# A positive feedback loop mediates crosstalk between calcium, cyclic nucleotide and lipid signalling in calcium-induced *Toxoplasma gondii* egress

**DOI:** 10.1371/journal.ppat.1010901

**Published:** 2022-10-20

**Authors:** Stephanie D. Nofal, Caia Dominicus, Malgorzata Broncel, Nicholas J. Katris, Helen R. Flynn, Gustavo Arrizabalaga, Cyrille Y. Botté, Brandon M. Invergo, Moritz Treeck

**Affiliations:** 1 Signalling in Apicomplexan Parasites Laboratory, The Francis Crick Institute, London, United Kingdom; 2 Protein Analysis and Proteomics Platform, The Francis Crick Institute, London, United Kingdom; 3 Apicolipid Team, Institute for Advance Biosciences, CNRS UMR5309, Université Grenoble Alpes, INSERM U1209, Grenoble, France; 4 University of Indianapolis, School of Medicine, Indianapolis, Indiana, United States of America; 5 Translational Research Exchange at Exeter, University of Exeter, Exeter, United Kingdom; Universite de Geneve Faculte de Medecine, SWITZERLAND

## Abstract

Fundamental processes that govern the lytic cycle of the intracellular parasite *Toxoplasma gondii* are regulated by several signalling pathways. However, how these pathways are connected remains largely unknown. Here, we compare the phospho-signalling networks during *Toxoplasma* egress from its host cell by artificially raising cGMP or calcium levels. We show that both egress inducers trigger indistinguishable signalling responses and provide evidence for a positive feedback loop linking calcium and cyclic nucleotide signalling. Using WT and conditional knockout parasites of the non-essential calcium-dependent protein kinase 3 (CDPK3), which display a delay in calcium inonophore-mediated egress, we explore changes in phosphorylation and lipid signalling in sub-minute timecourses after inducing Ca^2+^ release. These studies indicate that cAMP and lipid metabolism are central to the feedback loop, which is partly dependent on CDPK3 and allows the parasite to respond faster to inducers of egress. Biochemical analysis of 4 phosphodiesterases (PDEs) identified in our phosphoproteomes establishes PDE2 as a cAMP-specific PDE which regulates Ca^2+^ induced egress in a CDPK3-independent manner. The other PDEs display dual hydrolytic activity and play no role in Ca^2+^ induced egress. In summary, we uncover a positive feedback loop that enhances signalling during egress, thereby linking several signalling pathways.

## Author summary

The obligate intracellular parasite, *Toxoplasma gondii*, relies on successive rounds of invasion, intracellular growth and exit from the host cell to ensure its survival. Host cell exit, also known as egress, is a highly regulated process governed by several signalling pathways including cyclic nucleotide, phospholipid and calcium signalling. Despite recent advances in understanding how these signalling pathways mediate egress, it remains unclear how they interact with one another. Here, we use a combination of phosphoproteomics, lipidomics, reverse genetics and biochemical assays to demonstrate the presence of a feedback loop linking calcium signalling with the upstream cyclic nucleotide and phospholipid signalling pathways. This feedback loop appears to be regulated in part by a plant-like calcium dependent kinase (CDPK3) as well as a cAMP-specific phosphodiesterase (PDE2) and likely allows the parasite to integrate different stimuli to rapidly increase second messengers required for rapid egress.

## Introduction

The Apicomplexa are obligate intracellular parasites that pose a considerable challenge to human and animal health. The most prevalent member of this phylum, *Toxoplasma gondii*, infects virtually all warm-blooded animals, including an estimated 30% of humans worldwide [1]. To ensure its survival in the host, *Toxoplasma*, like all apicomplexan parasites, must actively invade host cells to initiate replication inside a parasitophorous vacuole. Following several rounds of division, or in response to adverse environmental changes, tachyzoites are triggered to egress from host cells, allowing for subsequent cycles of reinvasion of new host cells and growth.

At any stage during the replicative cycle, *T*. *gondii* may be triggered to egress from infected cells in response to deleterious environmental changes. Both extrinsic and intrinsic stimuli play a role in this process. Extrinsic signals include low potassium (K^+^), low pH, and serum albumin [2–4], while the accumulation of phosphatidic acid (PA) produced in the parasitophorous vacuole serves as an intrinsic signal to induce natural egress, although this occurs in a more gradual manner after several cycles of endodyogeny [5]. Once initiated, tachyzoites secrete vesicles, called micronemes, which contain proteins necessary for host cell attachment and invasion.

Irrespective of the egress trigger, it is clear that second messengers play a key role in driving the process forward once initiated. Indeed, calcium (Ca^2+^) [6], purine cyclic nucleotides (cyclic guanosine monophosphate (cGMP) and cyclic adenosine monophosphate (cAMP)) [4,7], and phosphatidic acid (PA) [8] have all been implicated (a model is shown in S1 Fig). Upon initiation of egress, migration, or invasion, cytosolic Ca^2+^ levels rise substantially [9]. It has been hypothesised that inositol triphosphate (IP_3_), generated by phosphoinositide phospholipase C (PI-PLC)-mediated cleavage of phosphatidylinositol 4,5-bisphosphate (PIP_2_), opens an (as yet unidentified) IP_3_-sensitive channel to release Ca^2+^ from organelles that otherwise sequester Ca^2+^ during immotile replicative stages [10,11]. Once released, Ca^2+^ activates a range of effectors, including a group of ‘plant-like’ Ca^2+^-dependent protein kinases (CDPKs) [12] and proteins involved in vesicle exocytosis [13]. PI-PLC-mediated cleavage of PIP_2_ also leads to the production of diacylglycerol (DAG), which can be converted to PA by the apicomplexan-specific DAG-kinase 1 (DGK1). In conjunction with Ca^2+^, PA is believed to play an indispensable role in microneme secretion by interacting with the PA receptor, acylated pleckstrin-homology (PH) domain-containing protein (APH), to facilitate microneme exocytosis [8].

The apicomplexan cGMP-dependent protein kinase (PKG) has been identified as a key regulator of the above-mentioned Ca^2+^ signalling cascade by facilitating the production of IP_3_ precursors [14]. Moreover, several studies have suggested that PKG, beyond its regulation of the phosphoinositide pathway, may also exert further control over egress by targeting as yet unidentified substrates required for microneme secretion [4,15]. In *T*. *gondii* tachyzoites, the cAMP-dependent protein kinase catalytic subunit 1 (PKAc1), meanwhile, has been proposed to act as a negative regulator of PKG signalling by inhibiting egress induced by parasite-dependent acidification [7,16]. PKAc1 has been suggested to down-regulate PKG activity by indirectly regulating the phosphorylation status of the putative cGMP phosphodiesterase, PDE2 [7]. Notably, a recent study has demonstrated that PDE2 displays exclusively cAMP-hydrolysing activity [17], suggesting that the model proposed by Jia *et al*. requires revision. It is important to note that the cAMP signalling pathway plays no role in regulating *P*. *falciparum* egress during the blood stages of infection, and instead appears to be important for mediating invasion [18–20].

Although many experimental observations place PKG [7,15] and phosphoinositide [8] signalling upstream of cytosolic Ca^2+^ flux (and by extension the activation of CDPKs) a further level of interaction between cGMP signalling and CDPK3 has become apparent [21]. CDPKs, comprised of a serine/threonine kinase domain fused to a calmodulin-like domain, belong to a superfamily of kinases that feature prominently in the Ca^2+^ signalling pathways of plants and some ciliates. Although *Toxoplasma* has numerous CDPK encoding genes [22], CDPK1 and CDPK3 have been most extensively studied. CDPK1 has been implicated in microneme exocytosis and the subsequent initiation of gliding motility [21], while CDPK3, which is not required for lytic growth, has been shown to be important for rapid Ca^2+^ ionophore-induced egress, where the addition of the calcium ionophore A23187 leads to the formation of lipid-soluble complexes with divalent cations and is thought to induce a discharge of Ca^2+^ from organelle stores into the cytosol, leading to concerted parasite exit from the host cell in seconds [21,23–25]. Intriguingly, while a marked delay in egress is evident when CDPK3 depleted/inhibited parasites are treated with Ca^2+^ ionophore, this phenotype is partially rescued when tachyzoites are induced to egress with PDE inhibitors such as zaprinast and BIPPO [21,26]. Both zaprinast and BIPPO, known inducers of *Toxoplasma* egress [21,27] are thought to induce an elevation in cytosolic cGMP levels by inhibiting cGMP hydrolysing PDEs, activating PKG activity via elevated cGMP levels. Accordingly, these findings suggest a compensatory role for PKG signalling in the absence of CDPK3.

While this compensatory mechanism has not been examined in any great detail, it is possible that PKG and CDPK3 substrate specificity may overlap. Multiple kinases converging on shared targets can provide multiple layers of regulation to a single pathway, and this is a known feature of nucleotide-activated kinases, including PKG [28]. Alternatively, it is plausible that BIPPO’s compensatory effects are explained by a more direct link between CDPK3 and PKG activity; if CDPK3 were to play a feedback-mediated role in the positive regulation of PKG signalling, pharmacological activation of PKG (e.g. by BIPPO/zaprinast treatment) would also diminish the requirement for CDPK3 during egress. Interestingly, this is reminiscent of the *P*. *falciparum* PfCDPK5, where the egress block of PfCDPK5-deficient parasites can be rescued by hyperactivation of PKG [29].

While the current literature forms a common understanding that CDPKs, PKG, PKA, lipid and second messenger signalling are important across lifecycle stages in *Toxoplasma* and *Plasmodium* species, how they are spatially and temporally regulated, how they intersect and how specific signalling outcomes are achieved is not well described. It is therefore important to understand how kinases and other signalling enzymes function, and how external and internal signals are integrated across the parasite’s lifecycle.

Here, we report on the phosphorylation, lipid and cyclic nucleotide signalling networks activated during the pharmacological induction of *Toxoplasma* tachyzoite egress using either A23187 or BIPPO. Collectively, our data highlights the presence of a positive feedback loop between A23187-regulated Ca^2+^ release and cyclic nucleotide signalling, which culminates in parasite egress. This mechanism appears to be regulated, at least in part, by CDPK3 and the cAMP-specific phosphodiesterase PDE2.

## Results

### Generation of calcium reporter lines to align BIPPO and A23187 signalling pathways

While it is clear that cGMP and calcium signalling play a paramount role in rapid parasite egress from the host cell, it is not clear how the signalling pathways converge and differ. The observation that induced cGMP signalling can only partially overcome the egress defect of parasite lines depleted of CDPK3 (a kinase shown to be important for rapid induced egress using the Ca^2+^ ionophore A23187), indicates that induced cGMP signalling can bypass elements of Ca^2+^ signalling. To first identify how cGMP and Ca^2+^ signalling converge and differ, we compared their phosphorylation dynamics using two activators of these pathways: BIPPO, a PDE inhibitor, and the calcium ionophore A23187.

**Fig 1:**
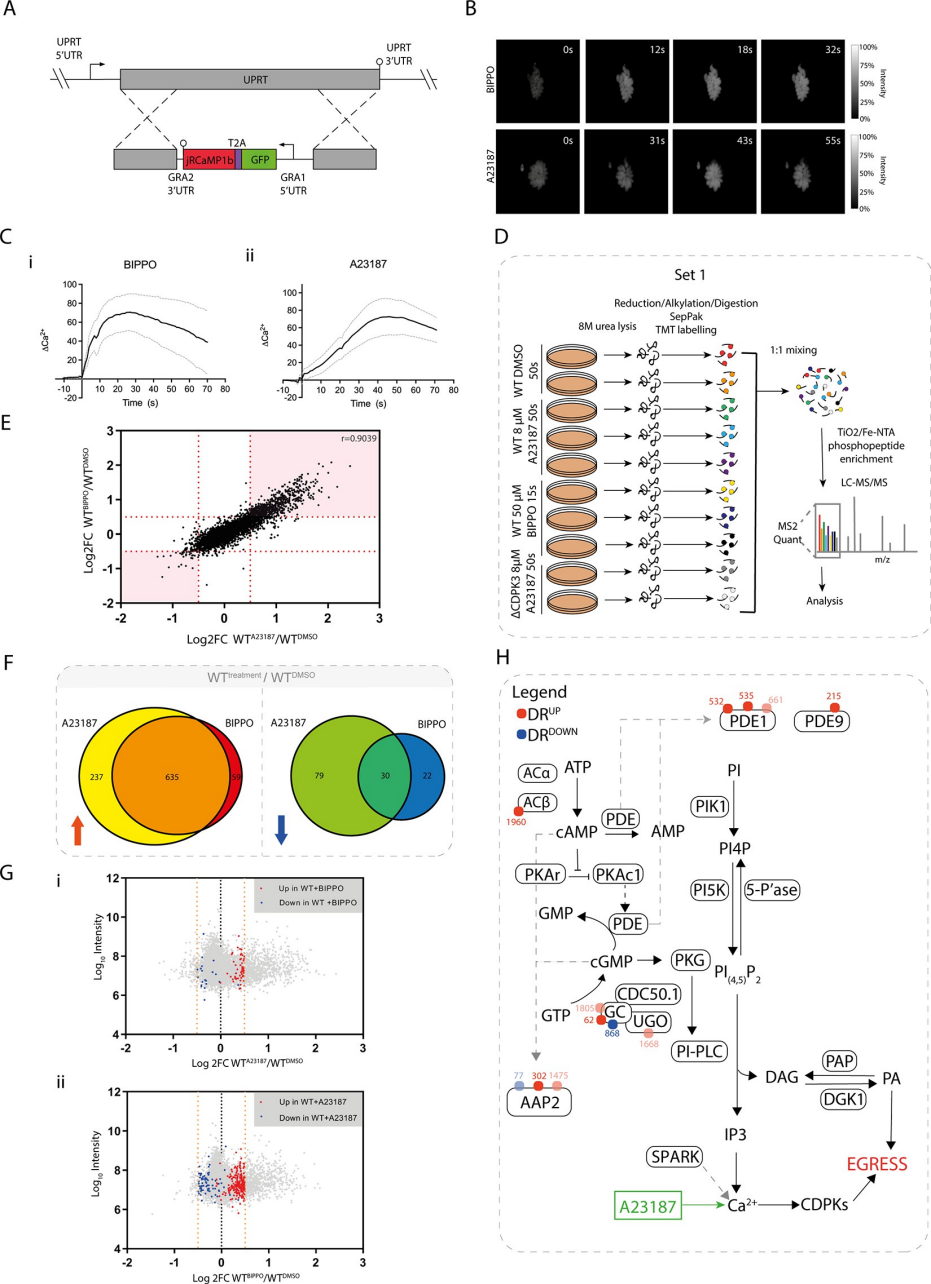
**Comparative phosphoproteomics of BIPPO- vs A23187-induced GFP-T2A-jRCaMP1b (WT) parasites at peak cytosolic calcium levels (A)** Generation of the calcium sensor line GFP-T2A-jRCaMP1b by integration into the UPRT locus. **(B)** Video microscopy of intracellular GFP-T2A-jRCaMP1b parasites (red channel) depicted in grey scale, following addition of 50 μM BIPPO or 8 μM A23187 at 0s. **(C)** Ratiometric tracking of mean Ca^2+^ response (jRCaMP1b/GFP normalised to 0), following addition of (i) 50μM BIPPO or (ii) 8 μM A23187. Grey dotted lines represent ± SEM. Data was collected from ≥ 10 vacuoles (in separate wells) over ≥6 days. **(D)** Schematic summary of the TMT-10-plex experiment (set 1) and workflow used to quantify the phosphoproteomes of intracellular WT tachyzoites treated with 50 μM BIPPO (15s) or 8 μM A23187 (50s). ΔCDPK3 parasites treated with 8 μM A23187 (50s) were included to facilitate later analyses. **(E)** Correlation of phosphosite regulation (log_2_FC) in BIPPO (y axis) and A23187 (x axis)-treated WT parasites. Each data point corresponds to a single phosphosite. Red dotted lines represent 3xMAD outlier thresholds used to determine differential site regulation. Phosphosites that fall within the pink shaded regions are differentially regulated upon treatment with both BIPPO and A23187. **(F)** Overlap between phosphosites that are differentially regulated in GFP-T2A-jRCaMP1b (WT) tachyzoites following treatment with 8 μM A23187 (50s) or 50 μM BIPPO (15s). Red arrow represents up-regulated sites (log_2_FC>0.5), blue arrow represents down-regulated sites (log_2_FC<-0.5). Only sites confidently detected in both treatment conditions were included for analysis. **(G)** Differential phosphosite regulation **(**log_2_FC) vs log_10_ total reporter intensity following treatment of WT tachyzoites with **(i)** 8 μM A23187 (50s) or **(ii)** 50 μM BIPPO (15s). Each data point corresponds to a single phosphosite. Only sites confidently detected in both BIPPO and A23187 samples are shown. In (i) Coloured data points highlight sites that were differentially up- or down-regulated (red and blue, respectively) upon BIPPO, but not A23187 treatment, while in (ii) coloured data points highlight sites that were differentially up- or down-regulated (red and blue, respectively) upon A23187, but not BIPPO treatment. Orange dotted lines represent 3xMAD outlier thresholds used to determine differential site regulation (log_2_FC>0.5 for up-regulated sites and log_2_FC<-0.5 for down-regulated sites). **(H)** Differentially A23187-regulated phosphosites detected on targets implicated in the regulation of cyclic nucleotides. Red and blue dots represent sites that are differentially up- or down-regulated, respectively. Numbers refer to site position within protein. Dots with reduced opacity represent phosphorylation sites with lower confidence in the phosphorylation site assignment.

The signalling kinetics following Ca^2+^ ionophore and BIPPO treatment vary, so we first determined a timepoint at which both pathways should be comparable. Common to both treatments is a rise in intracellular calcium levels before egress. We therefore chose peak intracellular calcium levels as a reference point to facilitate a direct comparison between BIPPO- and A23187-treated parasites. To this end, we generated a stable calcium sensor line that co-expresses, through use of a T2A ribosomal skip peptide, an internal GFP control and the genetically encoded ruby Ca^2+^ biosensor jRCaMP1b [30] from a single promoter (Fig 1A and 1B). The expression of the biosensor did not have any discernible effects on Ca^2+^ ionophore (A23187) or BIPPO induced egress rates (S2 Fig), therefore all subsequent experiments were performed with this line (henceforth referred to as wild type (WT)). While some variability of jRCaMP1b fluorescence was observed between vacuoles at a per-well level upon stimulation, ratiometric quantitation of jRCaMP1b fluorescence upon BIPPO or A23187 treatment of cytochalasin D-immobilised parasites illustrated distinct Ca^2+^ response curves; BIPPO treatment led to a rapid increase in Ca^2+^ levels, (Fig 1Ci), while the cytosolic Ca^2+^ rise detected upon A23187 treatment appeared more gradual (Fig 1Cii). To facilitate optimal alignment, and to account for the rapid kinetics of these signalling pathways, treatment timings of 15s (BIPPO) and 50s (A23187) were selected for subsequent phosphoproteomics experiments.

### A23187- and BIPPO-treated Wild Type parasites exhibit highly correlative phosphoproteomic responses at temporally aligned calcium flux

Having identified the optimal BIPPO and A23187 treatment timings to achieve maximal calcium release, we wanted to identify and compare phosphorylation events that take place during BIPPO- and A23187-induced signalling cascades at these timepoints. We used multiplexed tandem-mass-tags (TMT) and LC-MS/MS to quantify the phosphoproteome of intracellular WT tachyzoites treated with vehicle (DMSO), 50 μM BIPPO (15s) or 8 μM A23187 (50s) at 37°C. DMSO-treated parasite samples were generated in 2 biological replicates, while BIPPO- and A23187-treated samples were generated in 3 biological replicates (Fig 1D). At these timepoints all parasites remained intracellular. Samples were lysed, digested, and labelled with different TMT tags. Labelled samples were pooled and subjected to TiO_2_/Fe-NTA phosphopeptide enrichment prior to LC-MS/MS analysis. Of note, this experiment (set 1), also contains two biological replicates of an A23187-treated calcium-dependent kinase 3 deletion (ΔCDPK3) parasite line (S3A Fig). This allowed us to identify ΔCDPK3 dependency of signalling events during A23187 and BIPPO induced egress and is explained further below.

We quantified changes in phosphorylation states by calculating the log2-transformed intensity ratios (log2FC) of A23187- or BIPPO-stimulated WT parasites versus DMSO-treated WT parasites (S1 Data). In total we quantified 7,811 phosphorylation sites across these conditions.

Differentially regulated (DR) phosphorylation sites were selected if the log2FC exceeded 3x the median absolute deviation (MAD), rounded to the nearest tenth. This was log2FC>0.5 for up-regulated sites and log2FC<-0.5 for down-regulated sites; (S3B Fig) and applied across all datasets.

The rapid signalling progression upon treatment with BIPPO and A23187 inevitably results in variability in phosphosite intensities between replicates, where despite our best efforts, signalling may be stopped with several seconds difference between experiments. As such variability results in poor p-values in classical t-tests and, by extension, an under-reporting of true treatment-regulated sites, we did not subject DR sites to further p-value-based thresholding. However, the reporter intensities associated with DR sites correlated well across replicates (r>0.89, S3C Fig). This suggests that despite some of the aforementioned replicate variability, the overall trends across replicates were consistent, and these scores could therefore be confidently averaged to provide values that are representative of a site’s phosphorylation state at the timepoint of interest.

Comparison of the log2FCs observed in BIPPO- and A23187-treated samples shows strong correlation between the phosphorylation responses of these conditions (r = 0.9039, Fig 1E), suggesting that the signalling pathways at these selected timepoints align sufficiently well to directly compare them.

### Phosphoproteomic analysis cannot confidently distinguish BIPPO- from A23187-induced signalling

To investigate the signalling events that are shared between or are unique to BIPPO and A23187 treatment, we identified DR sites for each treatment condition. We then identified DR sites that were successfully quantified in both treatments, which allowed us to examine their behaviour under both conditions. In total we identified 746 BIPPO and 981 A23187 DR sites. A large overlap was detected between treatments for both up- and down-regulated phosphosites (DR^UP^ and DR^DOWN^, respectively); ~91% of phosphosites up-regulated following BIPPO treatment showed similar regulation upon A23187 addition and ~58% of BIPPO down-regulated sites behaved similarly following A23187 treatment (Fig 1F).

We also observed some dissimilar regulation between conditions; 59 phosphorylation sites were found to be up-regulated following BIPPO treatment only, while 237 sites were phosphorylated exclusively following A23187 treatment. Of the DR^DOWN^ phosphorylation sites, 22 were found to be unique to BIPPO treatment, while 79 were unique to A23187 treatment. These treatment-specific sites may originate from distinct signalling pathways, activated by each of the compounds. To discern whether these disparate site behaviours are truly treatment-specific effects, or whether they are the result of imperfect alignment of the treatment timings, we visualised phosphorylation site log2FCs following A23187 treatment, and highlighted phosphorylation sites that were only DR following BIPPO treatment (Fig 1Gi). Similarly, we also visualised phosphorylation site log2FCs following BIPPO treatment, and highlighted phosphorylation sites that were only DR following A23187 treatment (Fig 1 Gii). In both instances, most sites approached the DR thresholds for up- or down-regulation. While this does not preclude the possibility that some of the BIPPO- and A23187-specific DR^UP/DOWN^ sites are regulated in a drug-exclusive manner, it is likely that the majority of these sites would pass the DR threshold within seconds, and that minor changes in treatment timing can make the difference between surpassing the DR threshold or not.

Collectively, these findings demonstrate that at temporally aligned calcium release within the parasite, it is nearly impossible to detect clear signalling features that confidently distinguish the BIPPO-activated signalling pathway from the signalling cascade activated upon cytosolic Ca^2+^ elevation by A23187 treatment.

### A23187 treatment leads to differential regulation of proteins implicated in the PKG signalling pathway

A substantial overlap between BIPPO-regulated and A23187-regulated sites was expected given the increase of cytosolic Ca^2+^ in both treatment conditions. However, the inability to confidently distinguish BIPPO from A23187 signalling was unexpected, as these agents are believed to initiate egress by activating distinct, albeit interconnected, signalling responses [21]. Previous studies have placed PKG activation upstream of Ca^2+^ release [14,26] and it was therefore to our surprise that within the A23187 and BIPPO response overlap, DR phosphorylation sites were detected on proteins implicated in the catalysis and hydrolysis of the cyclic nucleotides (cNMPs) cGMP and cAMP, key molecules involved in PKG activation. Subsequent examination of all A23187-regulated phosphorylation sites (including those for which we lacked quantifications in BIPPO samples) identified differential phosphorylation on proteins including, but not limited to, enzymes important for cNMP signalling: PDEs (PDE1; TGGT1_202540 and PDE9; TGGT1_241880), the adenylyl cyclase beta (ACβ; TGGT1_270865) [7,31], a guanylyl cyclase (GC; TGGT1_254370) [5,31–33], the unique guanylyl cyclase organiser (UGO; TGGT1_238390 [5] and a cyclic nucleotide binding domain (CNBD) containing protein apical annuli protein 2 (AAP2; TGGT1_295850) [34] (Fig 1H).

The differential phosphorylation of several proteins in the upstream pathway of cNMP production/regulation hints at a putative Ca^2+^-mediated feedback loop that regulates cGMP and/or cAMP signalling. The existence of such a feedback mechanism could account for our inability to confidently discern PKG-specific signalling upon BIPPO treatment, as such signalling would be activated upon treatment with both BIPPO and A23187.

### Deletion of CDPK3 leads to signalling perturbations in both A23817 and BIPPO treatment conditions

To seek further evidence for or against a positive feedback loop, we set out to explore CDPK3’s role in A23187- and BIPPO-induced signalling. BIPPO/zaprinast-mediated activation of PKG partially compensates for a loss of CDPK3, which has led to the hypothesis that, given the function of both kinases in egress, the kinases’ substrate specificities may overlap [21]. In such a scenario, BIPPO treatment would facilitate PKG-mediated phosphorylation of CDPK3 targets, thus overcoming the egress delay otherwise seen in A23187-treated parasites.

**Fig 2:**
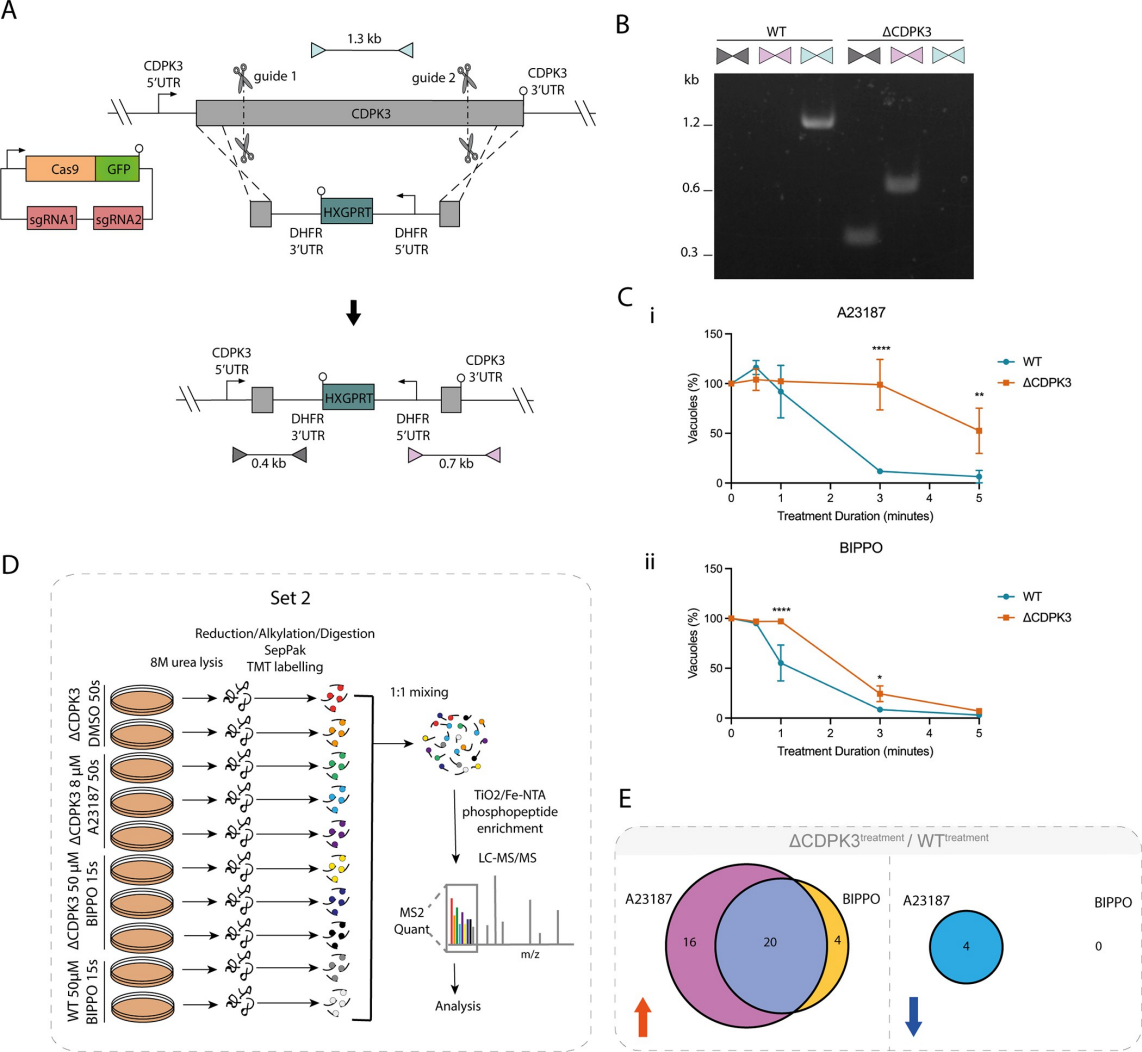
**Comparative phosphoproteomics of BIPPO vs A23187-induced GFP-T2A-jRCaMP1b ΔCDPK3 parasites at peak cytosolic calcium levels (A)** Generation of the GFP-T2A-jRCaMP1b ΔCDPK3 line using CRISPR/Cas9 to increase site-directed integration. Scissors represent Cas9 cleavage sites and lollipops depict stop codons. Coloured triangles represent primer pairs used to detect WT, 5’ integration and 3’ integration loci (light blue, grey and pink respectively). **(B)** PCR results using the primer pairs shown in Fig 2A **(C)** Egress assay of GFP-T2A-jRCaMP1b (WT) and GFP-T2A-jRCaMP1b ΔCDPK3 (ΔCDPK3) parasites following treatment with **(i)** 8 μM A23187 or **(ii)** 50μM BIPPO. Graph shows the remaining % of un-egressed vacuoles (relative to untreated) following A23187/BIPPO treatment. Data are represented as mean ± s.d. (n = 3). Two-way ANOVA with Holm-Sidak *post hoc* comparison.****, *P* ≤ 0.0001 **(D)** Schematic summary of the TMT-10-plex experiment (set 2) and workflow used to quantify the phosphoproteomes of intracellular ΔCDPK3 tachyzoites treated with 50 μM BIPPO (15s), 8 μM A23187 (50s), and WT parasites treated with 50 μM BIPPO (15s). **(E)** Overlap between differentially regulated phosphosites that display CDPK3 dependency following treatment with 50 μM BIPPO (15s) or 8 μM A23187 (50s) (data derived from set 1 and 2). Red and blue arrows represent up- and down-regulated sites, respectively. Only phosphosites found to be differentially regulated upon treatment with both A23187 and BIPPO were included for analysis.

To identify phosphorylation sites that might fit such criteria, we wanted to identify phosphorylation sites that are CDPK3-dependent upon A23187s treatment, but CDPK3-independent upon BIPPO treatment. To do this, we generated a ΔCDPK3 parasite line by replacing the endogenous CDPK3 locus in the RH GFP_T2A_jRCaMP1b line with a HXGPRT expression cassette (henceforth known as ΔCDPK3) (Fig 2A) and confirmed deletion of CDPK3 by PCR (Fig 2B). We performed an egress assay to validate the known A23187-induced egress delay reported for ΔCDPK3 parasites [21,23–25]. As expected, we found that A23187-induced egress was substantially inhibited in this line (Fig 2Ci), while a less severe egress delay was observed in BIPPO-treated ΔCDPK3 parasites (Fig 2Cii). This recapitulates previous findings [21,26] that activation of PKG partially compensates for a loss of CDPK3.

We next quantified phosphorylation events in ΔCDPK3 parasites treated with DMSO, 50 μM BIPPO (15s) or 8 μM A23187 (50s) at 37°C in biological replicates (2x DMSO, 3x A23187, 3x BIPPO) (Fig 2D). In this experiment (set 2), we included 2 biological replicates of BIPPO-treated WT parasites (S3A Fig). In conjunction with the ionophore-treated ΔCDPK3 parasites included in set 1 (Fig 1D), this allowed us to identify CDPK3-dependent phosphorylation sites during BIPPO- and A23187-induced egress (S2 Data). We first identified DR phosphorylation sites across all datasets for which we had quantifications and tested for CDPK3 dependency. Of the 498 phosphosites detected, 44 sites (~8.5%) were CDPK3-dependent (Fig 2E and S2 Data). 40 sites were classed as DR^UP^; 16 were exclusive to A23187 treatment, 20 were identified upon both A23187 and BIPPO treatment, and 4 were detected upon BIPPO treatment only. By contrast, only 4 sites were classed as DR^DOWN^, all in a seemingly A23187-exclusive manner.

The 16 phosphorylation sites that show CDPK3 dependency exclusively upon A23187 treatment constituted putative candidates for PKG/CDPK3 substrate overlap. We reasoned that, if a DR^UP^ site was found to be CDPK3-dependent upon A23187 treatment only, this phosphorylation site should be recovered in ΔCDPK3 parasites following BIPPO treatment. Only 3 of these phosphorylation sites showed this behaviour and were located on two hypothetical proteins (TGGT1_243460, TGGT1_232120) and a DnaJ domain-containing protein (TGGT1_203380) (S2 Data). While these findings do not rule out the ‘substrate overlap’ theory to account for BIPPO’s compensatory effects, the putative overlap is extremely small, and none of these proteins contain predicted domains that would explain the rescue of CDPK3 mutants by BIPPO-induced egress.

In conjunction with the observed A23187- and BIPPO-driven phosphorylation of proteins implicated in the catalysis and hydrolysis of the cyclic nucleotides cGMP and cAMP (key signalling molecules involved in the regulation of PKG activation), our current findings provide stronger evidence for a Ca^2+^-regulated feedback loop that acts as a signal amplifier in both A23187- and BIPPO-induced egress.

### A sub-minute timecourse of CDPK3 dependent and independent signalling progression in ionophore-induced egress

**Fig 3:**
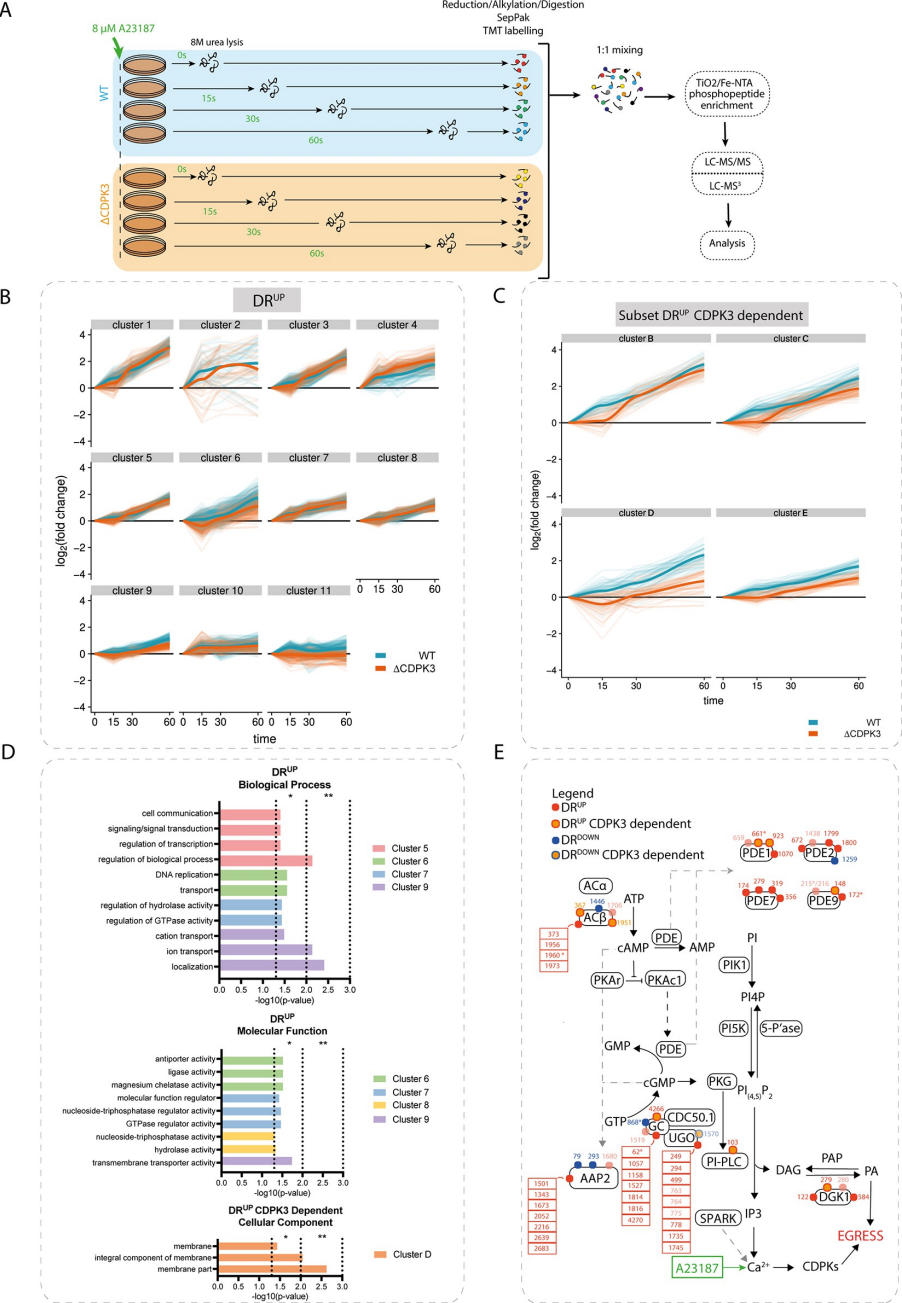
**A23187 treatment results in partially CDPK3-dependent phosphorylation of targets implicated in PKG signalling (A)** Schematic of A23187-treatment timecourse experimental design. **(B)** Gaussian mixture-model-based clustering of all DR^UP^ sites in the A23187-treatment timecourses. Log_2_FC values from both WT and ΔCDPK3 samples were combined to cluster on six dimensions (WT 15s, 30s and 60s and ΔCDPK3 15s, 30s and 60s). Thin lines represent the timecourse traces of individual phosphorylation sites. Thick lines represent Loess regression fits of all traces. **(C)** Gaussian mixture-model-based clustering of a subset of DR^UP^ CDPK3-dependent sites in the A23187-treatment timecourses. Log_2_FC values from both WT and ΔCDPK3 samples were combined to cluster on six dimensions (WT 15s, 30s and 60s and ΔCDPK3 15s, 30s and 60s). Thin lines represent the timecourse traces of individual phosphorylation sites. Thick lines represent Loess regression fits of all traces. Four clusters (B-E) best illustrating the transient phosphorylation delay in ΔCDPK3 parasites are shown. **(D)** GO term enrichment analysis showing a subset of terms in the DR^UP^ and DR^UP^ CDPK3-dependent clusters. **(E)** Differentially A23187-regulated phosphosites detected on targets implicated in the regulation of PKG signalling. Red and blue dots represent sites that are differentially up- or down-regulated, respectively. Dots with an orange centre indicate CDPK3-dependent sites. Numbers refer to site position within protein. Dots with reduced opacity represent phosphorylation sites with lower confidence in the phosphorylation site assignment. Asterisks highlight sites that were previously detected in this study’s A23187/BIPPO phosphoproteome experiments (see Figs 1D and 2D).

While the experiments above provide strong support for a feedback loop, they represent only a snapshot of signalling at a single timepoint. To further examine the cNMP-induced signalling pathways in WT and ΔCDPK3 mutants following A23187 treatment, we performed a sub-minute phosphosignalling timecourse. We treated intracellular WT and ΔCDPK3 tachyzoites with 8 μM A23187 for 15, 30 or 60 seconds at 37°C (Fig 3A), during which the parasites remained intracellular. As before, samples were subjected to TMT-based quantitative analysis of phosphoproteomic changes. Fold changes were calculated relative to a 0s (DMSO) control. In total we quantified 11,021 phosphorylation sites (S3 Data). It is possible that some changes in phosphopeptide abundance could reflect changes in protein abundance. However, for the vast majority of DR phosphorylation sites found on a given protein, we found other phosphorylation sites that do not change throughout our timecourse. This suggests that any observed changes are dynamic changes in phosphorylation and not protein levels.

DR thresholds were set at 3x MAD of the log2FC across each WT timepoint (15s, 30s and 60s). Phosphorylation sites were considered differentially regulated if at any given timepoint their log2FC surpassed these thresholds. CDPK3 dependency was determined for each phosphorylation site by calculating the log2 ratios of A23187-treated WT and ΔCDPK3 parasites for each timepoint. The resulting ratios were used to calculate the MAD at each timepoint and the most stringent score was used to set 3X MAD outlier thresholds (i.e. CDPK3 dependency thresholds). Phosphorylation sites were considered to be CDPK3 dependent if, at any given timepoint, they showed A23817 specific phosphorylation upon A23187 treatment in WT but not ΔCDPK3 parasites.

We identified 2,405 phosphorylation sites (DR^UP^) upon A23187 treatment in WT parasites, which were also quantified in ΔCDPK3 parasites (S3 Data). To examine whether this dataset recapitulates our previous findings, we compared the DR^UP^ sites identified at the 60s timepoint in this experiment, with those identified after 50s A23187 treatment from the preceding experiments (Figs 1D and 2D). Of the 572 DR phosphorylation sites identified 50s after A23187 treatment in our initial experiment, 503 sites (~88%) also passed the threshold for differential (up)regulation in the timecourse at the 60s post-treatment timepoint. Reassuringly, we observed many proteins previously identified as being phosphorylated in a CDPK3-dependent manner [35,36], including the CRAL/TRIO domain containing protein (TGGT1_254390), a putative P-type ATPase4 (TGGT1_278660), CDPK2A (TGGT1_206590) and the tyrosine transporter ApiAT5-3 (TGGT1_257530).

To get an overview of the progression of signalling cascades in DMSO- and A23187-treated parasites, we performed a clustering analysis, as previously described [37], of DR phosphorylation sites identified in WT and, separately, of DR sites found to be CDPK3-dependent in our timecourse experiments (see S1 Table for all clusters). We obtained 11 clusters showing distinct up-regulation dynamics (Fig 3B) and 10 clusters showing down-regulation dynamics in WT parasites (S4A Fig). Analysis of CDPK3-dependent DR sites, meanwhile, yielded 10 up-regulated clusters (Figs 3C and S4B) and 6 down-regulated clusters (S4C Fig).

In the up-regulated clusters, we identified a preponderance for phosphorylation motifs with arginine in the -3 position (S4Di Fig), a consensus sequence that has previously been shown to be preferentially phosphorylated by CDPK1 [38] and possibly CDPK3 [35]. Reassuringly, this consensus motif was also observed among DR^UP^ CDPK3-dependent sites (S4Dii Fig). Down-regulated phosphorylated sites, meanwhile, show a clear enrichment for proline in the +1 position (S4Diii and S4Div Fig). This indicates that while CDPK activity (and/or activity of kinases with a similar substrate preference) is being induced by calcium signalling, a distinct set of one or more kinases with this phosphorylation motif preference is being inactivated concurrently. Alternatively, this could be mediated by the activation of a specific phosphatase. We observed that several of the less phosphorylated proteins in the timecourse are secreted into the parasitophorous vacuole (PV), which physically separates the parasite from the host cell cytoplasm. Several proteins that are secreted into the PV have been shown to play a role in mediating egress. This includes GRA41, which has been shown to be important for A23187-induced egress [39]. It is therefore possible that the secreted proteins identified in our timecourse may be implicated in wider signalling events occurring in the PV that are required for egress.

Several functionally related proteins were phosphorylated with similar dynamics, as revealed by Gene Ontology (GO) term enrichment (see S2–S5 Tables for DR^UP^, DR^DOWN^, CDPK3-dependent DR^UP^ and CDPK3-dependent DR^DOWN^ GO term enrichments, respectively). Most notably, numerous up-regulation clusters were enriched in terms related to signal transduction (GO:0007165, GO:0007154, GO:0023052) and hydrolase activity (GO:0016787, GO:0042578, GO:0016462, GO:0016817, GO:0016818, GO:0017111), respectively (Fig 3D). These enrichments were, in part, driven by phosphorylation of PDEs and cyclases involved in cyclic nucleotide signalling. Thus, not only are the enzymes potentially upstream of PKG being phosphorylated upon exposure to ionophore, but the dynamics of phosphorylation are also similar between them.

We also found significant enrichment of membrane proteins in CDPK3-dependent clusters (GO:0044425, GO:0016021, GO:0031224, GO:0016020) (Fig 3D). These are predicted to play roles in nutrient transport and ion-exchange, including the sodium-hydrogen exchangers NHE1 and NHE3 (which have previously been linked to egress) [40,41], and the tyrosine transporter ApiAT-5-3 (a known target of CDPK3 phosphorylation) [35,36].

Further GO enrichment analysis of up-regulated clusters revealed other potential downstream targets of ionophore-induced signalling, including transcription (e.g. GO:0006355, GO:1903506, GO:2001141) by AP2-family transcription factors, magnesium chelatase activity (GO:0016851) by DNA replication licensing factors, and regulation of a GTPase-mediated process (GO:00423087, GO:0051336) (Fig 3D). Intriguingly, we found that phosphorylation of several targets associated with these GO terms also showed CDPK3 dependency, including AP2VIII-2 (TGGT1_233120), AP2XI-2 (TGGT1_310900), MCM7 (TGGT1_237220), and a predicted GTPase, Ras-related Rab11 (TGGT1_289680). The function of these proteins in the CDPK3 signalling cascade is less clear but may point towards biological processes related to DNA replication and transcription.

In addition to its likely involvement in the numerous signalling processes mentioned above, CDPK3 also directly or indirectly regulates the activities of other signalling proteins. For example, CDPK3-dependent down-regulation was detected at a site within the protein kinase domain of the cell-cycle-associated protein kinase GSK (TGGT1_265330; S214). Similarly, CDPK3-dependent DR^UP^ sites were found within the PI3Ka domain of PI3/4-kinase (TGGT1_215700) and the EF-hand domains of centrin 2 (TGGT1_250340) and the calmodulin-like protein TgELC2 (TGGT1_305050) [42].

It is important to note that visualisation of the CDPK3-dependent DR^UP^ clusters revealed that for many sites, the effect of CDPK3 deletion was temporary, such that there was an initial delay in phosphorylation, but by 60s the sites reached only slightly lower log2FC values than in WT (Fig 3C). This may point to the redundant or compensatory activity of a protein kinase other than PKG, which could in part account for the fact that egress still occurs in CDPK3-depleted parasites, albeit at a delayed pace. Such a delay was not clearly detectable in the CDPK3-dependent DR^DOWN^ clusters.

The GO terms “signal transduction” and “hydrolase activity” mentioned above contained numerous phosphorylation sites on proteins potentially involved in cNMP signalling, including PDE1 and PDE9, the ACβ, the GC, UGO and the CNBD containing protein AAP2 (Fig 3E and S3 Data). In addition to these previously detected targets, we also observed increased phosphorylation of PDEs TGGT1_280410 (PDE7) and TGGT1_293000 (PDE2) as well as components of phosphoinositide signalling including the PI-PLC (TGGT1_248830) [8,43] and the DAG kinase 1 (DGK1; TGGT1_202460) [8]. Many, but not all, phosphorylation sites on these enzymes are found to be CDPK3-dependent, indicating that the putative feedback loop is likely regulated by several kinases (Fig 3E). PI-PLC is required for the production of IP_3_ which triggers calcium release and has been implicated as a key downstream mediator of PKG activity [14], while the DGK1 has been shown to play an important role in the conversion of intracellular DAG to PA and, by extension, the activation of microneme secretion and subsequent egress [8]. Collectively, these findings further substantiate our hypothesis that the A23187-mediated release of Ca^2+^ activates a feedback loop that is partially dependent on CDPK3 and regulates the PKG signalling pathway.

### Disruption of calcium signalling leads to perturbations in cAMP levels and lipid signalling following A23187 treatment

While it is uncertain whether the above-mentioned phosphorylation events modulate the function of the proteins that they are found on, the putative Ca^2+^-dependent regulation of cNMP signalling is intriguing as altered hydrolysis of either cAMP or cGMP has defined regulatory consequences for PKG activation. Similarly, both PI-PLC and DGK1 are key players in the lipid signalling pathway, acting downstream of PKG to modulate egress [8,14]. We next set out to test the importance of these phosphorylation events by testing whether disrupting calcium signalling, by deletion of CDPK3, would lead to dysregulation of these signalling pathways.

**Fig 4:**
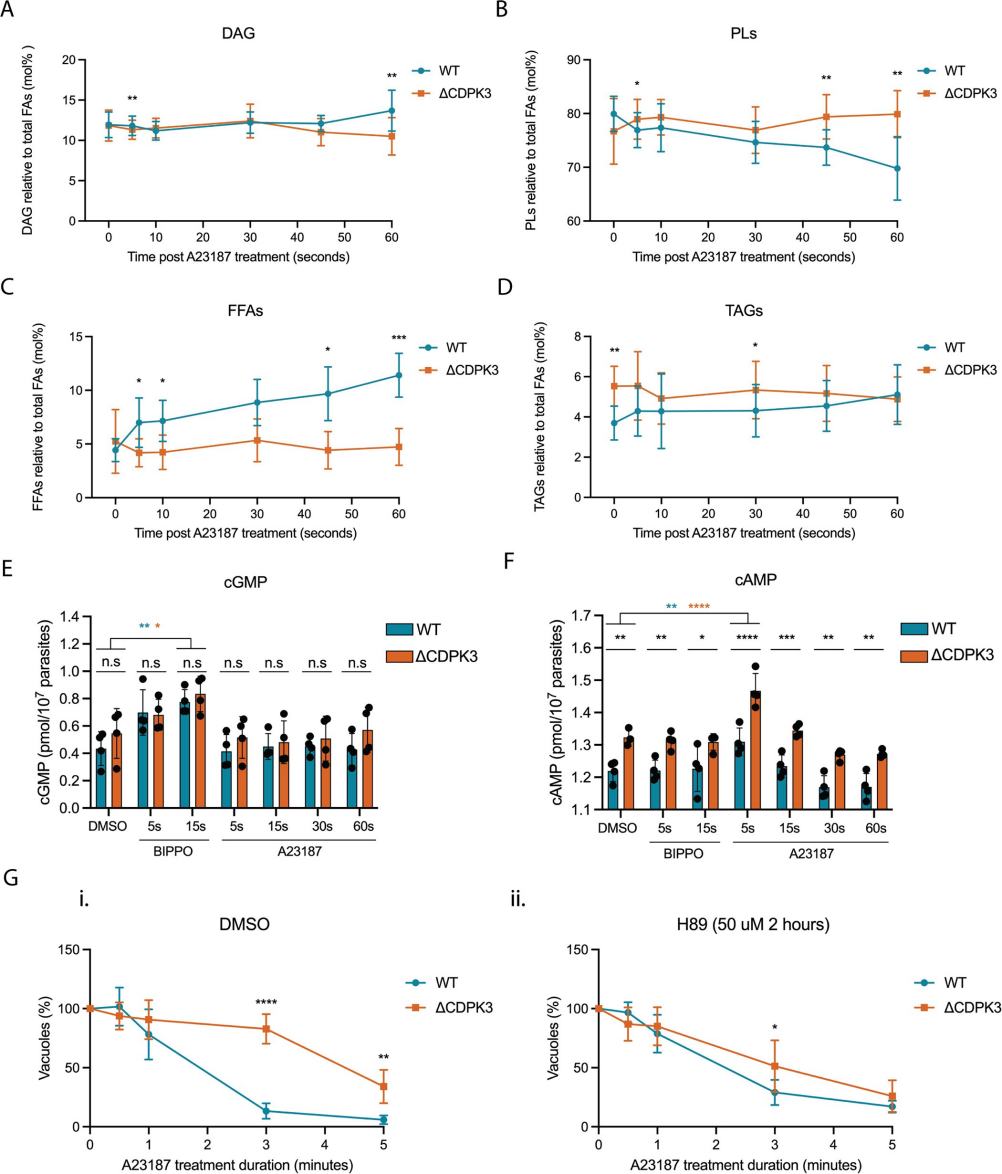
**Disruption of CDPK3 leads to perturbations in lipid and cAMP signalling following A23187 treatment** Pulse experiment of WT and ΔCDPK3 parasites treated with DMSO (0s) or 8 μM A23187 for 5, 10, 30, 45 or 60 seconds analysing levels of (A) DAG, (B) PLs, (C) FFAs and (D) TAGs, with data expressed as %mol relative to total FAs. Data are represented as mean ± s.d. (n = 4). Significance between WT and ΔCDPK3 parasites was assessed using a paired two sample t-test. ***, *P* ≤ 0.001; **, *P* ≤ 0.01; *, *P* ≤ 0.05. **(E)** Comparison of intracellular cGMP and **(F)** cAMP levels in WT and ΔCDPK3 tachyzoites following treatment with DMSO for 60 seconds; 50 μM BIPPO for 5 or 15 seconds; or 8 μM A23187 for 5, 15, 30 or 60 seconds. All samples were lysed in 0.1 M HCl to inactivate all PDEs and cyclases, and extracts were analysed by using commercial ELISA-based cGMP and cAMP detection assays. Data are represented as mean ± s.d. (n = 4). Two-way ANOVA with Sidak multiple comparisons. ****, *P* ≤ 0.0001; ***, *P* ≤ 0.001; **, *P* ≤ 0.01; *, *P* ≤ 0.05; n.s, not significant. **(G)** Egress assay of WT and ΔCDPK3 parasites following treatment with 8 μM A23187. Parasites were pre-treated with either **(i)** DMSO or **(ii)** H89 before addition of A23187. Data are represented as mean ± s.d. (n = 5). Two-way ANOVA with Holm-Sidak *post hoc* comparison. ****, *P* ≤ 0.0001; **, *P* ≤ 0.01.

To this end, we analysed DAG and phospholipid levels in WT and ΔCDPK3 parasites before and after stimulus with the calcium ionophore A23187. To avoid inclusion of host cell-derived lipids, parasites were syringe lysed in DMEM [44]. Extracellular WT and ΔCDPK3 parasites were shifted to 37°C for 60 seconds to acclimatise, and then stimulated by addition of media containing A23187 similar to other ongoing studies [44]. The parasites were incubated with 8 μM A23187 for 5, 10, 30, 45 or 60 seconds before quenching to stop the signal chain, followed by lipid analysis. A 0s (DMSO) control was also included. The starting levels of DAG did not differ between WT and ΔCDPK3 parasites, however after 60 seconds of A23187 stimulus, WT parasites produced slightly more DAG than ΔCDPK3 parasites (Figs 4A and S5A). Accordingly, WT parasites began to show less phospholipid abundance than ΔCDPK3 parasites after 45 seconds of stimulus (Figs 4B and S5A), consistent with a lack of turnover of phospholipids to produce DAG in the ΔCDPK3 parasites. While our timecourse phosphoproteome data suggests that DAG metabolism-related proteins were the primary metabolic proteins affected in ΔCDPK3 parasites, we also identified other DR proteins involved in palmitoylation and triacylglycerol synthesis. This included the palmitoyltransferase DHHC13 (TGGT1_249380) [45] and TgLIPIN (TGGT1_230690) [46]. Accordingly, we also investigated Free Fatty Acids (FFAs) and triacylglycerol (TAG) levels. We observed a trend towards an increase in the levels of free fatty acids (FFAs) in WT parasites following A23187 stimulus (Figs 4C and S5B), which remained unchanged in ΔCDPK3 parasites. This was accompanied by a concomitant change in triacylglycerols (TAGs) whereby, prior to stimulus, ΔCDPK3 parasites had more TAGs than WT parasites, but after A23187 stimulus, WT tachyzoites produced more TAGs over time so that levels became similar between both parasite lines (Figs 4D and S5C).These findings demonstrate that, following A23187 treatment, ΔCDPK3 parasites have altered FFA and TAG abundance necessary for lipid recycling and storage, consistent with a speculated role for CDPK3 in metabolic regulation [35]. A full lipidomic analysis of individual phospholipid species including lysophosphatidylinositol (LPI), lysophosphatidylcholine (LPC) phosphatidylinositol (PI), phosphatidylserine (PS), phosphatidylthreonine (PT) phosphatidicacid (PA), phosphatidylethanolamine (PE), FFAs, sphingomyelin (SM) and phosphatidylcholine (PC) found no significant difference in any phospholipids between WT and ΔCDPK3 parasites under normal cell culture conditions (S5D Fig), showing that defects in lipid signalling in ΔCDPK3 parasites can only be seen following A23187 calcium stimulus.

Collectively, these findings suggest that A23187-induced changes in the phosphorylation status of enzymes involved in lipid signalling leads to alterations in DAG, phospholipid, TAG and FFA levels following stimulation with A23187.

As our phosphoproteome timecourse also demonstrated that cNMP-related proteins were differentially phosphorylated in ΔCDPK3 parasites shortly after A23187 treatment, we next explored whether disrupting calcium signalling, by means of CDPK3 deletion, would lead to changes in cyclic nucleotide levels. To mimic the intracellular conditions of the parasites used in our phosphoproteome experiments, while ensuring the absence of host-derived cNMPS, we measured intracellular levels of cAMP and cGMP in WT and ΔCDPK3 parasites syringe lysed in Endo buffer [6], a potassium-rich buffer which mimics the intracellular environment. Following lysis, parasites were treated with vehicle (DMSO), 50 μM BIPPO for 5 or 15 seconds or 8 μM A23187 for 5, 15, 30 or 60 seconds at 37°C. Basal levels of cGMP were identical in WT and ΔCDPK3 parasites and, as expected, treatment with the PDE inhibitor BIPPO resulted in elevated cGMP levels in both parasite lines compared to baseline (Fig 4E). In contrast, and somewhat surprisingly, we observed no changes in cGMP levels following A23187 treatment over the course of 60 seconds. This is in contrast to what has been previously described by Stewart *et al.*, who observed a rise in cGMP levels following A23187-treatment of extracellular parasites [26]. To test whether this discrepancy is due to an altered calcium response when parasites are in Endo buffer, we measured the calcium flux of extracellular parasites in Endo buffer following treatment with either BIPPO or A23187. While BIPPO treatment led to a response similar to that of intracellular parasites (S6A Fig), parasites in Endo buffer had a reduced Ca^2+^ flux following A23187 treatment compared to intracellular parasites (S6B Fig). Addition of exogenous CaCl_2_ to Endo buffer restored Ca^2+^ flux intensity (S6C Fig), however this did not lead to a change in cGMP levels following A23187 treatment (S6D Fig), suggesting that other factors, such as the type of assay used to measure cGMP levels, are responsible for the differences between our results and those reported by Stewart *et al.*.

Basal cAMP levels were 8.3% higher in knockout parasites compared to WT. Following treatment with A23187, we found that cAMP levels initially rose in both WT and knockout, with a gradual decrease to below basal levels at 60 seconds post treatment (Fig 4F). For both WT and ΔCDPK3 parasites, the A23187-induced rise in cAMP levels was only significant 5 seconds post treatment when compared to basal levels. We observed no immediate change in cAMP levels following BIPPO treatment, suggesting that BIPPO does not inhibit cAMP-specific PDEs in *T*. *gondii*. These findings point towards perturbations in cAMP signalling in ΔCDPK3 parasites which have elevated basal levels that further increase upon A23187 treatment.

Since basal cAMP levels are elevated in ΔCDPK3 parasites compared to WT, we reasoned that inhibition of cAMP signalling would overcome the A23187-mediated egress delay observed in knockout parasites. To test this, we treated both WT and ΔCDPK3 parasites with the ATP-competitive PKAc inhibitor H89 (50 μM). After 2 hours of treatment, there was a significant amount of premature egress in both WT and ΔCDPK3 parasites (~45% & ~34%, respectively) (S5E Fig) consistent with previous reports that have shown that the downregulation of cAMP signalling by genetic disruption of PKAc1 stimulates premature egress in *Toxoplasma* [7,16]. Intriguingly, when we investigated A23187-induced egress rates of the remaining intracellular H89 pre-treated parasites, we found that the egress delay normally observed in ΔCDPK3 parasites was largely rescued with H89 pre-treatment (Fig 4Gi-ii). This finding suggests that pharmacological inhibition of cAMP signalling is sufficient to partially compensate for the deletion of CDPK3 and that cAMP signalling is an important part of the feedback loop.

### Cell biological and biochemical characterisation of PDE1, 2, 7 and 9

**Fig 5:**
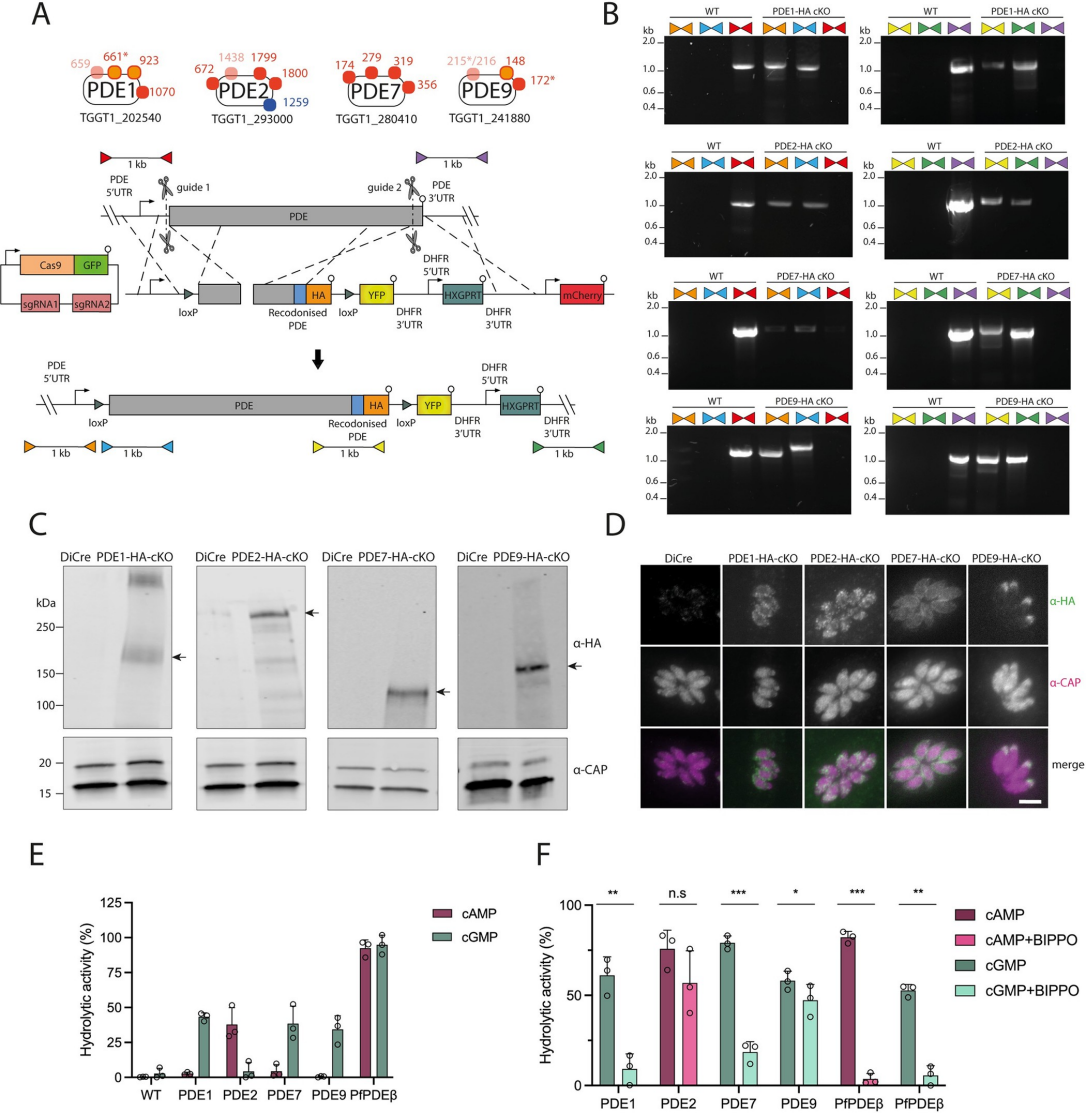
**Candidate PDEs occupy distinct subcellular localisations and are differentially inhibited by BIPPO (A)** Schematic representation of the PDEs identified in the timecourse phosphoproteome (see Fig 2E) and the strategy used to generate the conditional PDE knockout lines. CRISPR/Cas9 was used to generate two cuts in the gene and two separate repair templates were provided to integrate one loxP site (green triangle) upstream of the PDE gene, and another repair template to tag the PDE with a C-terminal HA epitope tag (orange) and introduce a second loxP site, a YFP sequence and the HXGPRT cassette. Scissors represent Cas9 cleavage sites and lollipops depict stop codons. Coloured triangles represent primer pairs used to detect WT, 5’ integration and 3’ integration loci for 5’ loxP integration (red, orange and blue respectively) and 3’ tagging (purple, yellow and green respectively). **(B)** PCR analysis showing correct 5’ and 3’ integration of the 5’ loxP site and 3’ tagging repair templates, and lack of WT locus in PDE1, PDE2, PDE7 and PDE9 cKO lines. Coloured triangles represent primer pairs as illustrated in Fig 5A **(C)** Western blot analysis of parental DiCre and HA-tagged PDE1, PDE2, PDE7 and PDE9 cKO parasites probed with α-HA antibodies showing migration of the PDEs at their expected molecular weights as depicted by arrows. A non-specific band >250 kDa is observed in the PDE1-HA cKO line. Blots were probed with α-CAP antibodies as a loading control. **(D)** Immunofluorescence analysis of DiCre and HA-tagged PDE1, PDE2, PDE7 and PDE9 cKO lines probing with α-HA (red) and α-CAP (green) antibodies. Scale bar, 5 μm. **(E)** Hydrolytic activity of immunoprecipitated HA-tagged PDE1, PDE2, PDE7, PDE9 and PfPDEβ using either 1 μM cAMP or 10 μM cGMP as a substrate and incubating at room temperature for 1 hour. Lysates from the WT parental line were also included as a control. Data are represented as mean ± s.d. (n = 3). **(F)** Hydrolytic activity of immunoprecipitated HA-tagged PDE1, PDE2, PDE7, PDE9 and PfPDEβ after incubating with DMSO (vehicle) or 25 μM BIPPO. 1 μM cAMP was used as a substrate for PDE2 and PDEβ, while 10 μM cGMP was used as a substrate for PDE1, PDE7, PDE9 and PDEβ. Data are represented as mean ± s.d. (n = 3). Significance was assessed using a Two-way ANOVA. ***, *P* ≤ 0.001; **, *P* ≤ 0.01; *, *P* ≤ 0.05; n.s. not significant.

The preponderance of A23187 and BIPPO-induced phosphorylation of several PDEs, ACβ and the GC suggests that they may play an important role in the cAMP- and cGMP-mediated signalling cascades that lead to egress assuming that phosphorylation may directly, or indirectly control their activity. Given the increase of cAMP, and its role in inhibiting egress, we predicted that one or more cAMP metabolising enzymes might play an important role in the feedback loop. These enzymes could either be PDEs that hydrolyze cAMP, or one or both of the predicted ACs in *Toxoplasma*, which generate cAMP. While we observed differential phosphorylation of ACβ (both CDPK3 dependent and independent), both ACα and ACβ, the only two predicted ACs encoded in the *Toxoplasma* genome, have previously been shown to be non-essential for the lytic cycle, suggesting these proteins can compensate for the loss of one another [7]. Accordingly, we decided to further explore the 4 PDEs that were differentially phosphorylated following A23187 egress stimulation. Some, but not all, of these differentially phosphorylated sites were CDPK3 dependent. In order to study these 4 PDEs in the lytic cycle and determine which are capable of hydrolysing cAMP, we generated HA-tagged conditional knockouts (cKOs) of each of the PDEs (Fig 5A). For each line, integration of both repair templates was validated by PCR (Fig 5B). Western blot analysis confirmed that they migrate at their predicted sizes (Fig 5C), and we found that each PDE occupies a distinct subcellular localisation (Fig 5D), in agreement with previous reports [17,47]. To identify the substrate specificity of the PDEs, we immunoprecipitated each PDE via the HA-tag from parasite lysates (S7A Fig) and measured their hydrolytic activity *in vitro*. We included the *P*. *falciparum* PDEβ that was previously shown to be dual-specific as a positive control [19]. After incubating the PDEs for 1 hour at room temperature with either 1 μM cAMP or 10 μM cGMP, PDEs 1, 7 and 9 were able to hydrolyse cGMP, while PDE2 is specific for cAMP (Fig 5E). Only PfPDEβ displayed dual-hydrolytic activity in our hands. Our results differ from the recent findings of Moss *et al*. and Vo *et al*. in which PDE1 and PDE9 were found to display dual hydrolytic activity, respectively [17,47]. To investigate the disparities between these results, we re-examined the hydrolytic activity of our PDEs using the conditions outlined by Moss *et al*., which involved a longer incubation period at 37°C and a 10-fold lower starting concentration of cAMP (0.1 μM). This confirmed that PDEs 1, 7 and 9 are all capable of hydrolysing both cAMP and cGMP (S7B Fig). However, under these conditions all PDE reactions reached the maximum signal at the end of the assay, whereby 100% of the cyclic nucleotide in the reactions was hydrolysed. This suggests that the experiments were performed using non-saturating conditions of cNMP, which may mask the primary hydrolysing activity of the PDEs. To test this, we performed a timecourse assay on PDE1 using our original conditions (incubating at room temperature with either 1 μM cAMP or 10 μM cGMP), and show that PDE1 is able to hydrolyse cGMP at a much faster rate than cAMP, with hydrolysis of cAMP detected only after 4 hours of incubation (S7C Fig). Therefore, despite displaying dual hydrolytic activity, PDEs 1, 7 and 9 appear to predominantly hydrolyse cGMP.

To test if BIPPO directly acts on one or more of the tagged PDEs, which may explain its role in triggering egress, we treated each of the immunoprecipitated PDEs with BIPPO and tested their activity. We found that of the 3 predominantly cGMP-specific PDEs, PDE1 and 7 were significantly inhibited by BIPPO, while PDE9 appeared less sensitive to BIPPO treatment. Interestingly the cAMP-specific PDE2 showed no statistically significant change in activity upon treatment with BIPPO (Fig 5F). When we performed the BIPPO inhibition assays using the conditions used by Moss *et al*., we found that the cGMP hydrolytic activity of PDE1 was completely inhibited (S7B Fig), similar to our findings in Fig 5F. However, PDEs 7 and 9, which previously showed significant, but incomplete inhibition under our more stringent conditions, were no longer inhibited. Collectively these data show that BIPPO inhibits the cGMP-specific hydrolytic activity of *Toxoplasma* PDEs more potently than their cAMP-specific hydrolytic activity and lends further support that PDE2 is a cAMP-specific PDE. Interestingly, we found that both the cAMP- and cGMP-hydrolysis activities of PfPDEβ are inhibited with BIPPO. It will be interesting in the future to evaluate the structural differences between the PDEs and the inhibitory potential of BIPPO.

### PDEs 1 & 2 are important but not essential during the lytic cycle, with PDE2 contributing to the A23187-mediated feedback loop in a CDPK3-independent manner

**Fig 6:**
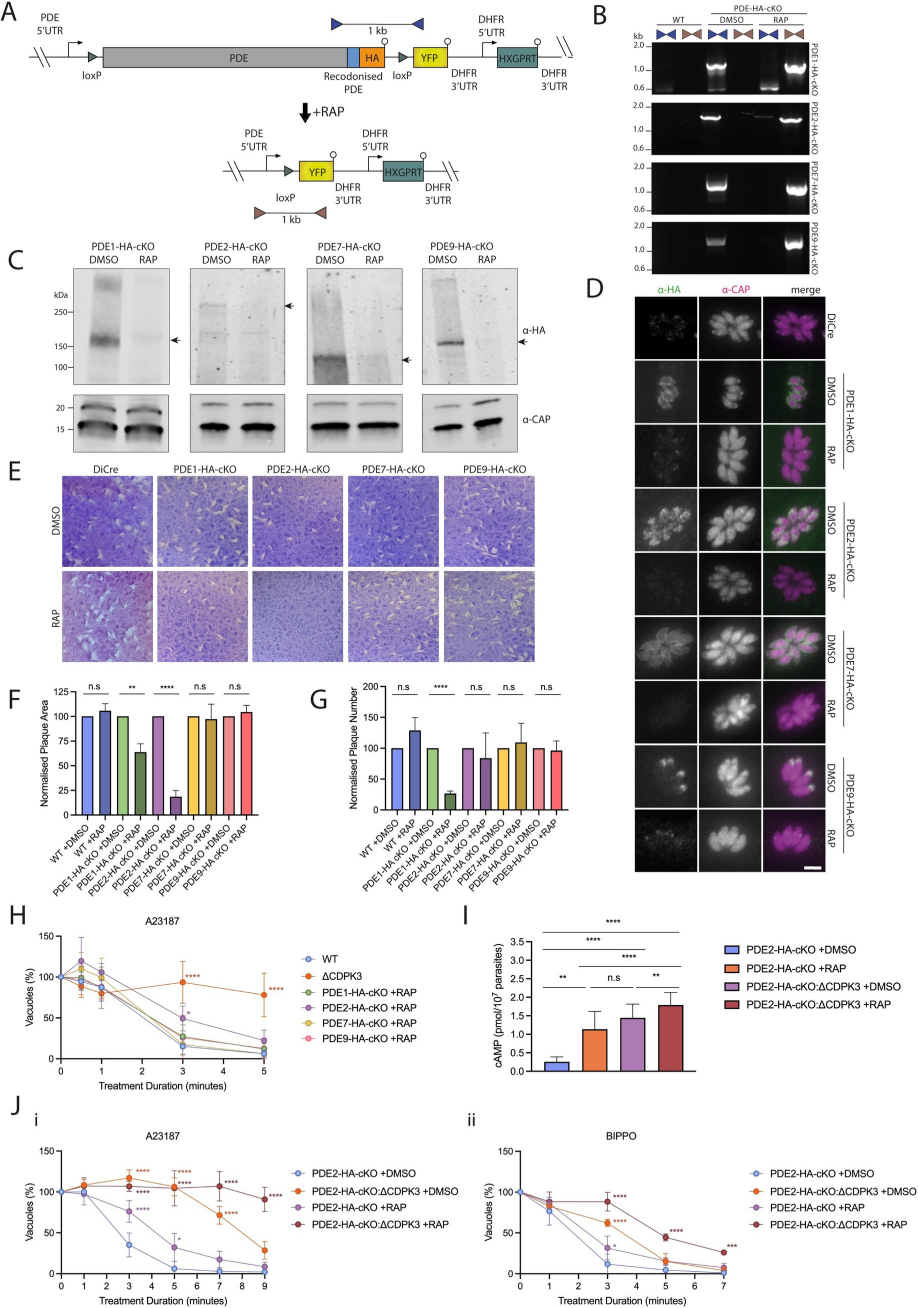
**PDE1 and PDE2 are important for lytic growth, with ΔPDE2 parasites displaying an A23187-mediated egress defect similar to ΔCDPK3 parasites (A)** Schematic representation of PDE2 rapamycin mediated deletion of PDE cKO lines. Addition of rapamycin leads to excision of the entire gene, placing YFP under the control of the PDE promoter. Coloured triangles represent primer pairs used to detect unexcised (blue) and excised (brown) loci. **(B)** PCR analysis of DMSO- and RAP-treated PDE cKO parasite lines showing near complete excision for all lines. **(C)** Western blot analysis of PDE cKO lines showing near complete loss of the PDEs at the protein level following treatment with RAP. PDEs at their expected molecular weights as depicted by arrows **(E)** Representative images of plaque assays performed on DMSO- and RAP-treated WT and PDE cKO lines after a period of 5 days. Assays were performed in biological triplicates. **(F)** Measurement of plaque area shown in Fig 6E. Data are represented as mean ± s.d. (n = 3). Significance was assessed using multiple t-tests. ****, *P* ≤ 0.0001; **, *P* ≤ 0.01; n.s, not significant. **(G)** Quantification of plaque numbers shown in Fig 6E. Data are represented as mean ± s.d. (n = 3). Significance was assessed using multiple t-tests. ****, *P* ≤ 0.0001; n.s. not significant. **(H)** Egress assay of GFP-T2A-jRCaMP1b expressing WT, ΔCDPK3 and RAP-treated PDE-HA-cKO parasites following treatment with 8 μM A23187. Data are represented as mean ± s.d. (n = 3). Two-way ANOVA.****, *P* ≤ 0.0001; *, *P* ≤ 0.05. **(I)** Quantification of intracellular cAMP levels of PDE2-HA-cKO and PDE2-HA-cKO:ΔCDPK3 parasites treated with either DMSO or RAP. Data are represented as mean ± s.d. (n = 7). Two-way ANOVA with Turkey’s multiple comparisons. **, *P* ≤ 0.01; *, *P* ≤ 0.05. **(J)** Egress assay of DMSO- or RAP-treated GFP-T2A-jRCaMP1b expressing PDE2-HA-cKO and PDE2-HA-cKO:ΔCDPK3 parasites following treatment with **(i)** 8 μM A23187 or **(ii)** 50 μM BIPPO. Data are represented as mean ± s.d. (n = 3). Two-way ANOVA. ****, *P* ≤ 0.0001; ***, *P* ≤ 0.001; *, *P* ≤ 0.05.

We next wanted to establish which of the aforementioned PDEs were essential for lytic growth. Addition of rapamycin (RAP) to the HA-tagged PDE cKO lines leads to excision of the PDE gene of interest in the respective cKO lines (Fig 6A). Despite near complete excision in all lines as observed by PCR 24 hours post RAP treatment (Fig 6B), it was only until 3 days post-treatment that we saw complete protein depletion below detectable levels (Fig 6C and 6D). Therefore, all subsequent experiments were conducted with parasites 3 days post RAP treatment. To assess the impact of PDE disruption on parasite viability and growth, we performed plaque assays using DMSO- and RAP-treated PDE cKO parasites and measured the size and number of plaques after 5 days (Fig 6E–6G). All knockout lines were able to form plaques, with deletion of PDE7 and PDE9 resulting in no significant changes in plaque size or number. PDE1 and PDE2 knockout lines, by contrast, formed much smaller plaques, with a 37% and 81% reduction in plaque sizes respectively. Despite a marked reduction in plaque size for PDE2 knockout parasites, there was no significant change in the number of plaques compared to the DMSO-treated line. Deletion of PDE1 on the other hand resulted in a 4-fold reduction in the number of plaques formed. Overall, these results suggest that both PDE1 & PDE2 are important but not essential for lytic growth and that PDE1 may be important for egress and/or invasion due to the reduced number of plaques formed following its disruption. Our observed phenotypes for PDE 1, 7 and 9 KOs are in agreement with a recent study which knocked down all predicted *Toxoplasma* PDEs [17]. While in our study deletion of PDE2 led to a reduction in plaque area, Moss *et al.* observed a defect in both plaque area and overall plaque number. This disparity is most likely explained by the different systems used to deplete PDE2 levels.

To determine whether disruption of any of the PDEs would lead to an A23187-mediated egress delay, similar to ΔCDPK3 parasites, we performed medium-throughput plate-based egress assays. We reasoned that if one of the PDEs is involved in the A23187-mediated feedback loop, disruption of this PDE would mimic, at least partially, the A23187-mediated egress delay observed in ΔCDPK3 parasites. Of the 4 PDEs, only deletion of PDE2 led to a modest egress defect (Figs 6H and S7), suggesting an important, but non-essential role for PDE2 in A23187-induced egress. To test whether the role of PDE2 is solely dependent on CDPK3, or plays an independent role, we deleted CDPK3 in the PDE2-HA-cKO line (PDE2-HA-cKO:ΔCDPK3). Upon deletion of both CDPK3 and PDE2, we observed a significant increase in cAMP levels compared to either single gene deletion (Fig 6I). To obtain experimentally independent support for elevated cAMP levels, we compared the single and double knockout parasites in egress assays. The double gene deletion exacerbated the A23187-mediated egress defect, supporting the biological relevance of the observed elevated cAMP levels (Fig 6Ji). BIPPO treatment partially rescued this egress inhibition (Fig 6Jii). Collectively these data show that while both CDPK3 and PDE2 are part of the feedback loop controlling induced egress, PDE2 activity is likely not directly dependent on CDPK3 function, and other cAMP metabolising enzymes are contributing to the elevated cAMP levels in ΔCDPK3 parasites. The data also suggests that BIPPO-mediated signalling can still overcome the combined effect of PDE2 and CDPK3 on cAMP levels, however the egress defect of the double knockout is more pronounced than either gene deletion on its own.

## Discussion

In this study we have aimed to unravel the complexity of the signalling pathways that govern the control of host cell egress of *Toxoplasma* from its host cell. Several signalling components conserved in higher eukaryotes have previously been identified, and their connectivity, to some extent described. However, published data is not currently supported by a model that fits all experimental results; there is, at present, no model that accounts for the fact that PKG pathway activation only partially rescues the ionophore-driven egress delay observed in ΔCDPK3 parasites. Our data, while in keeping with existing literature that highlights the importance of cNMP, Ca^2+^ and lipid signalling on parasite exit from the host cell, adds an additional key layer of information: Ca^2+^ release drives a signalling loop that, in part through CDPK3 activation, facilitates rapid parasite egress. This enhanced understanding of how the signalling pathways are interconnected is essential for both our understanding of the regulation of host cell egress, and of the integration of environmental or endogenous signals in general. This is particularly important as we do not fully understand how *Toxoplasma* parasites sense and react to their environment. Indeed, the plethora of calcium-dependent kinases and phosphodiesterases imply a highly complex and sensitive interplay of signalling pathways to finetune cellular responses to inputs. Our results are likely of importance beyond *Toxoplasma gondii* research: the plant-like calcium-dependent kinases (CDPKs) are conserved in *Plasmodium* species where it has been shown that PDE inhibitors can overcome disruption of PfCDPKs [29]. Therefore, the conservation of CDPKs across the apicomplexans could signify a conservation of this feedback loop and its relevance in other parasite species.

The feedback loop we describe does not control release of Ca^2+^ from internal stores, since Stewart *et al*. have shown that disruption of CDPK3 does not lead to a delay in Ca^2+^ release [26]. More likely, and in keeping with our cAMP measurements, CDPK3 directly or indirectly downregulates levels of cAMP. This, in turn, alters the activity of the cAMP-dependent protein kinase, PKAc. While PKAc inactivation is important for egress to occur, PKG needs to be activated. How PKG is activated under these conditions requires further investigation; while we have been able to measure an increase in cGMP levels upon BIPPO treatment, likely through inactivation of cGMP-specific PDEs or activation of the GC, we did not observe an increase of cGMP levels upon A23187 treatment. Similarly, Jia and colleagues found a clear dependency on PKG for parasites to egress upon PKAc depletion, but they were equally unable to reliably ascertain cGMP accumulation in intracellular parasites [7]. While it is possible that our collective inability to observe altered cGMP levels following A23187 treatment or CDPK3 depletion is explained by the sensitivity limits of the assay employed, it is equally possible that local nuanced changes in cGMP levels cannot be readily detected when measuring global cGMP levels. Indeed, the preponderance of A23187-dependent phosphorylation of cGMP hydrolysing PDEs, the GC, and UGO (a cofactor required for GC activity) [5], does suggest that A23187-treatment leads to some modulation or fine-tuning of the cGMP signalling pathway. It is, of course, also possible that cAMP-mediated signalling is exerting its effects on the PKG signalling pathway in a cGMP-independent manner. While no such mechanism has been described, it is possible that phosphorylation of PKG may lead to changes in its affinity for cGMP or that it may regulate the activity of the kinase itself.

We also identified dysregulation of DAG and phospholipid signalling in ΔCDPK3 parasites following A23187 treatment. These data provide an explanation as to why ΔCDPK3 parasites display a defect in microneme secretion [21,25], which is expected if the dysregulation of DAG leads to reduced PA levels [8]. We also demonstrate that the A23187-mediated feedback loop leads to changes in cAMP levels following A23187 treatment, which we show here occurs in a CDPK3- and PDE2-dependent manner. We identify PDE2 as one contributor of cAMP control, and establish that its deletion leads to an egress phenotype following A23187 treatment. While this finding clearly implicates PDE2 in the Ca^2+^-driven feedback loop, we were unable to detect a direct link between PDE2 and CDPK3, since none of the phosphorylation sites we detected on PDE2 are CDPK3-dependent. Although it is possible that CDPK3-dependent phosphorylation sites on PDE2 were not detected in our mass-spectrometry experiments for technical reasons, or that PDE2 is indirectly regulated by CDPK3, the double PDE2/CDPK3 gene deletions, and modest egress phenotype of ΔPDE2 (relative to ΔCDPK3), suggests that additional cAMP signalling modulators are also at play. These could be as yet unidentified PDEs with cAMP specificity, the combined effect of multiple PDEs, or an adenylyl cyclase (AC). As mentioned earlier, however, a previous study has shown that deletion of ACβ alone does not lead to a loss in parasite fitness, and is therefore not required for egress or invasion [7]. As such, it is likely that a more complex regulatory mechanism is at play. Regardless of the molecular basis for the cAMP dysregulation, our data supports a Ca^2+^-driven feedback loop, involving both CDPK3 and PDE2, that exerts its effects on egress by regulating cAMP signalling.

As the research community continues to identify novel egress signalling components, it will become increasingly clear precisely how these signalling components are interconnected [48]. For example, a recent report has identified SPARK, a novel kinase that appears to mediate Ca^2+^ release in a PKG-dependent manner and can be largely bypassed via treatment with A23187 [49]. While A23187 treatment appears to restore absolute levels of Ca^2+^ release in SPARK-depleted parasites, the rate of both calcium release and egress remains partially delayed. These findings suggest that PKG-regulated SPARK still contributes, to some degree, to A23187-mediated egress. This observation is in keeping with our proposition that A23187 signalling feeds back into the PKG signalling pathway in either a direct or indirect manner.

While we only compared BIPPO/A23187-driven signalling pathways at peak calcium flux, our timecourse data using A23187 identifies rapid CDPK3-dependent differences on proteins involved in cyclic nucleotide and phospholipid signalling as early as 15 seconds post-induction. This suggests that the feedback loop identified/described in this paper plays a role at the very onset of the signalling cascades, well before calcium release peaks. While we show that CDPK3 plays an important role in this feedback loop, it is almost certain that other kinases, enzymes and proteins that control second messenger signalling are involved in the finetuning and integration of the signalling pathways.

## Materials and methods

### Parasite culture and transfection

*T*. *gondii* tachyzoite RH strains lacking KU80 (*Δku80*) and HXGPRT (Δ*hxgprt*) [50,51] were cultured in a confluent monolayer of human foreskin fibroblasts (HFFs) maintained in Dulbecco’s Modified Eagle medium GlutaMAX (DMEM+ GlutaMAX, Gibco) supplemented with 10% foetal bovine serum (FBS), at 37°C and 5% CO2.

### Plasmid and parasite strain generation

Primers used throughout this study are listed in S6 Table. The calcium sensor construct was generated as recently described [30]. The construct was linearised using NaeI and transfected into RH *Δku80Δhxgprt* parasites as described previously [52] to generate the GFP-T2A-jRCaMP1b calcium sensor line. Transgenic parasites were subjected to 5’-fluo-2’-deoxyuridine (FUDR) selection (5 μM) 24 hrs after transfection. To generate the GFP-T2A-jRCaMP1b ΔCDPK3 line, the *HXGPRT* casette (flanked by 5’ and 3’ DHFR UTR sequences) was PCR amplified from *pGRA*-HA_HXGPRT [53] using primers 1/2 (introducing 40bp CDPK3 homology regions to the amplified fragment) and co-transfected into RH *Δku80Δhxgprt* with pSag1::Cas9-U6::dbl-sgCDPK3. The pSag1::Cas9-U6::dbl-sgCDPK3 vector was generated by inverse PCR amplification of the pSag1::Cas9-U6 [54] vector using primer pairs 3/4 and 3/5 to generate intermediate constructs pSag1::Cas9-U6::sg1CDPK3 (comprising sgRNA1) and pSag1::Cas9-U6::sg2CDPK3 (comprising sgRNA2) respectively. Following circularisation of both intermediate constructs using KLD reaction buffer (NEB), a region comprising sgRNA1 was PCR amplified with primers 6 and 7 from pSag1::Cas9-U6::sg1CDPK3 and Gibson assembled into Kpn1/XhoI linearised pSag1::Cas9-U6:: sg2CDPK3 to generate the double sgRNA plasmid pSag1::Cas9-U6::dbl-sgCDPK3. Recombinant parasites were selected 24 hrs post transfection by addition of mycophenolic acid (MPA; 25μg/mL) and xanthine (XAN; 50 μg/mL) to culture medium. Integration of the HXGPRT cassette at the CDPK3 locus was confirmed using primer pairs 8/9 and 10/11 to confirm 5’ and 3’ integration respectively. Absence of the endogenous CDPK3 locus was confirmed using primers 12/13.

To generate the PDE1, PDE2, PDE7 and PDE9 HA-tagged conditional knockout lines, two separate repair templates were generated for each gene; one which would integrate a loxP site 100 bp upstream of the start codon, and one that would introduce a C-terminal HA epitope tag along with a second loxP site and an HXGPRT cassette downstream of the gene.

To generate the pUC19_PDE1_5’loxP repair construct, a 1 kb 5’ homology region and a 1 kb 3’ homology region were PCR amplified from genomic DNA using primers 14/15 and 16/17 respectively, with the primers designed to introduce a loxP site between the 5’ and 3’ homology regions. The fragments were then Gibson cloned into the BamHI and EcoRI sites of the pUC19 vector. To generate the pG140_PDE1-HA_3’loxP_HXGPRT plasmid, a 1 kb 5’ homology region was amplified from genomic DNA using primers 18/19 and the HA tag was amplified from a plasmid containing the sequence encoding the HA tag [55] using primers 20/21. These fragments were Gibson cloned into the HindIII & PacI sites of the pG140 plasmid to generate an intermediate plasmid. A 1 kb 3’ homology region was PCR amplified from genomic DNA using primers 22/23, while an mCherry coding sequence flanked by Gra gene UTRs was amplified from pTKO2C [56] using primers 24/25. These fragments were subsequently Gibson cloned into the SacI sites of the intermediate plasmid to generate pG140_PDE1-HA_3’loxP_HXGPRT.

The pSag1::Cas9-U6::dbl-sgPDE1 vector was generated by inverse PCR amplification of the pSag1::Cas9-U6 [54] vector using primer pairs 3/26 and 3/27 to generate intermediate constructs pSag1::Cas9-U6::sg1PDE1 (comprising sgRNA1) and pSag1::Cas9-U6::sg2PDE1 (comprising sgRNA2) respectively. Following circularization of both intermediate constructs using KLD reaction buffer (NEB), a region comprising sgRNA1 was PCR amplified with primers 6 and 7 from pSag1::Cas9-U6::sg1PDE1 and Gibson assembled into Kpn1/XhoI linearised pSag1::Cas9-U6:: sg2PDE1 to generate the double sgRNA plasmid pSag1::Cas9-U6::dbl-sgPDE1.

After linearising pUC19_PDE1_5’loxP with HindIII & EcoRI and pG140_PDE1-HA_3’loxP_HXGPRT with HindIII & SapI, the two repair templates were co-transfected with pSag1::Cas9-U6::dbl-sgPDE1 into the RH DiCre *Δku80Δhxgprt* line [57]. Recombinant parasites were selected 24 hrs post transfection by addition of mycophenolic acid (MPA; 25μg/mL) and xanthine (XAN; 50 μg/mL) to culture medium.

The same cloning strategy was used for all other PDE cKO lines with the primer pairs used in each step listed in S1 Table.

To generate the PDE2-HA-cKOΔCDPK3 line, the *DHFR-TS* cassette (flanked by GRA1 5’ and GRA2 3’ UTR sequences amplified from the calcium sensor construct) was PCR amplified from an the pU6-SAG1-DHFR plasmid [58] using primers 28/29 (introducing 40bp CDPK3 homology regions to the amplified fragment) and co-transfected into RH *Δku80Δhxgprt* with pSag1::Cas9-U6::dbl-sgCDPK3. Recombinant parasites were selected 24 hrs post transfection by addition of pyrimethamine (1 μM) to culture medium.

### Egress assay

Fresh tachyzoites were harvested and seeded onto confluent HFF monolayers in black 96-well imaging μ-plates (Ibidi) at an MOI of 0.5. After 28 hours of growth, egress assays were performed in triplicate at 37°C in Ringer’s buffer (155 mM NaCl, 3 mM KCl, 2 mM CaCl2, 1 mM MgCl2, 3 mM NaH2PO4, 10 mM HEPES, 10 mM glucose). The parasites were incubated with 8 μM Ca2+ ionophore A23187 (BioVision) or 50 μM BIPPO (generated in-house) for variable timings. Wells were subsequently fixed by adding 16% FA to a final concentration of 3% for 15 mins. Wells were washed with PBS and stained with 5 μg/ml DAPI. Automated image acquisition of 25 fields per well was performed on a Cellomics 561 Array Scan VTI HCS reader (Thermo Scientific) using a 20× objective. Image analysis was performed using the Compartmental Analysis BioApplication on HCS Studio (Thermo Scientific).

### Live imaging of calcium sensor line

Fresh tachyzoites were harvested and seeded (at an MOI of 0.5) onto confluent HFF cells grown on IBIDI tissue culture treated 8 well chamber slides and allowed to grow for 28 hrs in DMEM + 10%FBS. Prior to imaging, wells were washed once with PBS, and supplemented with 100 μl Ringer’s buffer. Wells were treated for 5 mins at 37°C with 100 μl 2 μg/ml Cytochalasin D in Ringer’s buffer (final concentration 1 μg/ml) to prevent egress. Imaging was performed on the Nikon Eclipse Ti-U inverted fluorescent microscope, 60x/1.4 NA Oil immersion objective, in an environmental chamber (OKOLAB) with temperature maintained at 37°C. Image capture was managed by Nikon NIS-Elements software with acquisition 1/s for 70s. At 15s following image acquisition, 100μl of A23187 (24μM) or BIPPO (150 μM) in Ringer’s buffer was added by pipette (to final concentrations of 8 μM and 50 μM respectively). ≥10 vacuoles across ≥10 wells were imaged across ≥ 7 days for each condition. Image analysis was performed using Nikon NIS-Elements analysis software. jRCaMP1b and GFP signals at 0s were set to 0 (zero) and 1 respectively. jRCaMP1b/GFP was used as a readout for ΔCa^2+^.

## Phosphoproteome analysis

### Lysis and protein digestion

Parasites were seeded onto HFF monolayers in 15cm culture dishes at an MOI of 5. 24 hours post-inoculation, plates were washed once with PBS and treated with 50 μM BIPPO (15s) or 8 μM A23187 (variable timings depending on experiment) in Ringer’s buffer. Following the appropriate treatment duration, treatments were rapidly removed and plates placed on a supercooled salt water ice bath to inhibit further signalling. Lysis was performed by scraping cells in ice cold 8 M urea, 75 mM NaCl, 50 mM Tris, pH 8.2, supplemented with protease (complete mini, Roche) and phosphatase (PhosSTOP, Roche) inhibitors. Lysis was followed by sonication to reduce sample viscosity (30% duty cycle, 3 x 30 seconds bursts, on ice). Protein concentration was measured using a BCA protein assay kit (Pierce). Lysates (1mg each) were subsequently reduced with 5 mM DTT for 30 minutes at 56°C and alkylated in the dark with 14 mM iodoacetamide for 30 minutes at RT. Following iodoacetamide quenching with 5 mM DTT for 15 minutes in the dark, lysates were diluted with 50 mM ammonium bicarbonate to < 4M urea, and digested with LysC (Promega) for 2–3 hours at 37°C. Lysates were further diluted with 50 mM ammonium bicarbonate to < 2M urea and digested with trypsin (Promega) overnight at 37°C. After digestion, samples were acidified with trifluoroacetic acid (TFA) (Thermo Fisher Scientific) to a final concentration of 1% (v/v). All insoluble material was removed by centrifugation and the supernatant was desalted on Sep-Pak C_18_ cartridges (Waters).

#### TMT labelling

Samples were dissolved at 1 mg/ml in 50 mM Na-Hepes, pH 8.5 and 30% acetonitrile (v/v) and labelled with respective TMT reagents (Thermo Fisher Scientific, 2.4 mg reagent/1 mg sample) for 1 hour at RT. Labelling was then quenched with 0.3% hydroxylamine for 15 minutes at RT and samples acidified (pH~2) with formic acid. After verification of labelling efficiency via mass spectrometry, the lysates were mixed in a 1:1 ratio, vacuum dried and desalted on Sep-Pak C_18_ cartridges.

#### Phosphopeptide enrichment

Desalted and vacuum dried samples were solubilised in 1 ml of loading buffer (80% acetonitrile, 5% TFA, 1 M glycolic acid) and mixed with 5 mg of TiO_2_ beads (Titansphere, 5 μm GL Sciences Japan). Samples were incubated for 10 minutes with agitation, followed by a 1 minute 2000 × g spin to pellet the beads. The supernatant was removed and used for a second round of enrichment as explained below. Beads were washed with 150 μl loading buffer followed by two additional washes, the first with 150 μl 80% acetonitrile, 1% TFA and the second with 150 μl 10% acetonitrile, 0.2% TFA. After each wash, beads were pelleted by centrifugation (1 minute at 2000 × g) and the supernatant discarded. Beads were dried in a vacuum centrifuge for 30 minutes followed by two elution steps at high pH. For the first elution step, beads were mixed with 100 μl of 1% ammonium hydroxide (v/v) and for the second elution step with 100 μl of 5% ammonium hydroxide (v/v). Each time beads were incubated for 10 minutes with agitation and pelleted at 2000 × g for 1 minute. The two elutions were removed following each spin, and subsequently pooled together before undergoing vacuum drying. The supernatant from the TiO_2_ enrichment was desalted on Sep-Pak C_18_ and the High Select Fe-NTA phosphopeptide enrichment kit (Thermo Fisher Scientific) was used according to manufacturer’s instructions for a second round of enrichment.

#### Sample fractionation and desalting

Combined TiO_2_ and Fe-NTA phosphopeptide eluates were fractionated using the Pierce High pH Reversed-Phase kit (Thermo Fisher Scientific) according to manufacturer’s instructions. Resulting fractions were taken to dryness by vacuum centrifugation and further desalted on a stage tip using Empore C18 discs (3M). Briefly, each stage tip was packed with one C18 disc, conditioned with 100 μl of 100% methanol, followed by 200 μl of 1% TFA. The sample was loaded in 100 μl of 1% TFA, washed 3 times with 200 μl of 1% TFA and eluted with 50 μl of 50% acetonitrile, 5% TFA. The desalted peptides were vacuum dried in preparation for LC-MS/MS analysis.

#### LC-MS/MS

Samples were resuspended in 0.1% TFA and loaded on a 50 cm Easy Spray PepMap column (75 μm inner diameter, 2 μm particle size, Thermo Fisher Scientific) equipped with an integrated electrospray emitter. Reverse phase chromatography was performed using the RSLC nano U3000 (Thermo Fisher Scientific) with a binary buffer system (solvent A: 0.1% formic acid, 5% DMSO; solvent B: 80% acetonitrile, 0.1% formic acid, 5% DMSO) at a flow rate of 250 nl/minute. The samples were run on a linear gradient of 5–60% B in 150 minutes with a total run time of 180 minutes including column conditioning. The nanoLC was coupled to an Orbitrap Fusion Lumos mass spectrometer using an EasySpray nano source (Thermo Fisher Scientific). The Orbitrap Fusion Lumos was operated in data-dependent mode using two acquisition methods. For the MS2 method, HCD MS/MS scans (R = 50,000) were acquired after an MS1 survey scan (R = 120, 000) using MS1 target of 4E5 ions, and MS2 target of 2E5 ions. The number of precursor ions selected for fragmentation was determined by the “Top Speed” acquisition algorithm with a cycle time of 3 seconds, and a dynamic exclusion of 60 seconds. The maximum ion injection time utilised for MS2 scans was 86 ms and the HCD collision energy was set at 38. For the MS3 method, CID MS/MS scans (R = 30,000) were acquired after an MS1 survey scan with parameters as above. The MS2 ion target was set at 5E4 with multistage activation of the neutral loss (H3PO4) enabled. The maximum ion injection time utilised for MS2 scans was 80 ms and the CID collision energy was set at 35. HCD MS3 scan (R = 60,000) was performed with synchronous precursor selection enabled to include up to 5 MS2 fragment ions. The ion target was 1E5, maximum ion injection time was 105 ms and the HCD collision energy was set at 65. Acquired raw data files were processed with MaxQuant [59,60] (version 1.5.2.8) and peptides were identified from the MS/MS spectra searched against *Toxoplasma gondii* (combined TG1, ME48 and VEG proteomes, ToxoDB release 27 & 37) and *Homo sapiens* (UniProt, 2016 and 2018) proteomes using Andromeda [60] search engine. TMT based experiments in MaxQuant were performed using the ‘reporter ion MS2 or MS3’ built-in quantification algorithm with reporter mass tolerance set to 0.003 Da. Cysteine carbamidomethylation was selected as a fixed modification. Methionine oxidation, acetylation of protein N-terminus, deamidation (NQ) and phosphorylation (S, T, Y) were selected as variable modifications. The enzyme specificity was set to trypsin with a maximum of 2 missed cleavages. The precursor mass tolerance was set to 20 ppm for the first search (used for mass re-calibration) and to 4.5 ppm for the main search. The datasets were filtered on posterior error probability to achieve a 1% false discovery rate on protein, peptide and site level. ‘Match between runs’ option was enabled for fractionated samples (time window 0.7 min) and “Unique and razor peptides” mode was selected to allow identification and quantification of proteins in groups (razor peptides are uniquely assigned to protein groups and not to individual proteins). All mass spectrometry acquisition files and MaxQuant processing files have been deposited to the ProteomeXchange Consortium via the PRIDE [61] partner repository (PXD030057).

### Phosphoproteome data processing

#### A23187/BIPPO analysis (set1 and set2)

The data were analysed using Perseus [62] (version 1.5.0.9) and Microsoft Office Excel 2016. Briefly, the data were filtered to remove common contaminants, IDs originating from reverse decoy sequences and sites originating from the host (human) proteome. Individual TMT reporter intensities (MS2-based acquisition) and total intensity were log2 and log10 transformed, respectively. Log2 reporter intensities for each sample were subsequently normalised (centred) by subtracting the median log2 reporter intensity value calculated for all non-phosphorylated peptides detected in the same sample. Data were then filtered by 1 valid value to retain only the quantified phosphosites and log2 fold changes in reporter intensity between conditions were calculated. Differentially regulated (DR) phosphorylation sites were identified by calculating the median absolute deviation (MAD) for the log2FC in each comparative dataset. The largest of these was used to set an outlier threshold of 3x MAD (rounded to the nearest tenth; log2FC>0.5 for up-regulated sites and log2FC<-0.5 for down-regulated sites) and applied across all datasets.

#### A23187 timecourse analysis

The data were analysed using Perseus [62] (version 1.5.0.9) and Microsoft Office Excel 2016. TMT reporter intensities obtained via MS2 and MS3-based acquisition were filtered to remove common contaminants, IDs originating from reverse decoy sequences and sites originating from the host (human) proteome. MS2/MS3 reporter intensities and the total intensity were then log2 and log10 transformed, respectively. Log2 reporter intensities for each sample were subsequently normalised (centred) by subtracting the median log2 reporter intensity value calculated for all non-phosphorylated peptides detected in the same sample. Data were then filtered by 1 valid value to retain only the quantified phosphosites. Finally, log2 fold changes were calculated relative to a 0s (DMSO) control, separately for the MS2 and MS3 data, to obtain per-site response to ionophore treatment. For downstream analysis responses obtained by the MS2 and MS3 based quantification were averaged.

DR thresholds were determined in a timepoint-specific manner by calculating the log2FC MADs scores across each WT timepoint (15s, 30s and 60s), and setting 3x MAD outlier thresholds for each (rounded to the nearest tenth: 15s log2FC<-0.5 for DR^DOWN^ and 15s log2FC>0.5 for DR^UP^; 30s log2FC<-0.6 for DR^DOWN^ and 30s log2FC>0.6 for DR^UP^; 60s log2FC<-0.9 for DR^DOWN^ and 60s log2FC>0.9 for DR^UP^). Phosphorylation sites were considered to be differentially regulated if at any given timepoint their log2FC surpassed these thresholds.

CDPK3 dependency was determined for each phosphorylation site by calculating the log2 ratios of A23187-treated WT and ΔCDPK3 parasites (ΔCDPK3^A23187^/WT ^A23187^) for each timepoint. The resulting ratios were used to calculate the MAD at each timepoint, and the most stringent score was used to set 3X MAD outlier thresholds (rounded to the nearest tenth: log2FC<-0.6 for CDPK3 dependency in DR^UP^ sites and log2FC>0.6 for CDPK3 dependency in DR^DOWN^ sites). A DR site was considered to be CDPK3-dependent if, at any given timepoint, it simultaneously passed the appropriate DR and CDPK3 dependency thresholds.

#### Clustering

Phosphosite log2FC values from the timecourse experiment were clustered using a Gaussian finite mixture model-based method [63] log2FC values from both the WT and ΔCDPK3 samples were combined, thus the clustering was performed on six dimensions: WT 15s, 30s and 60s and ΔCDPK3 15s, 30s and 60s. The method was restricted to spherical models with equal or unequal volumes (models “EII” and “VII”) and models with up to 11 clusters were tested. The clustering method was applied separately to the sites designated as up-regulated, down-regulated, and CDPK3-dependent.

#### Gene ontology enrichment

Each cluster was tested for an enrichment in Gene Ontology annotations using goatools version 0.8.12 [64]. The *T*.*gondii* proteome was used as background set. Fisher’s exact test was used to calculate enrichment, and p-values were adjusted for false discovery rate (Benjamini-Hochberg). Default settings were used. The ontology was downloaded from https://geneontology.org on 2019 April 17.

#### Motif analysis

The sequence surrounding each DR timecourse phosphosite, +/-7 residues, was subjected to a motif analysis using rmotifx 1.0 (motif enrichment) [65] and WebLogo 3.7.1 [66]. The analysis was performed for each cluster as well as for the combined sets of phosphosites designated as up-regulated, down-regulated and CDPK3-dependent.

### Measurement of cyclic nucleotide levels in extracellular parasites

Parasites were seeded onto HFF monolayers in T175 flasks. After 24–30 hours, flasks were washed once with PBS, then scraped and syringe lysed in endo buffer (44.7 mM K_2_SO_4_, 10 mM MgSO_4_, 106 mM sucrose, 5 mM glucose, 20 mM Tris–H_2_SO_4_, pH 8.2)/ After counting, the parasites were aliquoted into eppendorfs and treated with 50 μM BIPPO, 8 μM A23187 or the equivalent volume of DMSO for the variable timings while maintained at 37°C. The samples were then lysed by adding two volumes of 0.1 M HCl and left on ice for 10 minutes with intermittent vortexing. The levels of cAMP and cGMP levels in the samples was determined using the enzyme-linked immunosorbent assay (ELISA)-based high-sensitivity direct cAMP and cGMP colorimetric assay kits (Enzo Life Sciences). Samples and standards were acetylated in order to improve sensitivity. All samples and standards were set up in duplicate. Absorbance was measured at 405nm using a FLUOstar Omega plate reader. The detection ranges were 0.078 to 20 pmol/ml and 0.08 to 50 pmol/ml for the cAMP and cGMP assays, respectively.

### DAG & global lipid metabolomics

For the DAG and lipid kinetics experiments, parasites were grown for 3 days in DMEM containing 10% FBS and syringe released by passing through a 23-gauge needle. Lysed parasites were filtered through a 10 μm polycarbonate filter, counted and then pelleted (1,800 rpm, 10 min). After washing pellets with DMEM containing 10 mM HEPEs, parasites were resuspended to a concentration of 4x10^8^ cells per ml and 0.5ml was each aliquoted into 1.5ml tubes, to achieve 1x10^8^ cells per tube, and maintained at room temperature. Parasites were shifted to 37°C and allowed to equilibrate for 60 seconds before addition of 0.5 ml of pre-warmed DMEM containing either DMSO or 16 μM A23187 (8 μM final concentration). Parasites were allowed to incubate for the desired time and quenched immediately on dry ice/ethanol for 5 seconds then left on ice. Cells were pelleted (8,000 rpm, 2 min) and washed 3x with 1 ml of ice-cold PBS. Pellets were then stored at –80°C until required.

Alternatively for global lipidomics, parasites were grown for 3 days in DMEM containing 10% FBS, then scraped and rapidly quenched in a dry ice ethanol bath and placed on ice. Tachyzoites were then released by passing through a 23-gauge needle and filtered through a 10 μm polycarbonate filter, counted and pelleted (1800 rpm, 10 minutes) at 4°C. Cells were washed twice with ice cold 1x PBS and then pellets were stored at -80 until required.

### Lipid analysis

Total lipids and internal standards were extracted using chloroform:methanol, 1:2 (v/v) and chloroform:methanol, 2:1 (v/v) in the presence of 0.1 M HCl with periodic sonication. The organic phase was dried under N_2_ gas and dissolved in 1-butanol. For DAG, total lipid was then separated by 1D-HPTLC using hexane: diethyl-ether: formic Acid, 40:10:1. For global phospholipid analysis including PA, total lipid was spiked with 1 μg PA(C17:0/C17:0) (Avanti Polar lipids) and then separated by 2D-HPTLC using chloroform/methanol/28% NH_4_OH, 60:35:8 (v/v) as the 1st dimension solvent system and chloroform/acetone/methanol/acetic acid/water, 50:20:10:13:5 (v/v) as the 2nd dimension solvent system [67]. All lipid spots including PA were visualised with primulin and scraped. Lipids and additional standards were then prepared for GC-MS analysis in hexane (Agilent 5977A- 7890B) after derivatisation by methanolysis using 0.5 M HCl in methanol incubated at 85°C for 3.5 hrs. Fatty acid methyl esters were identified by their mass spectrum and retention time compared to authentic standards. Individual lipid classes were normalised according to internal standards.

### Parasite protein extraction, SDS-PAGE, and immunoblotting

Intracellular parasites were scraped and syringe released HFFs by passing through a 23-gauge needle. Extracellular parasites were pelleted (8,000 rpm, 10 min) then lysed in an NP40 buffer (150mM NaCl, 0.5mM EDTA, 1% NP-40, 10mM Tris [pH 7.5]) supplemented with cOmplete EDTA-free protease inhibitor (Roche). Samples were incubated on ice for 10 min, then centrifuged at 12,000xg for 10 min at 4°C and supernatants collected. Following the addition of SDS sample buffer, the samples were electrophoresed on 4–20% Mini-Protean TGX stain-free precast gels (Bio-Rad) then transferred onto nitrocellulose mem- branes using a semi dry Trans-Blot Turbo transfer system (Bio-Rad). The membranes were blocked using 10% skimmed milk in PBS containing 0.1% Tween 20 (PBST) and then incubated with rat anti-HA high affinity (1:1,000; Roche) and rabbit anti-T. gondii CAP (1:2,000) [57] for 1 hour, followed by donkey anti-rabbit IRDye 680LT (1:20,000; LI-COR) and goat anti-rat IRDye 800CW (1:20,000; LI-COR) for 1 hour. After several washed with PBS, the remaining bound near-infrared conjugated secondary antibodies were visualised using the Odyssey Infrared Imaging System (LI-COR Biosciences, Nebraska, United States).

### Immunofluorescence microscopy

Parasites were seeded onto HFFs grown in chambered coverslip slides (Ibidi). After 18–24 hours, the chambers were washed with PBS, then fixed in 3% formaldehyde in PBS for 15 minutes. The cells were then permeabilised using 0.1% Triton X-100 in PBS for 10 minutes, then blocked in 3% BSA in PBS for 1 hour. The samples were then incubated rat anti-HA high affinity (1:1,000; Roche) and rabbit anti-T. gondii CAP (1:2,000) [57] for 1 hour followed by goat anti-rabbit Alexa Flour 594 (1:2,000; Life Technologies) and donkey anti-rat Alexa Flour 488 (1:2,000; Life Technologies) secondary antibodies along with 5 μg/ml DAPI for 1 hour. Images were obtained using a Nikon Eclipse Ti-U inverted fluorescent microscope using a 100x objective and processed using ImageJ software.

### PDE pulldown

HFF monolayers infected with WT or HA-tagged PDE1, PDE2, PDE7 or PDE9 lines were scraped, syringe-released and counted. A total of 1x10^7^ cells per condition were pelleted (8,000 rpm, 10 min) then lysed in 10 μl of NP-40 buffer (150mM NaCl, 0.5mM EDTA, 1% NP-40, 10mM Tris [pH 7.5]) supplemented with cOmplete EDTA-free protease inhibitor (Roche) for 10 minutes on ice. Samples were centrifuged at 12,000 xg for 10 min at 4°C, the supernatants collected and the volume adjusted to 100 μl with IP buffer (150mM NaCl, 0.5mM EDTA, 10mM Tris [pH 7.5] and cOmplete EDTA-free protease inhibitor) to give a final detergent concentration of 0.1%. To pull down HA-tagged PDEs, Pierce anti-HA conjugated magnetic beads (5 μl per condition, Thermo Fisher) were washed 3x with IP buffer to equilibrate the beads, after which the diluted lysate samples was added to the beads and incubated for 2 hours at 4°C on a rotating wheel. After incubation, the supernatant was discarded and the beads washed 3x with IP buffer.

### PDE assay

The hydrolytic activity of immunoprecipitated PDEs bound to anti-HA magnetic beads was measured using the PDE-Glo assay (Promega), a bioluminsescence-based assay which quantifies the amount of cAMP or cGMP hydrolysed by a given PDE. Briefly, the PDE-bound magnetic beads were resuspended in assay buffer and incubated with either 1 μM cAMP or 10 μM cGMP for 1 hour at room temperature and the reaction terminated by the addition of termination buffer. Detection buffer was added and incubated for 20 minutes, followed by the addition of Kinase-Glo detection solution which was incubated for a further 10 minutes. The supernatants were then transferred to a white 96-well plate and luminescence measured using a FLUOstar Omega plate reader. To test the inhibitory effects of BIPPO on the activity of the PDEs, 25 μM BIPPO or the equivalent volume of DMSO was added to the reaction buffer and left to incubate for 10 minutes before addition of cyclic nucleotide.

Percentage hydrolytic activity for each sample was calculated by taking the bioluminescence value of the sample, subtracting the background, then dividing that value by the bioluminescence resulting from 100% hydrolysis (where no cyclic nucleotide was added) and multiplying by 100.

### Plaque assays

Intracellular parasites were treated with 50 nM RAP or the equivalent volume of DMSO for 4 hours, after which the media was replaced, and the parasites left to grow for 3 days to ensure efficient turnover of the PDEs in the RAP-treated samples. DMSO- and RAP-treated parasites were harvested by syringe lysis, counted and 250 parasites seeded on confluent HFFs grown in T25 flasks. Plaques were allowed to form for 5 days, after which cells were fixed in 100% ice cold methanol for 2 minutes and then stained with 0.1% crystal violet for 10 minutes to visualise the plaques. Images of the plaques were acquired with a 4x objective using an Olympus CKX53 microscope fitted with an Olympus DP74 camera. Plaque areas were determined using ImageJ software.

## Supporting information

S1 Fig Schematic representation of the key signalling components in a *Toxoplasma gondii* tachyzoite infecting a host cell. Protein localisations are based on published data, however are only representative and may not accurately represent the accurate localisation. Highlighted in red are known stimulants of egress that have been used in this study.
(JPG)

S2 Fig Egress assay of GFP-T2A-jRCaMP1b and GFP parasites following treatment with 50μM BIPPO **(A)** or 8 μM A23187 **(B)**. Graphs show the remaining % of un-egressed vacuoles (relative to untreated) following A23187/BIPPO treatment. Data are represented as mean ± s.d. (n = 3). Two-way ANOVA.
(JPG)

S3 Fig **(A)** Visual summary of the TMT-10-plex samples (sets 1 and 2) used to quantify the phosphoproteomes of intracellular WT and ΔCDPK3 tachyzoites treated with 50 μM BIPPO (15s) or 8 μM A23187 (50s). **(B)** Global phosphorylation responses (Log_2_FC frequency distribution) of WT and ΔCDPK3 tachyzoites following treatment with 8 μM A23187 (50s) or 50 μM BIPPO (15s). Dotted lines in graph represent 3xMAD outlier thresholds used to determine differential site regulation (log_2_FC>0.5 for up-regulated sites and log_2_FC<-0.5 for down-regulated sites). **(C)** Differentially regulated phosphosites were identified through the comparative analyses shown (grey circle). For these phosphosites, the reporter intensity scores for each sample replicate (grey star) were correlated, and the resulting Pearson’s correlation coefficients for each comparison are shown. R1, replicate 1; R2, replicate 2; R3, replicate 3.
(JPG)

S4 Fig **(A)** Gaussian mixture-model-based clustering of DR^DOWN^ sites in the A23187-treatment timecourses. Log_2_FC values from both WT and ΔCDPK3 samples were combined to cluster on six dimensions (WT 15s, 30s and 60s and ΔCDPK3 15s, 30s and 60s). Thin lines represent the timecourse traces of individual phosphorylation sites. Thick lines represent Loess regression fits of all traces. **(B)** Gaussian mixture-model-based clustering of DR^UP^ and **(C) DR**^**DOWN**^ CDPK3-dependent sites in the A23187-treatment timecourses. Log_2_FC values from both WT and ΔCDPK3 samples were combined to cluster on six dimensions (WT 15s, 30s and 60s and ΔCDPK3 15s, 30s and 60s). Thin lines represent the timecourse traces of individual phosphorylation sites. Thick lines represent Loess regression fits of all traces. **(D)** Results of phosphorylation motif enrichment analysis using rmotifx 1.0. Results are shown for the analysis of **(i)** DR^UP^ **(ii)** DR^UP^ CDPK3-dependent **(iii)**DR^DOWN^ and **(iv)** DR^DOWN^ CDPK3-dependent phosphorylation sites.
(JPG)

S5 Fig Pulse experiment of WT and ΔCDPK3 parasites treated with DMSO (0s) or 8 μM A23187 for 5, 10, 30, 45 or 60 seconds analysing levels of **(A)** DAG and PLs **(B)** FFAs and **(C)** TAGs, with data expressed as a ratio of ΔCDPK3/WT levels. Data are represented as mean ± s.d. (n = 4). Significance was assessed using a paired two sample t-test. **, *P* ≤ 0.01; *, *P* ≤ 0.05. **(D)** Phospholipid profile of WT and ΔCDPK3 extracellular parasites measuring the levels of lysophosphatidylinositol (LPI), lysophosphatidylcholine (LPC), phosphatidylinositol (PI), phosphatidylserine (PS), phosphatidylthreonine (PS), phosphatidic acid (PA), phosphatidylethanolamine (PE), free fatty acids (FA), sphingomyelin (SM) and phosphatidylcholine (PC). Data are represented as mean ± s.d. (n = 4). Significance was assessed using a paired t-test. n.s, not significant. **(E)** Quantification of natural egress of WT and ΔCDPK3 parasites following treatment with DMSO or 50 μM H89 (2 hrs). Graph shows the remaining % of un-egressed vacuoles (relative to untreated). Data are represented as mean ± s.d. (n = 5). Significance was assessed using an unpaired two-tailed t-test. ***, *P* ≤ 0.001; **, *P* ≤ 0.01.
(JPG)

S6 Fig Ratiometric tracking of mean Ca^2+^ response (jRCaMP1b/GFP normalised to 0) of extracellular parasites in Endo buffer following addition of **(A)** 50μM BIPPO **(B)** 8 μM A23187 or **(C)** 8 μM A23187 with 1 μM CaCl_2_. Grey dotted lines represent ± SEM. Data was collected from ≥ 30 vacuoles (in separate wells) over ≥5 days. **(D)** cGMP levels extracellular WT tachyzoites in Endo buffer supplemented with 1 μM CaCl_2_ following treatment with DMSO for 60 seconds; 50 μM BIPPO for 5 or 15 seconds; or 8 μM A23187 for 5, 15, 30 or 60 seconds. All samples were lysed in 0.1 M HCl to inactivate all PDEs and cyclases, and extracts were analysed by using a commercial ELISA-based cGMP detection assay. Data are represented as mean ± s.d. (n = 4).
(JPG)

S7 Fig **(A)** Representative immunoprecipitation of PDEs 1, 2, 7 & 9 using α-HA magnetic beads probed with α-HA antibodies showing migration of the PDEs at their expected molecular weights as depicted by arrows. **(B)** Hydrolytic activity of immunoprecipitated HA-tagged PDE1, PDE2, PDE7, PDE9 using either 100 nM cAMP or 10 μM cGMP as a substrate. Lysates from the WT parental line were also included as a control. PDEs reactions were carried out in the presence of DMSO (vehicle) or 25 μM BIPPO for 2 hours at 37°C. Data are represented as mean ± s.d. (n = 3). Significance was assessed using a paired Two-way ANOVA. ****, *P* ≤ 0.0001; **, *P* ≤ 0.01; *, *P* ≤ 0.05; n.s. not significant. **(C)** Timecourse measuring the hydrolytic activity of immunoprecipitated HA-tagged PDE1 after incubating with either 1 μM cAMP or 10 μM cGMP at room temperature for the timepoints indicated. Data are represented as mean ± s.d. (n = 2).
(JPG)

S8 Fig Egress assay of GFP-T2A-jRCaMP1b expressing WT, ΔCDPK3 and DMSO- and RAP-treated (A) PDE1, (B) PDE2, (C) PDE7 and (D) PDE9 cKO parasites following treatment with 8 μM A23187 after 0, 0.5, 1, 3, and 5 minutes. Data are represented as mean ± s.d. (n = 3). Two-way ANOVA.****, *P* ≤ 0.0001; *, *P* ≤ 0.05.
(JPG)

S1 Data Phosphopeptide quantifications and calculated logFCs from A23187/BIPPO treated WT/ΔCDPK3 parasites. Tabs include data subsets that were subjected to thresholding for differential regulation in A23187 and/or BIPPO treatment conditions. Includes data from TMT sets 1 and 2.
(XLSX)

S2 Data Phosphopeptide quantifications and calculated logFCs for peptides that were (i) differentially regulated following A21387/BIPPO treatment and (ii) were detected during CDPK3 dependency analysis. Tabs include data subsets that were subjected to thresholding for differential regulation and CDPK3 dependency. Includes data from TMT sets 1 and 2.
(XLSX)

S3 Data Phosphopeptide quantifications and calculated logFCs from WT/ΔCDPK3 parasites subjected to A23187 treatment timecourse. Tabs include data subsets subjected to thresholding for differential regulation and CDPK3 dependency.
(XLSX)

S1 Table List of proteins (identified in A23187 timecourse) assigned to clusters identified in the Gaussian mixed-model-based clustering analysis. The clusters are listed across four different tabs based on whether they are differentially up- or down-regulated and whether this shows CDPK3 dependency.
(XLS)

S2 Table Gene ontology analysis results of proteins found to be differentially up-regulated in A23187 timecourse.
(XLSX)

S3 Table Gene ontology analysis results of protein found to be differentially down-regulated in A23187 timecourse.
(XLSX)

S4 Table Gene ontology analysis results of protein found to be differentially up-regulated and CDPK3 dependent in A23187 timecourse.
(XLSX)

S5 Table Gene ontology analysis results of protein found to be differentially down-regulated and CDPK3 dependent in A23187 timecourse.
(XLSX)

S6 Table List of primers used in this study.
(XLSX)
