# Supplementary figures and images for: A positive feedback loop mediates crosstalk between calcium, cyclic nucleotide and lipid signalling in calcium-induced Toxoplasma gondii egress
Source: PLoS Pathog. 2022 Oct 20;18(10):e1010901. doi: 10.1371/journal.ppat.1010901 (PMC9624417; doi:10.1371/journal.ppat.1010901)

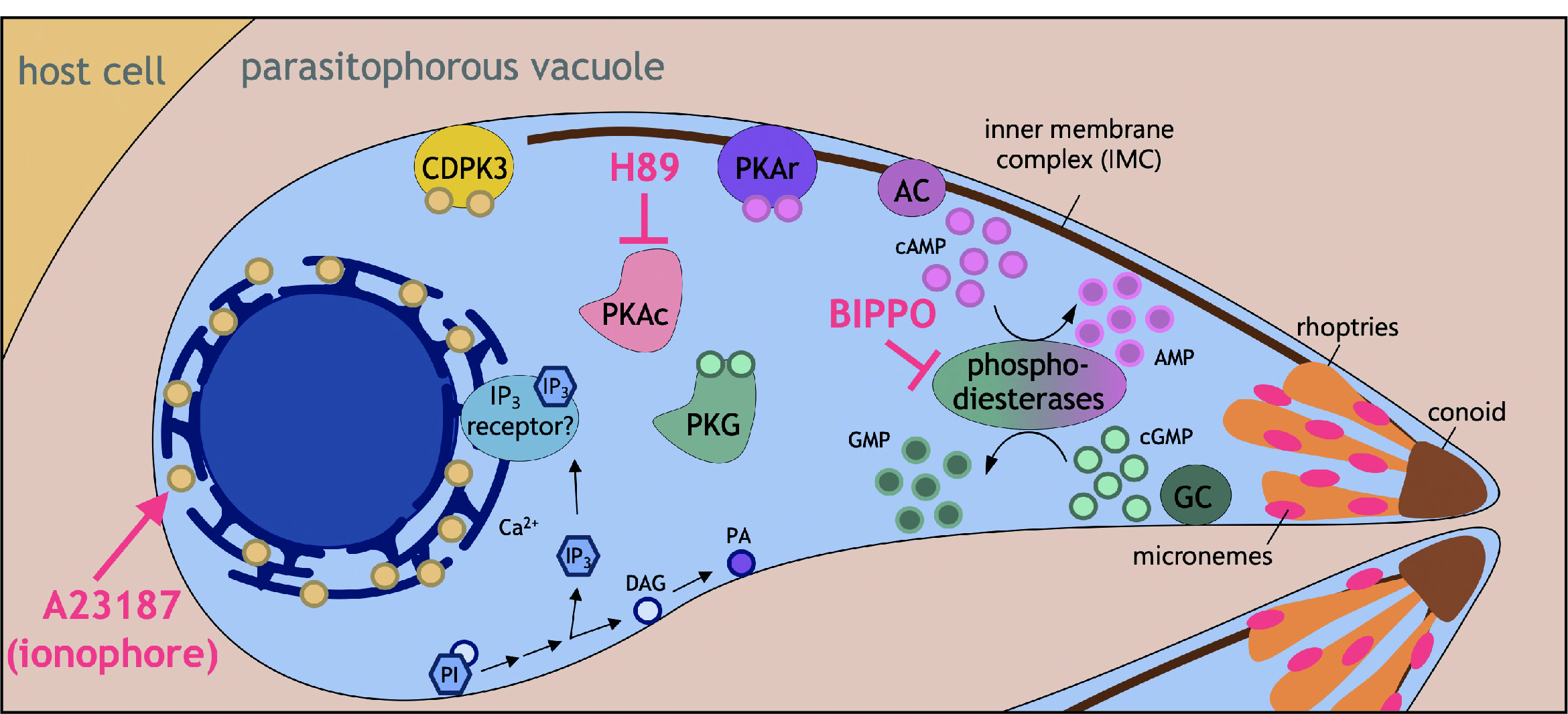

Supplement: S1 Fig — Protein localisations are based on published data, however are only representative and may not accurately represent the accurate localisation. Highlighted in red are known stimulants of egress that have been used in this study. (JPG) [file ppat.1010901.s001.jpg]

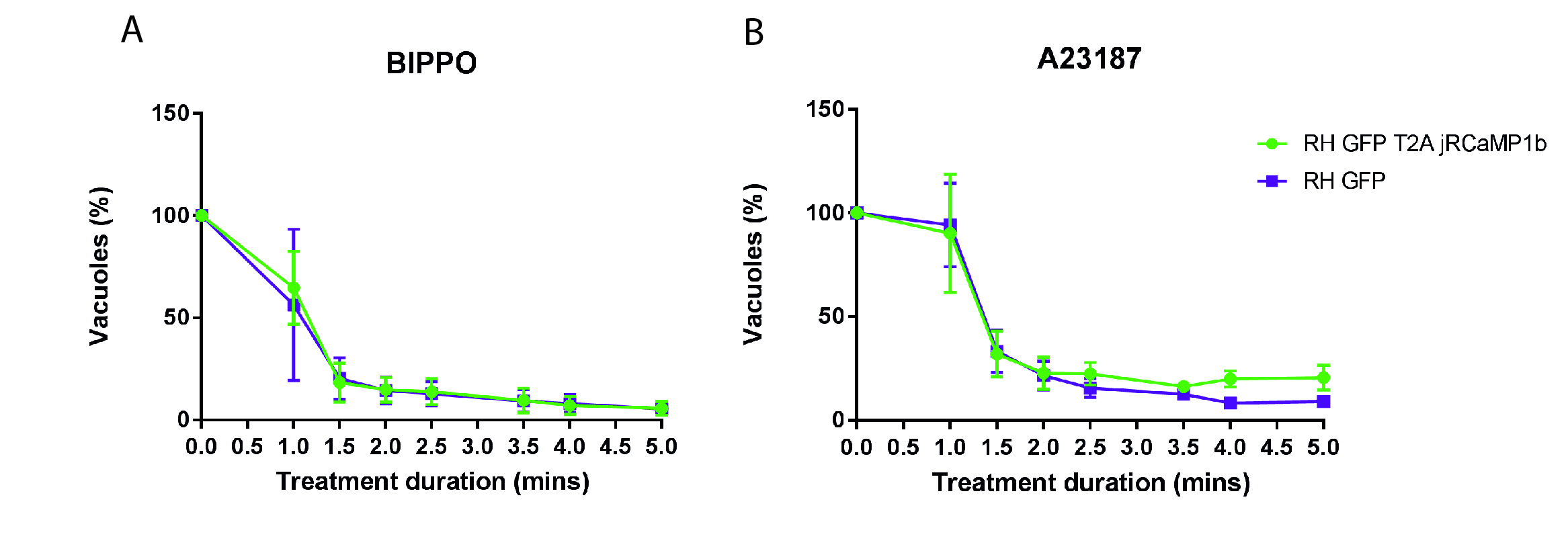

Supplement: S2 Fig — Egress assay of GFP-T2A-jRCaMP1b and GFP parasites following treatment with 50μM BIPPO (A) or 8 μM A23187 (B). Graphs show the remaining % of un-egressed vacuoles (relative to untreated) following A23187/BIPPO treatment. Data are represented as mean ± s.d. (n = 3). Two-way ANOVA. (JPG) [file ppat.1010901.s002.jpg]

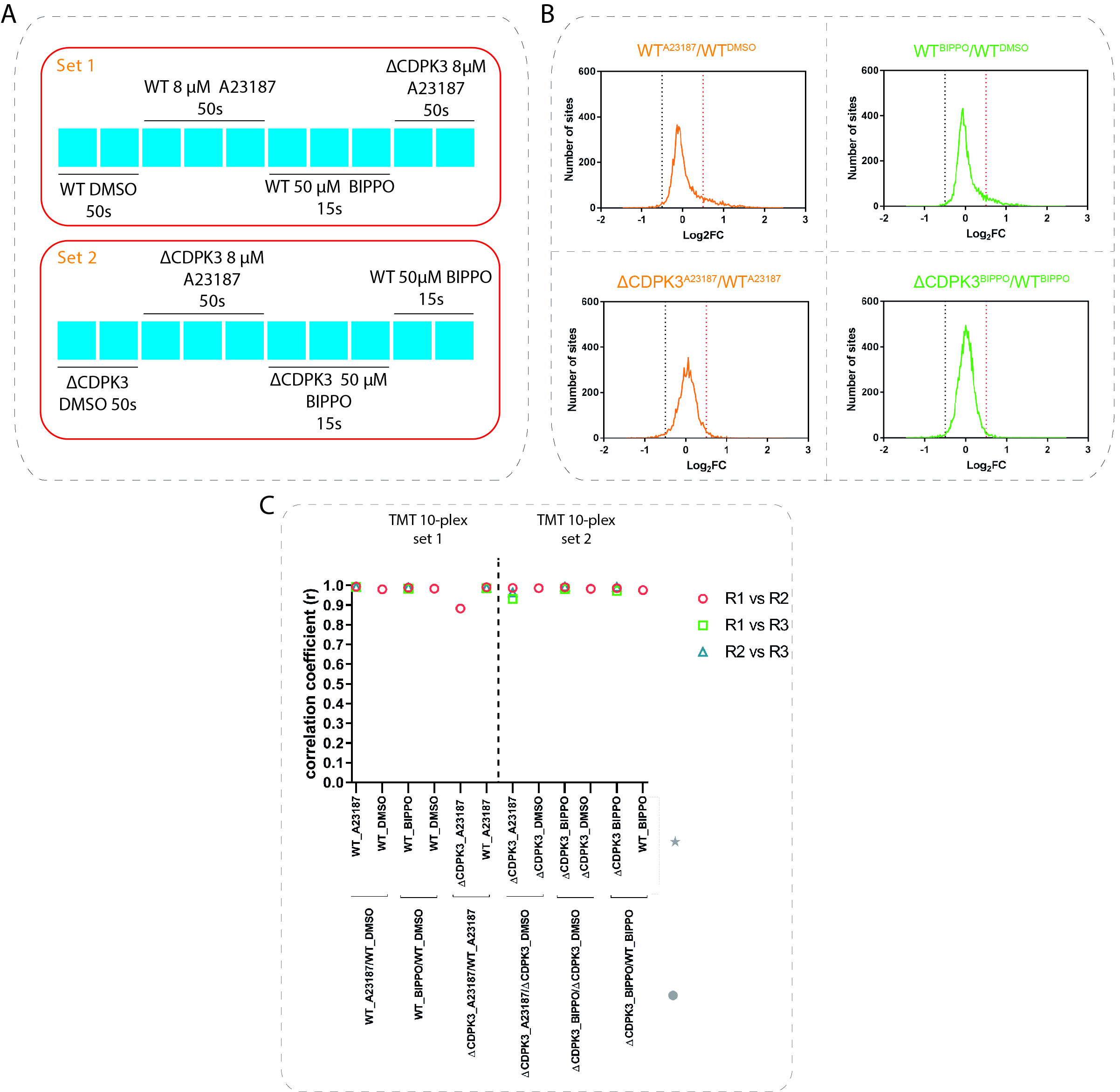

Supplement: S3 Fig — (A) Visual summary of the TMT-10-plex samples (sets 1 and 2) used to quantify the phosphoproteomes of intracellular WT and ΔCDPK3 tachyzoites treated with 50 μM BIPPO (15s) or 8 μM A23187 (50s). (B) Global phosphorylation responses (Log2FC frequency distribution) of WT and ΔCDPK3 tachyzoites following treatment with 8 μM A23187 (50s) or 50 μM BIPPO (15s). Dotted lines in graph represent 3xMAD outlier thresholds used to determine differential site regulation (log2FC>0.5 for up-regulated sites and log2FC<-0.5 for down-regulated sites). (C) Differentially regulated phosphosites were identified through the comparative analyses shown (grey circle). For these phosphosites, the reporter intensity scores for each sample replicate (grey star) were correlated, and the resulting Pearson’s correlation coefficients for each comparison are shown. R1, replicate 1; R2, replicate 2; R3, replicate 3. (JPG) [file ppat.1010901.s003.jpg]

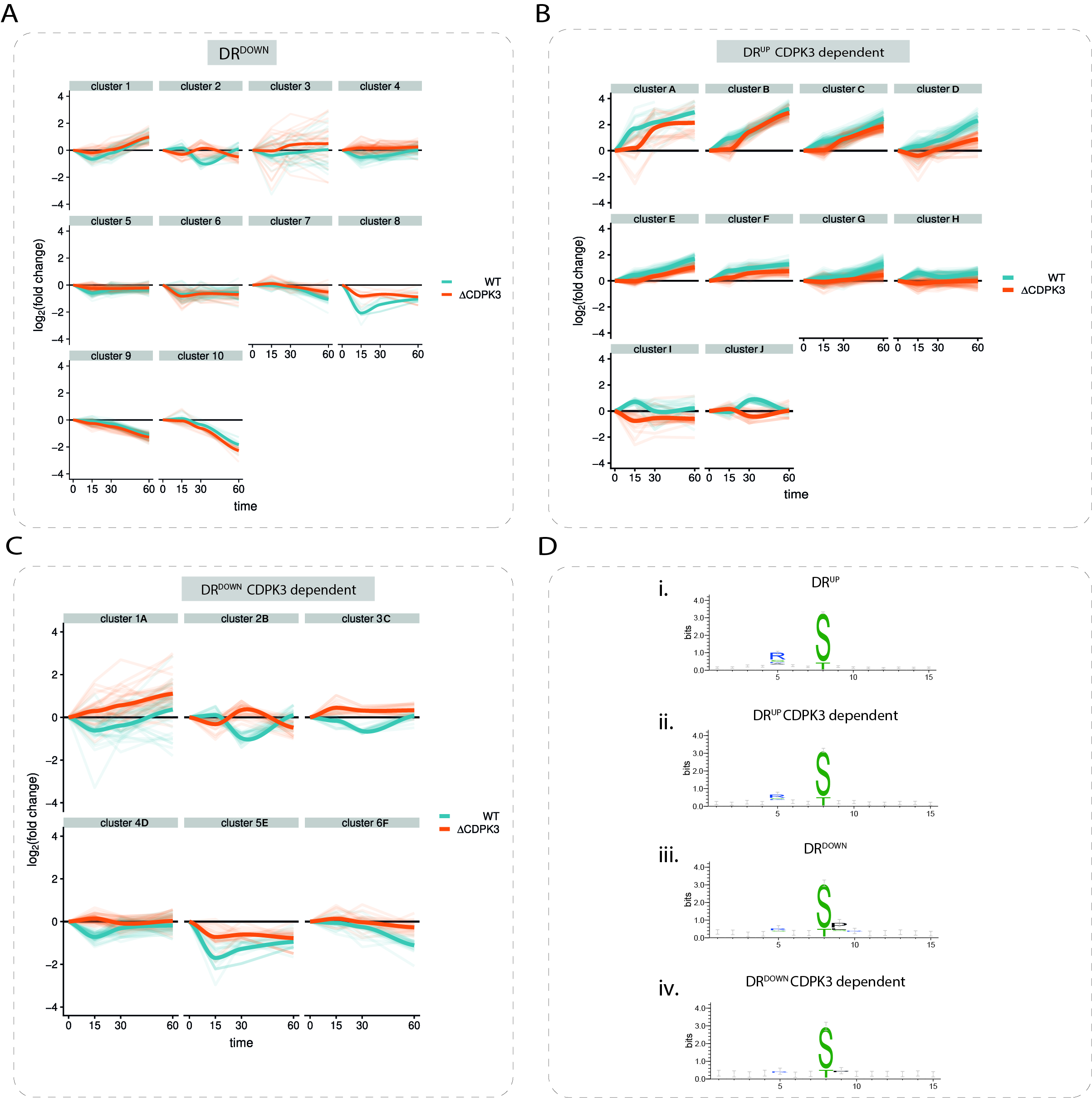

Supplement: S4 Fig — (A) Gaussian mixture-model-based clustering of DRDOWN sites in the A23187-treatment timecourses. Log2FC values from both WT and ΔCDPK3 samples were combined to cluster on six dimensions (WT 15s, 30s and 60s and ΔCDPK3 15s, 30s and 60s). Thin lines represent the timecourse traces of individual phosphorylation sites. Thick lines represent Loess regression fits of all traces. (B) Gaussian mixture-model-based clustering of DRUP and (C) DRDOWN CDPK3-dependent sites in the A23187-treatment timecourses. Log2FC values from both WT and ΔCDPK3 samples were combined to cluster on six dimensions (WT 15s, 30s and 60s and ΔCDPK3 15s, 30s and 60s). Thin lines represent the timecourse traces of individual phosphorylation sites. Thick lines represent Loess regression fits of all traces. (D) Results of phosphorylation motif enrichment analysis using rmotifx 1.0. Results are shown for the analysis of (i) DRUP (ii) DRUP CDPK3-dependent (iii)DRDOWN and (iv) DRDOWN CDPK3-dependent phosphorylation sites. (JPG) [file ppat.1010901.s004.jpg]

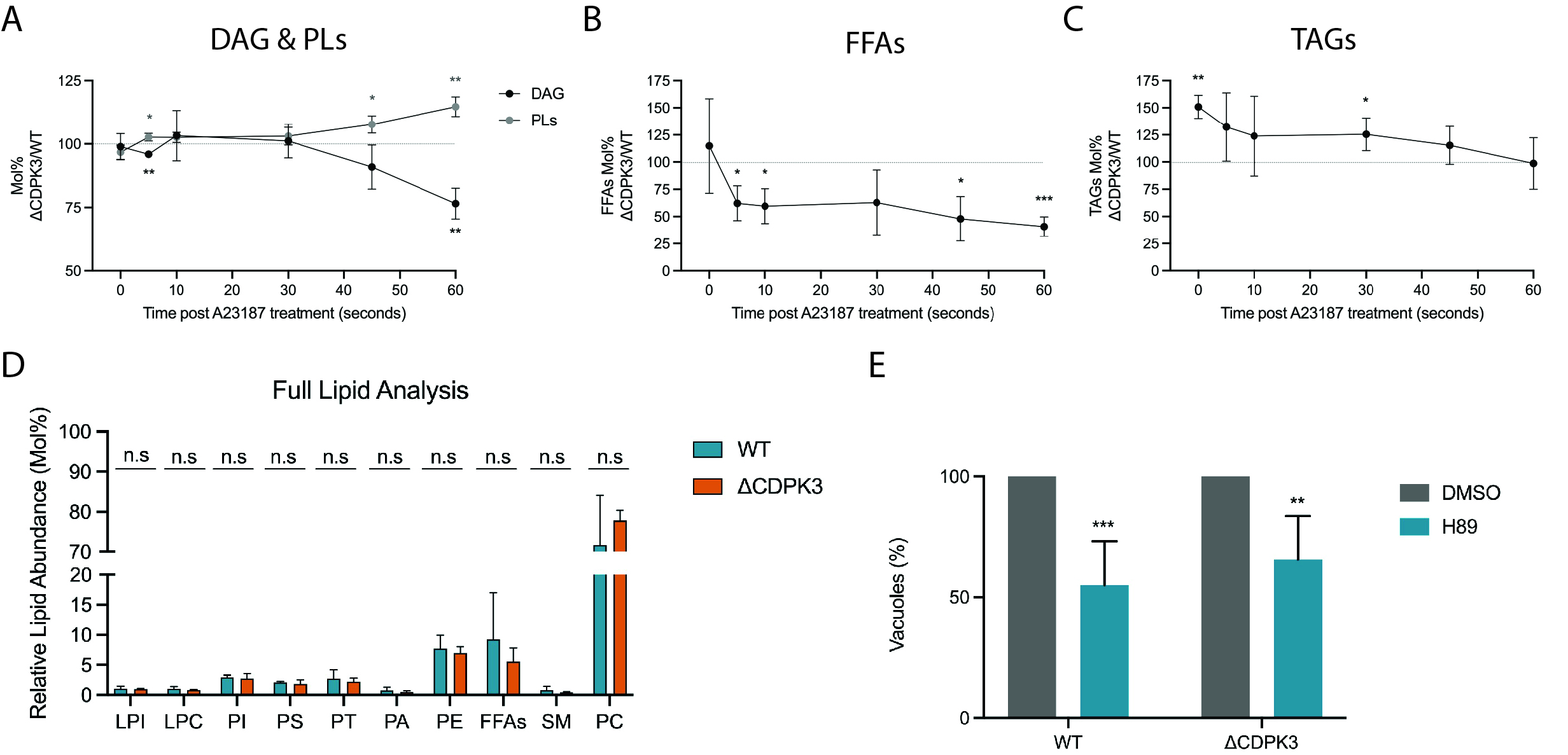

Supplement: S5 Fig — Pulse experiment of WT and ΔCDPK3 parasites treated with DMSO (0s) or 8 μM A23187 for 5, 10, 30, 45 or 60 seconds analysing levels of (A) DAG and PLs (B) FFAs and (C) TAGs, with data expressed as a ratio of ΔCDPK3/WT levels. Data are represented as mean ± s.d. (n = 4). Significance was assessed using a paired two sample t-test. **, P ≤ 0.01; *, P ≤ 0.05. (D) Phospholipid profile of WT and ΔCDPK3 extracellular parasites measuring the levels of lysophosphatidylinositol (LPI), lysophosphatidylcholine (LPC), phosphatidylinositol (PI), phosphatidylserine (PS), phosphatidylthreonine (PS), phosphatidic acid (PA), phosphatidylethanolamine (PE), free fatty acids (FA), sphingomyelin (SM) and phosphatidylcholine (PC). Data are represented as mean ± s.d. (n = 4). Significance was assessed using a paired t-test. n.s, not significant. (E) Quantification of natural egress of WT and ΔCDPK3 parasites following treatment with DMSO or 50 μM H89 (2 hrs). Graph shows the remaining % of un-egressed vacuoles (relative to untreated). Data are represented as mean ± s.d. (n = 5). Significance was assessed using an unpaired two-tailed t-test. ***, P ≤ 0.001; **, P ≤ 0.01. (JPG) [file ppat.1010901.s005.jpg]

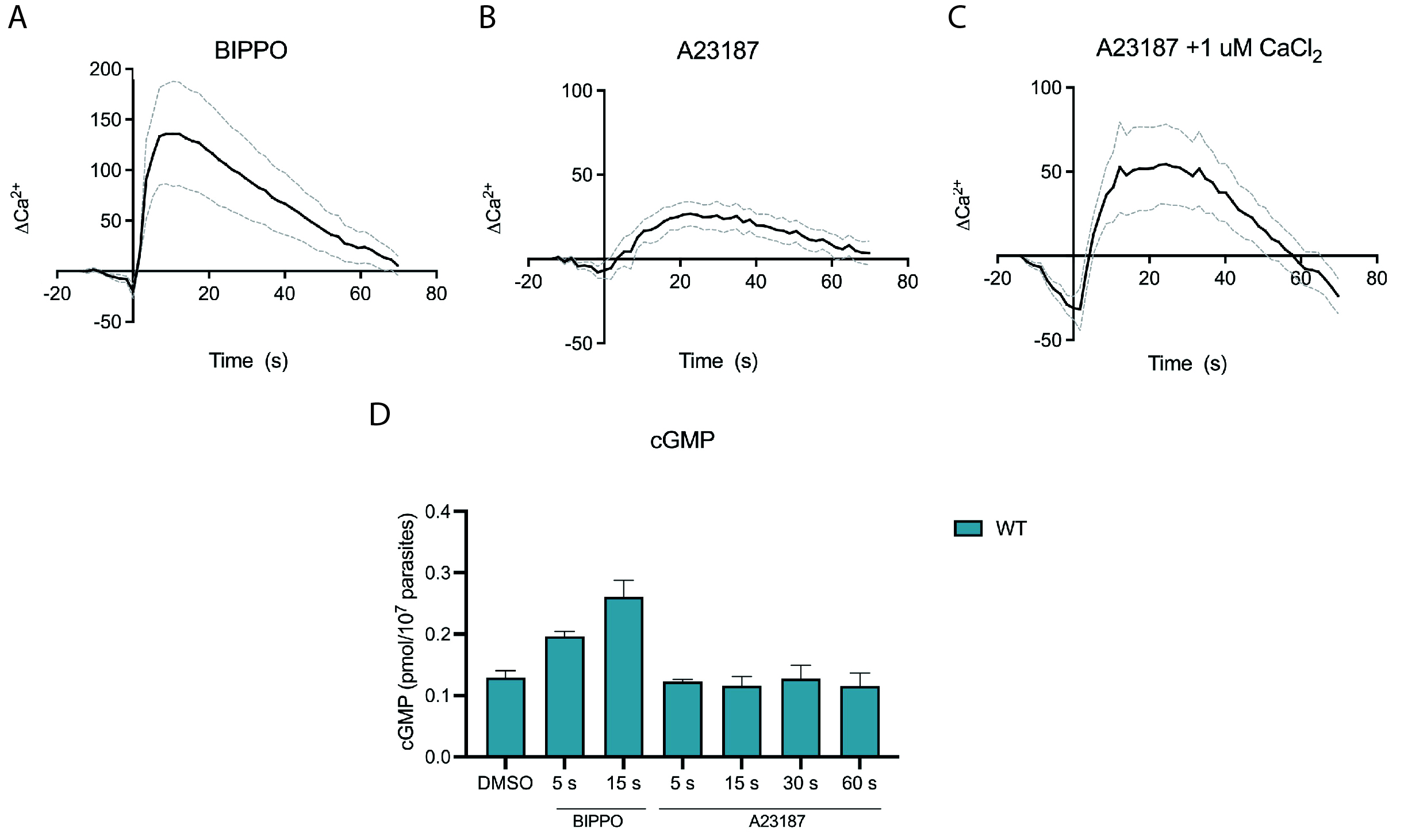

Supplement: S6 Fig — Ratiometric tracking of mean Ca2+ response (jRCaMP1b/GFP normalised to 0) of extracellular parasites in Endo buffer following addition of (A) 50μM BIPPO (B) 8 μM A23187 or (C) 8 μM A23187 with 1 μM CaCl2. Grey dotted lines represent ± SEM. Data was collected from ≥ 30 vacuoles (in separate wells) over ≥5 days. (D) cGMP levels extracellular WT tachyzoites in Endo buffer supplemented with 1 μM CaCl2 following treatment with DMSO for 60 seconds; 50 μM BIPPO for 5 or 15 seconds; or 8 μM A23187 for 5, 15, 30 or 60 seconds. All samples were lysed in 0.1 M HCl to inactivate all PDEs and cyclases, and extracts were analysed by using a commercial ELISA-based cGMP detection assay. Data are represented as mean ± s.d. (n = 4). (JPG) [file ppat.1010901.s006.jpg]

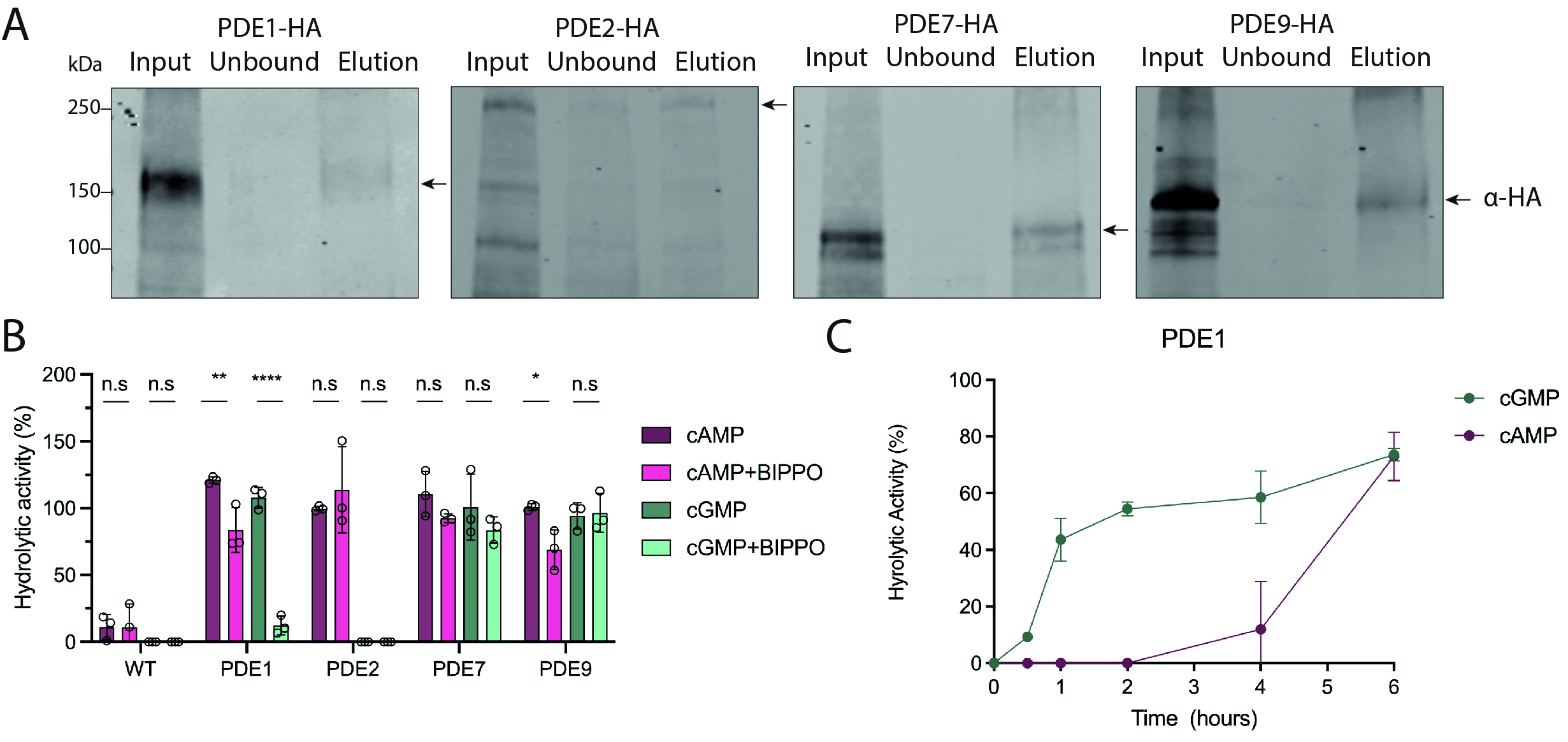

Supplement: S7 Fig — (A) Representative immunoprecipitation of PDEs 1, 2, 7 & 9 using α-HA magnetic beads probed with α-HA antibodies showing migration of the PDEs at their expected molecular weights as depicted by arrows. (B) Hydrolytic activity of immunoprecipitated HA-tagged PDE1, PDE2, PDE7, PDE9 using either 100 nM cAMP or 10 μM cGMP as a substrate. Lysates from the WT parental line were also included as a control. PDEs reactions were carried out in the presence of DMSO (vehicle) or 25 μM BIPPO for 2 hours at 37°C. Data are represented as mean ± s.d. (n = 3). Significance was assessed using a paired Two-way ANOVA. ****, P ≤ 0.0001; **, P ≤ 0.01; *, P ≤ 0.05; n.s. not significant. (C) Timecourse measuring the hydrolytic activity of immunoprecipitated HA-tagged PDE1 after incubating with either 1 μM cAMP or 10 μM cGMP at room temperature for the timepoints indicated. Data are represented as mean ± s.d. (n = 2). (JPG) [file ppat.1010901.s007.jpg]

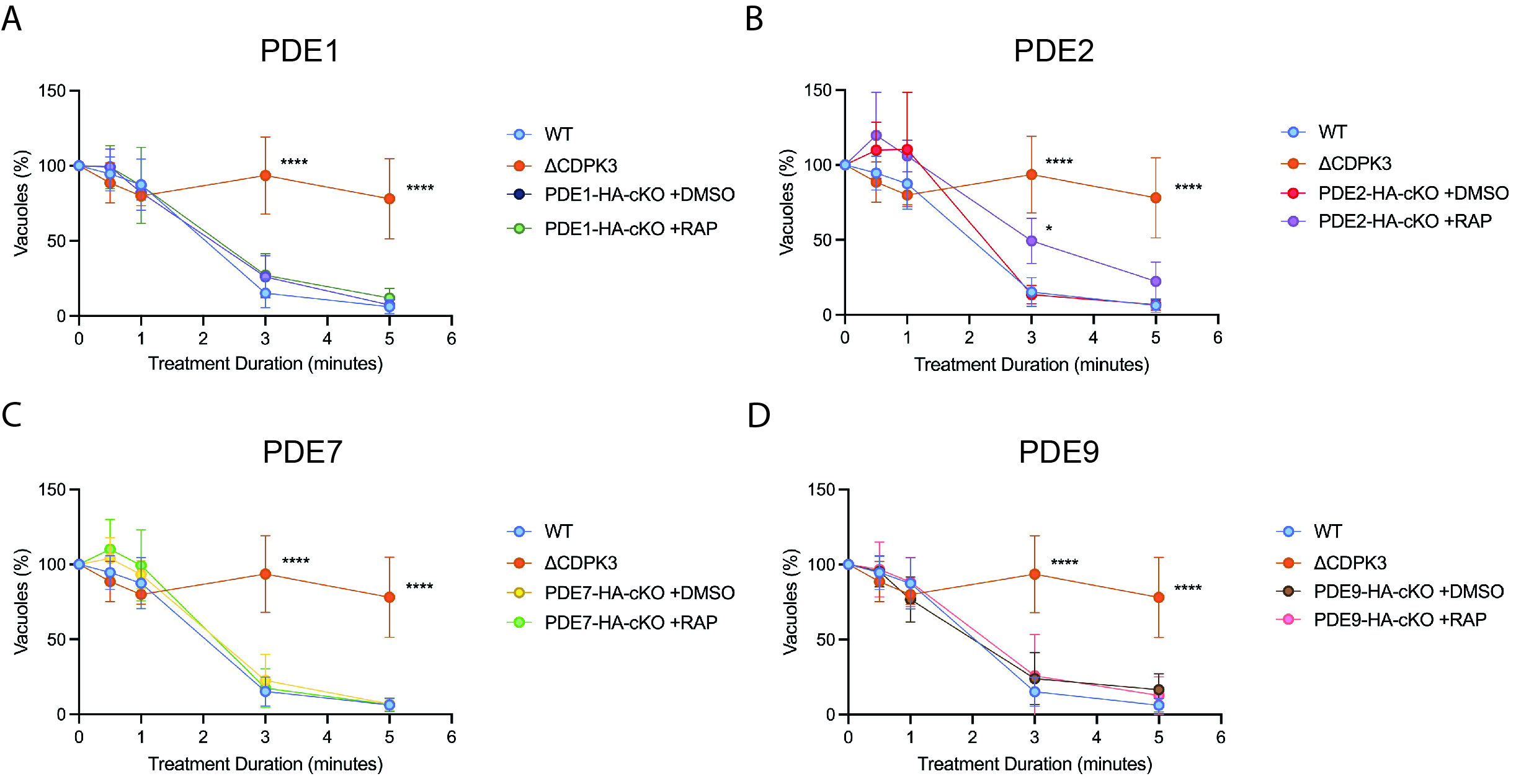

Supplement: S8 Fig — Egress assay of GFP-T2A-jRCaMP1b expressing WT, ΔCDPK3 and DMSO- and RAP-treated (A) PDE1, (B) PDE2, (C) PDE7 and (D) PDE9 cKO parasites following treatment with 8 μM A23187 after 0, 0.5, 1, 3, and 5 minutes. Data are represented as mean ± s.d. (n = 3). Two-way ANOVA.****, P ≤ 0.0001; *, P ≤ 0.05. (JPG) [file ppat.1010901.s008.jpg]
