# Supplementary material for: A positive feedback loop mediates crosstalk between calcium, cyclic nucleotide and lipid signalling in calcium-induced Toxoplasma gondii egress
Source: PLoS Pathog. 2022 Oct 20;18(10):e1010901. doi: 10.1371/journal.ppat.1010901 (PMC9624417; doi:10.1371/journal.ppat.1010901)
Supplement: Attachment — Submitted filename: 121903_1_rebuttal_2437671_rgntg1.pdf [file ppat.1010901.s018.pdf]

Full Revision

Manuscript number: RC-2021-01173

Corresponding author(s): Moritz Treeck

1. General Statements

Dear Editor,

We would like to transfer our manuscript, "**A positive feedback loop mediates crosstalk between calcium, cyclic nucleotide and lipid signalling in *Toxoplasma gondii***" after peer review in Review Commons.

The focus of the work lies on the investigation of how phospho-, cyclic nucleotide- and lipid-signalling pathways, utilised by the parasite *Toxoplasma gondii,* are interconnected. *Toxoplasma* is a member of a large group of related obligate intracellular parasites, including some of the most devastating human and livestock pathogens, which rely on successive rounds of host cell invasion and exit to proliferate in a host's body. Invasion and exit from the host cells are regulated by a set of plant-like calcium dependent kinases (CDPKs), which have no mammalian orthologues, as well as conserved signalling components of the cAMP-dependent protein kinase (PKA) and cGMP-dependent protein kinase (PKG) and lipid signalling pathways. While it is not well understood how parasites sense and respond to their environment, the aforementioned signalling pathways play a paramount role in this, allowing the parasites to rapidly leave a cell under attack from an immune cell or decide to transform into a latent or transmission stage. Thus, it is critical that we understand how these signalling pathways are connected. The various parasites differ widely in the number of CDPKs that they possess, as well as in the number of phosphodiesterases, (PDEs) which are required to generate cyclic AMP and GMP that activate the absolutely essential kinases PKA or PKG. This implies that there is a high degree of specialisation of how environmental signals are sensed and transformed into general phenotypic outputs. To investigate this in detail in a confined manner, we focused here on the long studied, but still poorly understood process of induced rapid exit of *Toxoplasma* from its host cell. We used two different stimuli of exit, a phosphodiesterase inhibitor thought to elevate cGMP, and a Calcium ionophore that activates CDPKs; and measured phospho-, lipid- and cyclic nucleotide signalling. We discovered a positive feedback loop that is regulated by the Calcium Dependent Kinase 3 (CDPK3), which was the focus of our and other groups' studies over the past years. We find that CDPK3 appears to sit central to this rapid feedback loop by controlling cAMP, a negative regulator of host cell egress and lipid signalling. Through the generation of 4 PDE conditional knockout parasites, we identify PDE2 as a cAMP hydrolysing PDE that contributes to egress regulation. This is the first description of a feedback loop involving all key signalling components studied so far and we believe of high importance for our understanding of the fine regulation of environmental sensing and responses in all parasites of this group. While we could not establish a direct link between CDPK3 and PDE2, our results strongly argue for an important role for both of these, linking the signalling pathways together. In addition to the biological findings in our study, our phospho-signalling timecourse provides a

substantial resource for the community and holds substantial promise for future targeted analysis of individual enzymes involved in the signalling pathways. We hope you share our enthusiasm for these findings and we look forward to hearing from you in due course.

2. Point-by-point description of the revisions

This section is mandatory. Please insert a point-by-point reply describing the revisions that were already carried out and included in the transferred manuscript.

Reviewer #1 (Evidence, reproducibility and clarity (Required)):

In recent years, the field has investigated crosstalk between cGMP and cAMP signaling (PMID: 29030485), lipid and cGMP signaling (PMID: 30742070), and calcium and cGMP signaling (PMID: 26933036, 26933037). In contrast to the Plasmodium field, which has benefited from proteomic experiments (ex: PMID 24594931, 26149123, 31075098, 30794532), second messenger crosstalk in T. gondii has been probed predominantly through genetic and pharmacological perturbations. The present manuscript compares the features of A23187- and BIPPO-stimulated phosphoproteomes at a snapshot in time. This is similar to a dataset generated by two of the authors in 2014 (PMID: 24945436), except that it now includes one BIPPO timepoint. The sub-minute phosphoproteomic timecourse following A23187 treatment in WT and ∆cdpk3 parasites is novel and would seem like a useful resource.

CDPK3-dependent sites were detected on adenylate cyclase, PI-PLC, guanylate cyclase, PDE1, and DGK1. This motivated study of lipid and cNMP levels following A23187 treatment. The four PDEs determined to have A23187-dependent phosphosites were characterized, including the two PDEs with CDPK3-dependent phosphorylation, which were found to be cGMP-specific. However, cGMP levels do not seem to differ in a CDPK3- or A23187-dependent manner. Instead, cAMP levels are elevated in ∆cdpk3 parasites. This would seem to implicate a feedback loop between CDPK3, the adenylyl cyclase, and PKA/PKG: CDPK3 activity reduces adenylyl cyclase activity, which reduces PKA activity, which increases PKG activity. The authors don't pursue this direction, and instead characterize PDE2, which does not have CDPK3-dependent phosphosites, and seems out of place in the study

Response:

We agree with reviewer 1 that a feedback loop between CDPK3, the adenylyl cyclase and PKA/PKG is certainly one of several possibilities (and we acknowledge this in the manuscript).

We felt, however, that given the observation that A23187 and BIPPO treatment leads to phosphorylation of numerous PDEs (hinting at the presence of an Ca^{2+}-regulated feedback loop), it was entirely relevant to study these in greater detail. Coupled with the A23187 egress assay on ΔPDE2 parasites - our findings suggest that PDE2 plays an important role in this signalling loop (an entirely novel finding). While PDE2 appears to exert its effects in a CDPK3-independent manner (indeed suggesting that CDPK3 might exert its effects on cAMP levels in a different fashion), this does not detract from the important finding that PDE2 is one of the (likely numerous) components that is regulated in a Ca^{2+}-dependent feedback loop to regulate egress.

We have modified our writing to better reflect the fact that our decision to pursue study of the PDEs was not solely CDPK3-centric.

While we feel that our reasoning for studying the PDEs is solid, we appreciate that further clarification on the putative CDPK3-Adenylate cyclase link would make it easier for the reader to follow the rationale.

We have not studied the direct link between CDPK3 and the Adenylate Cyclase β in more detail, as ACβ alone was shown to not play a major role in regulating lytic growth (Jia *et al.*, 2017).

****MAJOR COMMENTS****

1.Some of the key conclusions are not convincing.

The data presented in Figure 6E, F, and G and discussed in lines 647-679 are incongruent. In Figure 6E, the plaques in the PDE2+RAP image are hardly visible; how can it be that the plaques were accurately counted and determined not to differ from vehicle-treated parasites?

Are the images in 6E truly representative? Was the order of PDE1 and PDE2 switched? The cited publication by Moss et al. 2021 (preprint) is not in agreement with this study, as stated. That preprint determined that parasites depleted of PDE2 had significantly reduced plaque number and plaque size (>95% reduction); and parasites depleted of PDE1 had a substantially reduced plaque size but a less substantial reduction in plaque number.

Response:

The plaques for PDE2+RAP were counted using a microscope since they are difficult to see by eye. We thank the reviewer for detecting our incorrect reference to Moss *et al.* (2021). This has been corrected in the text. We confirm, however, that the images in 6E are representative of what we observed and do indeed differ from what was seen by Moss *et al.*. We have acknowledged this clearly in the text.

The differences cannot easily be explained other than by the different genetic systems used. Further studies of the individual PDEs will likely illuminate their role in invasion/ growth, but we feel this would be beyond the scope of this study.

Unfortunately, the length of time required for PDE depletion (72h) is incompatible with most T. gondii cellular assays (typically performed within one lytic cycle, 40-48h). Although the authors performed the assays 3 days after initial RAP treatment, is there evidence that non-excised parasites don't grow out of the population. This should be straightforward to test: treat, wait 3 days, infect onto monolayers, wait 24-48h fix, and stain with anti-YFP and an anti-Toxoplasma counterstain. The proportion of the parasite population that had excised the PDE at the time of the cellular assays will then be known, and the reader will have a sense of how complete the observed phenotypes are. As a reader, I will regard the phenotypes with some level of skepticism due to the long depletion time, especially since a panel of PDE rapid knockdown strains (depletion in < 18h) has recently been generated (Moss et al. 2021 preprint) using strains that have been used by several groups in the field since 2017 (PMID: 28465425). the experiments aren't wrong per se; the genetic system used here has substantial limitations, which are not appropriately controlled in the experiments or discussed in the text.

Response:

1. Cellular assays using KO parasites are commonly performed at the point at which protein depletion is detected. Both our western blots and plaque assay results demonstrate that, at the point of assay, there is no substantial outgrowth of non-excised parasites. The original manuscript also includes PCRs performed at the 72 hr time point (See Fig. 6B) to support this.

2. We appreciate the reviewer’s comment re the panel of PDE KD strains. The reviewer notes that there are substantial limitations to conditional KO systems, which similarly applies to KD systems - there are notable pros and cons to each approach. When

designing our strategy (pre-publication of the Moss *et al.*, 2022), we made a deliberate decision to use conditional KO strains in light of the fact that residual protein levels in KD systems can cause significant problems, particularly for membrane proteins (all of the investigated PDEs have a transmembrane domain). Tagging of proteins with the degradation domain can have further issues, leading to protein mis-localisation, which we have experienced with several unrelated proteins in the lab.

2. The authors should qualify some of their claims as preliminary or speculative, or remove them altogether.

The claims in lines 240-260 are confusing. It seems likely that the two drug treatments have at least topological distinctions in the signaling modules, given that cGMP-triggered calcium release is thought to occur at internal stores, whereas A23187-mediated calcium influx likely occurs first at the parasite plasma membrane.The authors' proposed alternative, that treatment-specific phosphosite behavior arises from experimental limitations and "mis-alignment", is unsatisfying for the following reasons: (1) From the outset, the authors chose different time frames to compare the two treatments (15s for BIPPO vs. 50s for A23187); (2) the experiment comprises a single time point, so it does not seem appropriate to compare the kinetics of phosphoregulation. There is still value in pointing out which phosphosites appear treatment-specific under the chosen thresholds, but further claims on the basis of this single-timepoint experiment are too speculative. Lines 264-267 and 281-284 should also be tempered.

Relatedly, graphing of the data in Figure 1G (accompanying the main text mentioned above) was confusing. Why is one axis a ratio, and the other log10 intensity? What does log10 intensity tell you without reference to the DMSO intensity? Wouldn't you want the L2FC(A23187) vs. L2FC(BIPPO) comparisons? Could you use different point colors to highlight these cases on plot 1E? Additionally, could you use a pseudocount to include peptides only identified in one treatment condition on the plot in 1E? (Especially since these sites are mentioned in lines 272-278 but are not on the plot)

Response:

1. The kinetics of the responses to A23187 and BIPPO are very different. This is why treatment timings are purposely different as they were selected to align pathways to a point where calcium levels peak just prior to calcium re-uptake. We make no mention of kinetic comparisons, and merely demonstrate that at the chosen timepoints, overall signalling correlation is very high. The observation that most of the sites that behave

differently between conditions sit remarkably close to the threshold for differential regulation (in the treatment condition where they are not DR - see Fig. 1G) led us to speculate that many of these sites are likely on the cusp of differential regulation. While it is entirely possible that some of these differences are, in fact, treatment specific (and we clearly acknowledge this in the text), we simply state that we cannot confidently discern clear signalling features that allow us to distinguish between the two treatments. We feel that this is an entirely relevant observation given the observed preponderance of both A23187 and BIPPO-dependent DR phosphosites on proteins in the PKG signalling pathway (as current models place this upstream of Ca^{2+} release).

2. Log10 intensity only serves to spread the data for easier visualisation. The only comparison being made relates to the LFCs. Fig. 1Gi shows the LFC scores (x axis) for all sites regulated following A23187 treatment (for which peptides were also identified in BIPPO treatment). On this plot we have highlighted the sites that are differentially regulated following BIPPO but not A23187 treatment (with red showing the DRup and blue showing the DRdown sites). This demonstrates that many of the sites that are regulated following BIPPO but not A23187 treatment cluster close to the threshold for differential regulation in the A23187 dataset - suggesting that many of these sites are likely on the cusp of differential regulation. Fig. 1Gii shows the reverse.

   While we could highlight the above-mentioned sites on the plot in Fig. 1E, we do not feel that it would demonstrate our point as clearly.

3. We feel that including a pseudocount on Fig. 1E for peptides lacking quantification in one treatment condition would be visually misleading as the direct correlation being made in Fig. 1E is BIPPO vs A23187 treatment. The sites mentioned in lines 272-278 in the original manuscript (now lines 268-276) are available in the supplement tables.

3.Additional experiments would be essential to support the main claims of the paper.

Genetic validation is necessary for the experiments performed with the PKA inhibitor H89. H89 is nonspecific even in mammalian systems (PMID: 18523239) and in this manuscript it was used at a high concentration (50 µM) The heterodimeric architecture of PKA in apicomplexans dramatically differs from the heterotetrameric enzymes characterized in metazoans (PMID: 29263246), so we don't know what the IC50 of the inhibitor is, or whether it inhibits competitively. Two inducible knockdown strains exist for PKA C1 (PMID: 29030485, 30208022).

The authors could request one of these strains and construct a ∆cdpk3 in that genetic background, as was done for the PDE2 cKO strain. Estimated time: 3-4 weeks to generate strain, 2 weeks to repeat assays.

Response:

1. While we appreciate that H89 is not 100% specific for PKA, this is not our only line of evidence that cAMP levels are altered. We demonstrate that cAMP levels are elevated in CDPK3 KO parasites – further substantiating our finding.

2. The H89 concentration used in our experiment is in keeping with/lower than the concentrations used in other *Toxoplasma* publications (Jia *et al.*, 2017), and both the *Toxoplasma* and *Plasmodium* fields have shown convincingly that H89 treatment phenocopies cKD/cKO of PKA (see Jia *et al.*, 2017; Flueck *et al.*, 2019).

3. While we agree that the genetic validation suggested by reviewer 1 would serve to further support our findings (though it would not provide further novel insights), the suggested time frame for experimental execution was not realistic. Line shipment, strain generation, subcloning and genetic validation would take substantially longer than 3-4 weeks.

cGMP levels are found to not increase with A23187 treatment, which is at odds with a previous study (lines 524-560). The text proposes that the differences could arise from the choice of buffer: this study used an intracellular-like Endo buffer (no added calcium, high potassium), whereas Stewart et al. 2017 used an extracellular-like buffer (DMEM, which also contains mM calcium and low potassium). An alternative explanation is that 60 s of A23187 treatment does not achieve a comparable amount of calcium flux as 15 s of BIPPO treatment, and a calcium-dependent effect on cGMP levels, were it to exist, could not be observed at the final timepoint in the assay. The experiments used to determine the kinetics of calcium flux following BIPPO and A23187 treatments (Fig. 1B, C) were calibrated using Ringer's buffer, which is more similar to an extracellular buffer (mM calcium, low potassium). In this buffer, A23187 treatment would likely stimulate calcium entry from across the parasite plasma membrane, as well as across the membranes of parasite intracellular calcium stores. By contrast, A23187 treatment in Endo buffer (low calcium) would likely only stimulate calcium release from intracellular stores, not calcium entry, since the calcium concentration outside of the parasite is low. Because calcium entry no longer contributes to calcium flux arising from A23187

treatment, it is possible that the calcium fluxes of A23187-treated parasites at 60 s are "behind" BIPPO-treated parasites at 15 s. The researchers could control these experiments by *either* (i) performing the cNMP measurements on parasites resuspended in the same buffer used in Figure 1B, C (Ringer's) or (ii) measuring calcium flux of extracellular parasites in Endo buffer with BIPPO and A23187 to determine the "alignment" of calcium levels, as was done with intracellular parasites in Figure 1C. No new strains would have to be generated and the assays have already been established in the manuscript. Estimated time to perform control experiments with replicates: 2 weeks. This seems like an important control, because the interpretation of this experiment shifts the focus of the paper from feedback between calcium and cGMP signaling, which had motivated the initial phosphoproteomics comparisons, to calcium and cAMP signaling. Further, the lipidomics experiments were performed in an extracellular-like buffer, DMEM, so it's unclear why dramatically different buffers were used for the lipidomics and cNMP measurements.

Response:

While the initial calibration experiments to measure calcium flux were indeed performed in Ringer's buffer, the parasites were intracellular. We therefore chose to measure cNMP concentrations of extracellular parasites syringe lysed in Endo buffer, which is better at mimicking intracellular conditions than any other described buffer.

As the reviewer suggested, we measured the calcium flux of extracellular parasites in Endo buffer upon stimulation with either A23187 or BIPPO.

We found that peak calcium response to BIPPO in Endo buffer was similar to that of intracellular parasites (~15 seconds post treatment) (See Supp Fig. 6A). Upon treatment with A23187, extracellular parasites in Endo buffer had a much faster response compared to their intracellular counterparts, with peak flux measured at ~25 seconds post treatment (see Supp Fig. 6B). This indeed does suggest that extracellular parasites in Endo buffer behave differently to A23187 compared to their intracellular counterparts. However, peak calcium response is still occuring within the experimental time course and is not being missed, as the reviewer worries. Moreover, since we are able to detect increased cAMP levels in A23187 treated parasites, Ca^{2+} flux appears sufficient to alter cNMP signalling.

We did notice however that the intensity of the calcium flux was much weaker in Endo buffer compared to intracellular parasites (see Supp Fig. 6B). We found that this was due

to the lack of host-derived Ca^{2+}, since supplementation of Endo buffer with 1 uM $CaCl_2$ restored the intensity of the calcium response to match that of intracellular parasites (see Supp Fig. 6C). We therefore decided to repeat our cGMP measurements, this time using extracellular parasites in Endo buffer supplemented with 1 uM $CaCl_2$. However, we found no differences in cGMP levels in the response to ionophore under these conditions (now Supp Fig. 6D) compared to the previous experiments, so the conclusions from the previous data do not change.

As for the lipidomics experiments, we chose to use DMEM so that our dataset could be compared with other published lipidomic datasets (Katris *et al.*, 2020; Dass *et al.*, 2021) where DMEM was also used as a buffer when measuring global lipid profiles of parasites.

We now acknowledge in the paper that Endo buffer has its shortcomings, and that this could be the reason why we do not detect changes in cGMP concentrations. We do, however, believe that Endo buffer is the best alternative to intracellular parasites and is supported by its consistent use in numerous publications studying *Toxoplasma* signalling (McCoy *et al.*, 2012; Stewart *et al.*, 2017).

Additional information is required to support the claim that PDE2 has a moderate egress defect (lines 681-687). T. gondii egress is MOI-dependent (PMID: 29030485). Although the parasite strains were used at the same MOI, there is no guarantee that the parasites successfully invaded and replicated. If parasites lacking PDE2 are defective in invasion or replication, the MOI is effectively decreased, which could explain the egress delay. Could the authors compare the MOIs (number of vacuoles per host cell nuclei) of the vehicle and RAP-treated parasites at t = 0 treatment duration to give the reader a sense of whether the MOIs are comparable?

Response:

Since PDE2 KO parasites have a substantial growth defect, we did notice that starting MOIs were consistently lower for the RAP-treated samples compared to the DMSO-treated samples. However, this was also the case for PDE1 KO parasites where we did not see an egress delay. We also found that the egress delay was still evident for ΔCDPK3 parasites, despite having higher starting MOIs than WT parasites in our experiments. Therefore there does not appear to be a link between starting MOIs and the egress delay.

To be sure of our results, we also performed egress assays where we co-infected HFFs with mCherry-expressing WT parasites (WT ΔUPRT) and GFP-expressing PDE2 cKO parasites that were treated with either DMSO or RAP or ΔCDPK3 parasites. This recapitulated our previous findings, confirming the deletion of PDE2 leads to delay in A23187-mediated egress.

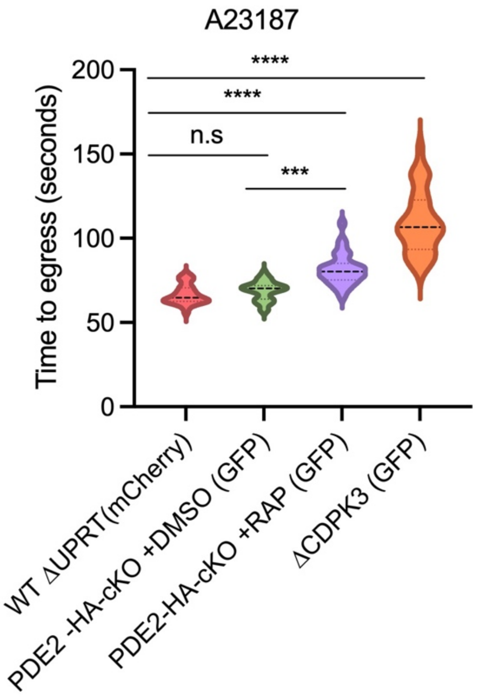

Egress assay quantifying the timing of parasite egress following stimulation with 8 uM A23187. Parasites expressing mCherry from the UPRT locus (called WT ΔUPRT) were seeded with GFP-expressing ΔCDPK3, DMSO-treated PDE2-HA-cKO or RAP-treated PDE2-HA-cKO parasites on a monolayer of confluent HFF cells grown on IBIDI tissue culture treated 8 well chamber slides and allowed to grow for 28 hrs in DMEM + 10%FBS. Prior to imaging, wells were washed once with PBS, and supplemented with 100 µl Ringer's buffer. Imaging was performed on the Nikon Eclipse Ti-U inverted fluorescent microscope, 60x/1.4 NA Oil immersion objective, in an environmental chamber (OKOLAB) with temperature maintained at 37°C. Image capture was managed by Nikon NIS-Elements software with acquisition 1/s for 200s. At 15s following image acquisition, 100µl of A23187 (24µM) in Ringer's buffer was added by pipette to achieve a final concentration of 8 µM. ≥30 vacuoles were imaged across ≥ 3 days for each condition. Image analysis was performed using ImageJ. The average timing of WT ΔUPRT parasite egress was used for normalisation between experiments.

4.A few references are missing to ensure reproducibility.

The manuscript states that the kinetic lipidomics experiments were performed with established methods, but the cited publication (line 497) is a preprint. These are therefore not peer reviewed and should be described in greater detail in this manuscript, including any relevant validation.

Response:

We thank the reviewer for pointing this out. We have included a greater description of the methods used in the materials and methods section such that the experiment is reproducible, as per the reviewer's suggestion. We decided to still make mention of the BioRxiv preprint since we thought it was appropriate for the reader to be informed of ongoing developments in the field.

Please cite the release of the T. gondii proteomes used for spectrum matching (lines 972-973).

Response:

We have included this as per the reviewer's suggestion.

Please include the TMT labeling scheme so the analysis may be reproduced from the raw files.

Response:

We have included this as per the reviewer's suggestion in Supp Fig. 3A.

5.Statistical analyses should be reviewed as follows:

Have the authors examined the possibility that some changes in phosphopeptide abundance reflect changes in protein abundance? This may be particularly relevant for comparisons involving the ∆cdpk3 strain. Did the authors collect paired unenriched proteomes from the experiments performed? Alternatively, there may be enriched peptides that did not change in abundance for many of the proteins that appear dynamically phosphorylated.

Response:

We did not collect unenriched proteomes from the experiments performed (although we did perform unenriched mixing checks to ensure equal loading between samples), and believe that this wasn't a necessity for the following reasons:

1. For within-line treatment analyses, treatment timings are so short (a maximum of 15-50s in the single timepoint experiment) that it would be unlikely to detect substantial changes in protein abundance. Moreover, these unlikely events would affect all phosphosites across a protein, and therefore be detectable.

2. In our CDPK3 dependency timecourse experiments, we normalise both the WT and ΔCDPK3 strain to 0s, and measure signalling progression over time. Therefore, any difference at timepoints that are not "0" are not originating from basal differences. We also see a consistent increase/decrease in phosphosite detection across the sub-minute timecourse, further confirming that the observed changes are truly down to dynamic changes in phosphorylation and not protein levels.

3. In the single timepoint CDPK3 dependency analyses (44 regulated sites identified, Data S2), we acknowledge that there could be some risk of altered starting protein abundance between lines. However, if protein abundance were responsible for the changes in phosphosite detection, we would expect all phosphosites across the protein to shift, and we do not observe this. Moreover, when we look at these CDPK3 dependent proteins and compare their phosphosite abundance in untreated WT and ΔCDPK3 lines, we find that for each protein, either all or the majority of phosphosites detected are unchanged (highlighting that there is no substantial difference in this protein's abundance between lines). Where there are phosphosite differences between lines, these are only ever on single sites on a protein while most other sites are unchanged - implying that these are changes to basal phosphorylation states and not protein levels.

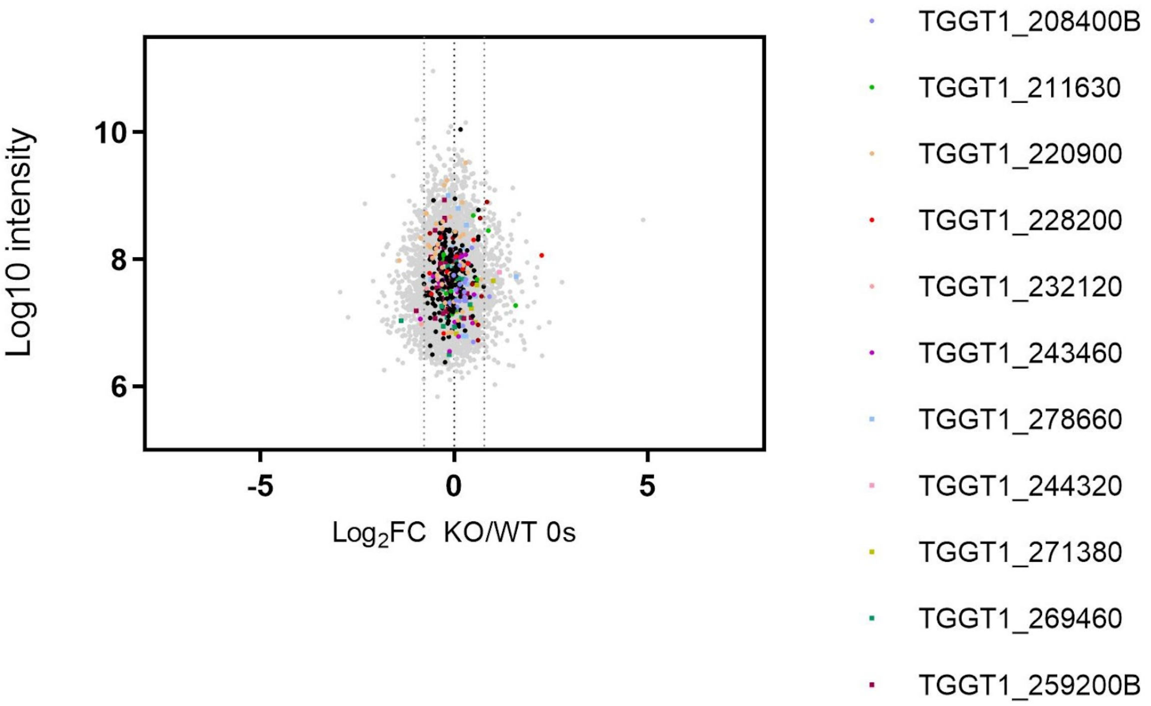

KO/WT log_2FC vs log_{10} total reporter intensity in untreated lines. Overlaid in black and colour are phosphosites on the CDPK3 dependent proteins from the IONO/BIPPO single timepoint experiment (Data S2). If none of the sites on these proteins are changed, the associated phosphosites are all shown in black. Where there are single phosphosites that pass the 3xMAD (0.78) threshold, all sites on this protein are shown in the same colour.

It seems like for Figs. 3B and S5 the maximum number of clusters modeled was selected. Could the authors provide a rationale for the number of clusters selected, since it appears many of the clusters have similar profiles.

The number of clusters is chosen automatically by the Mclust algorithm as the value that maximizes the Bayes Information Criterion (BIC). BIC in effect balances gains in model fit (increasing log-likelihood) against increasing the number of parameters (i.e. number of clusters).

Please include figure panel(s) relating to gene ontology. Relevant information for readers to make conclusions includes p-value, fold-enrichment or gene ratio, and some sort of metric of the frequency of the GO term in the surveyed data set. See PMID: 33053376 Fig. 7 and PMID: 29724925 Fig. 6 for examples or enrichment summaries. Additionally, in the methods, specify (i) the background set, (ii) the method used for multiple test correction, (iii) the criteria constituting "enrichment",

(iv) how the T. gondii genome was integrated into the analysis, (v) the class of GO terms (molecular function, biological process, or cellular component), (vi) any additional information required to reproduce the results (for example, settings modified from default).

Response:

We have included the additional information requested in the materials and methods.

We purposely did not include GO figure panels as our analyses are being done across many clusters, making it very difficult to display this information cohesively. We have included all data in Tables S2-S5. These tables included all the relevant information on p-value, enrichment status, ratio in study/ratio in population, class of GO terms etc.

The presentation of the lipidomics experiments in Figure 4A-C is confusing. First, the ∆cdpk3/WT ratio removes information about the process in WT parasites, and it's unclear why the scale centers on 100 and not 1. Second, the data in Figure S6 suggests a more modest effect than that represented in Fig. 4; is this due to day to day variability? How do the authors justify pairing WT and mutant samples as they did to generate the ratios?

This is a common strategy used by many metabolomics experts (Bailey *et al.*, 2015; Dass *et al.*, 2021; Lunghi *et al.*, 2022). We had originally chosen to represent the data as a ratio since this form of representation helps get rid of the variability that arises between experiments and allows us to see very clear patterns which would otherwise go unnoticed. This variability arises from the amount of lipids in each sample which varies between parasites in a dish, the batch of FBS and DMEM used, and the solutions and even room temperature used to extract lipids on a given day.

However, we agree with the reviewer that depicting the data in Figure 4A-C as a ratio of ∆CDPK3/WT parasites can be confusing, so we have now changed the graphs, plotting WT and ∆CDPK3 levels instead, and have moved the ratio of ∆CDPK3/WT to the Supplementary Figure 5.

The significance test seems to be performed on the difference between the WT and ∆cdpk3 strains, but not relative to the DMSO treatment? Wouldn't you want to perform a repeated measures ANOVA to determine (i) if lipid levels change over time and (ii) if this trend differs in WT vs. mutant strain?

The reviewer correctly points out that ANOVA is often used for time courses, but we must point out that it is not always strictly appropriate since it can overlook the purpose of the individual experiment design, which in this case is, 1) to investigate the role of CDPK3 compared to the WT parental strain, and 2) specifically to find the exact point at which the DAG begins to change after stimulus to match the proteomics time course.

Our data is clearly biassed towards earlier time points where we have 0, 5, 10, 30, 45 seconds where DAG levels are mostly unchanged compared to the single timepoint 60 seconds which shows a significant difference in DAG using our method of statistical comparison by paired two tailed t-test. Therefore, it would be unwise to use ANOVA when we really want to see when the A23187 stimulus takes effect, which appears to be after the 45 second mark. Therefore, analysing the data by ANOVA would likely provide a false negative result, where the result is non-significant but there is clearly more DAG in WT than CDPK3 after 60 seconds. T-tests are commonly used when comparing the same cell lines grown in the same conditions with a test/treatment, and in this case the test/treatment is CPDK3 present or absent (Lentini *et al.*, 2020).

In the main text, it would be preferable to see the data presented as the proteomics experiments were in Figure 4B and 4C, with fold changes relative to the DMSO (t = 0) treatment, separately for WT and ∆cdpk3 parasites.

We have now changed the way that we represent the data, plotting %mol instead of the ratio.

Signaling lipids constitute small percentages of the overall pool (e.g. PMID: 26962945), so one might not necessarily expect to observe large changes in lipid abundance when signaling pathways are modulated. Is there any positive control that the authors could include to give readers a sense of the dynamic range? Maybe the DGK1 mutant (PMID: 26962945)?

DGK1 is maybe not a good example because the DGK1 KO parasites effectively “melt” from a lack of plasma membrane integrity ((Bullen *et al.*, 2016), so this would likely be technically challenging. We don’t see the added value in including an additional mutant control since we can already see the dynamic change over time from no difference (0 seconds) to significant difference (60 seconds) between WT and CDPK3 for DAG and most other lipids. We already see a significant difference between WT and CDPK3 after 60 seconds for DAG, and we can clearly see in sub-minute timecourses the changes or not at the specific points where the A23187 is added (0-5 seconds), the parasites

acclimatise, for the A23187 to take effect (10-30 seconds) and for the parasite lipid response to be visible by lipidomics (45-60 +seconds).

Figure 4E: are the differences in [cAMP] with DMSO treatment and A23187 treatment different at any of the timepoints in the WT strain? The comparison seems to be WT/∆cdpk3 at each timepoint. Does the text (lines 562-568) need to be modified accordingly?

Response

In WT (and ∆CDPK3) parasites, [cAMP] is significantly changed at 5s of A23187 treatment (relative to DMSO). We have modified our figures to include this analysis. The existing text accurately reflects this.

Figure 6I: is the difference between PDE2 cKO/∆cdpk3 + DMSO or RAP significant?

Response

In our original manuscript, there was no statistical difference in [cAMP] between PDE2cKO/∆CDPK3+DMSO and PDE2cKO/∆CDPK3+DMSO+RAP, likely due to the variation between biological replicates. To overcome the issues in variability between replicates, we have now included more biological replicates (n=7). This has led to a significant difference in [cAMP] between PDE2cKO/∆CDPK3 DMSO- and RAP-treated parasites and between PDE2cKO DMSO- and RAP-treated parasites (now Fig. 6I).

****MINOR COMMENTS****

1.The following references should be added or amended:

Lines 83-85: in the cited publication, relative phosphopeptide abundances of an overexpressed dominant-negative, constitutively inactive PKA mutant were compared to an overexpressed wild-type mutant. In this experimental setup, one would hypothesize that targets of PKA should be down-regulated (inactive/WT ratios). However, the mentioned phosphopeptide of PDE2 was found to be up-regulated, suggesting that it is not a direct target of PKA.

Response:

We thank the reviewer for spotting this error, we have now modified our wording.

Cite TGGT1_305050, referenced as calmodulin in line 458, as TgELC2 (PMID: 26374117).

Response:

We have included this as per the reviewer's suggestion.

Cite TGGT1_295850 as apical annuli protein 2 (AAP2, PMID: 31470470).

Response:

We have included this as per the reviewer's suggestion.

Cite TGGT1_270865 (adenylyl cyclase beta, Acβ) as PMID: 29030485, 30449726.

Response:

We have included this as per the reviewer's suggestion.

Cite TGGT1_254370 (guanylyl cyclase, GC) as PMID: 30449726, 30742070.

Response:

We have included this as per the reviewer's suggestion.

Note that Lourido, Tang and David Sibley, 2012 observed that treatment with zaprinast (a PDE inhibitor) could overcome CDPK3 inhibition. The target(s) of zaprinast have not been determined and may differ from those of BIPPO (in identity and IC50). The cited study also used modified CDPK3 and CDPK1 alleles, rather than ∆cdpk3 and intact cdpk1 as used in this manuscript. That is to say, the signaling backgrounds of the parasite strains deviate in ways that are not controlled.

Response:

While it is true that zaprinast targets have not been unequivocally identified, zaprinast-induced egress is widely thought to be the result of PKG activation, a conclusion that is further supported by the finding that Compound 1 completely blocks zaprinast-induced egress (Lourido, Tang and David Sibley, 2012). Similarly, BIPPO-induced egress is

inhibited by chemical inhibition of PKG by Compound 1 and Compound 2 (Jia *et al.*, 2017). Moreover, like zaprinast, BIPPO has been clearly shown to partially overcome the ΔCDPK3 egress delay (Stewart *et al.*, 2017).

2.The following comments refer to the figures and legends:

Part of the legend text for 1G is included under 1H.

Response:

This has been corrected

Figure 1H: The legend mentions that some dots are blue, but they appear green. Please ensure that color choices conform to journal accessibility guidelines. See the following article about visualization for colorblind readers: https://www.ascb.org/science-news/how-to-make-scientific-figures-accessible-to-readers-with-color-blindness/ . Avoid using red and green false-colored images; replace red with a magenta lookup table. Multi-colored images are only helpful for the merged image; otherwise, we discern grayscale better. Applies to Figures 1B, 5C, 6D. (Aside: anti-CAP seems an odd choice of counterstain; the variation in the staining, esp. at the apical cap, is distracting.)

Response:

We thank reviewer #1 for bringing this to our attention, and have modified our colour usage for all IFAs and Figures 1H and 3E.

We chose CAP staining as the antibody is available in the laboratory and stains both the apical end (which has been shown to contain several proteins important for signalling as well as PDE9) and the parasite periphery, the location of CDPK3.

Figure 1B: When showing a single fluorophore, please use grayscale and include an intensity scale bar, since relative values are being compared.

Response:

We have modified this as per the reviewer's suggestion

Figure 1C: it is difficult to compare the kinetics of the calcium response when the curves are plotted separately. Since the scales are the same, could the two treatments be plotted on the same axes, with different colors? Additionally, according to the legend, a red line seems to be missing in this panel.

Response:

Fig1C is not intended to compare kinetics, merely to show peak calcium release in each separate treatment condition. We have removed mention of a red line in the figure legend.

Figure 2A: Either Figure S4 can be moved to accompany Figure 2A, or Figure 2A could be moved to the supplemental.

Figure S4 has now been incorporated into Figure 2.

Reviewer #1 (Significance (Required)):

This manuscript would interest researchers studying signaling pathways in protozoan parasites, especially apicomplexans, as CDPK3 and PKG orthologs exist across the phylum. To my knowledge, it is the first study that has proposed a mechanism by which a calcium effector regulates cAMP levels in T. gondii. Unfortunately, the experiments fall short of testing this mechanism.

We thank reviewer #1 for their comments, but disagree with their assessment that the key points of the manuscript “fall short of experimental testing”.

1. We demonstrate that, following both BIPPO and A23187 treatment, there is differential phosphorylation of numerous components traditionally believed to sit upstream of PKG activation (as well as several components within the PKG signalling pathway itself).
2. We show that some of these sites are CDPK3 dependent, and that deletion of CDPK3 leads to changes in lipid signalling and an elevation in levels of cAMP (dysregulation of which is known to alter PKG signalling).
3. We show that pre-treatment with a PKA inhibitor is able to largely rescue this phenotype.
4. We demonstrate that a cAMP-specific PDE is phosphorylated following A23187 treatment (i.e. Ca^{2+} flux)
5. We show that this cAMP specific PDE plays a role in A23187-mediated egress.

6. While the latter PDE may not be directly regulated by CDPK3, these findings suggest that there are likely several Ca^{2+}-dependent kinases that contribute to this feedback loop.

Reviewer #2 (Evidence, reproducibility and clarity (Required)):

****Summary:****

Provide a short summary of the findings and key conclusions (including methodology and model system(s) where appropriate).

In this manuscript, Dominicus et al investigate the elusive role of calcium-dependent kinase 3 during the egress of Toxoplasma gondii. Multiple functions have already been proposed for this kinase by this group including the regulation of basal calcium levels (24945436) or of a tyrosine transporter (30402958). However, one of the most puzzling phenotypes of CDPK3 deficient tachyzoites is a marked delay in egress when parasites are stimulated with a calcium ionophore that is rescued with phosphodiesterase (PDE) inhibitors. Crosstalk between, cAMP, cGMP, lipid and calcium signalling has been previously described to be important in regulating egress (26933036, 23149386, 29030485) but the role of CDPK3 in Toxoplasma is still poorly understood.

Here the authors first take an elegant phosphoproteomic approach to identify pathways differentially regulated upon treatment with either a PDE inhibitor (BIPPO) and a calcium ionophore (A23187) in WT and CDPK3-KO parasites. Not much difference is observed between BIPPO or A23187 stimulation which is interpreted by the authors as a regulation through a feed-back loop.

The authors then investigate the effect of CDPK3 deletion on lipid, cGMP and cAMP levels. The identify major changes in DAG, phospholipid, FFAs, and TAG levels as well as differences in cAMP levels but not for cGMP. Chemical inhibition of PKA leads to a similar egress timing in CDPK3-KO and WT parasites upon A23187 stimulation.

As four PDEs appeared differentially regulated in the CDPK3-KO line upon A23187, the authors investigate the requirement of the 4 PDEs in cAMP levels. They show diverse localisation of the PDEs with specificities of PDE1, 7 and 9 for cGMP and of PDE2 for cAMP. They further show that PDE1, 7 and 9 are sensitive to BIPPO. Finally, using a conditional deletion system, they show that PDE1 and 2 are important for the lytic cycle of Toxoplasma and that PDE2 shows a slightly delayed egress following A23187 stimulation.

****Major comments:****

-Are the key conclusions convincing?

The title is supported by the findings presented in this study. However I am not sure to understand why the authors imply a positive feed back loop. This should be clarified in the discussion of the results.

Response:

We believe in a positive feedback loop as, upon A23187 treatment (resulting in a calcium flux), ΔCDPK3 parasites are able to egress, albeit in a delayed manner. This egress delay is substantially, but not completely, alleviated upon treatment with BIPPO (a PDE inhibitor known to activate the PKG signalling pathway). In conjunction with our phosphoproteomic data (where we see phosphorylation of numerous pathway components upstream of PKG upon BIPPO and A23187 treatment - both in a CDPK3 dependent and independent manner), these observations suggest that calcium-regulated proteins (CDPK3 among them) feed into the PKG pathway. As deletion of CDPK3 delays egress, it is reasonable to postulate that this feedback is one that amplifies egress signalling (i.e. is positive).

The phosphoproteome analysis seems very strong and will be of interest for many groups working on egress. However, the key conclusion, i.e. that a substrate overlaps between PKG and CDPK3 is unlikely to explain the CDPK3 phenotype, seems premature to me in the absence of robustly identified substrates for both kinases.

Response:

We certainly do not fully exclude the possibility of a substrate overlap but do lean more heavily towards a feedback loop given (a) the inability to clearly detect treatment-specific signalling profiles and (b) the phospho targets observed in the A23187 and BIPPO phosphoproteomes. We have further clarified our reasoning, and overall tempered our language in the manuscript as per the reviewer's suggestion.

I am not sure there is a clear key conclusion from the lipidomic analysis and how it is used by the authors to build their model up. Major changes are observed but how could this be linked with CDPK3, particularly if cGMP levels are not affected?

Our phosphoproteomic analyses identify several CDPK3-dependent phospho sites on phospholipid signalling components (DGK1 & PI-PLC), suggesting that there is indeed altered signalling downstream of PKG. To test whether these lead to a measurable phenotype, we performed the lipidomics analysis. We did not pursue this arm of the signalling pathway any further as we postulated that the changes in the lipid signalling pathway were less likely to play a role in the feedback loop. Nevertheless, we felt that it was worthwhile to include these findings in our manuscript as they support the conclusions drawn from the phosphoproteomics - namely that lipid signalling is perturbed in CDPK3 mutants. We, or others, may follow up on this in future.

We agree with the reviewer that it is surprising that cGMP levels remain unchanged in our experiments when we treat with A23187. Given the measurable difference in cAMP levels between WT and ΔCDPK3 parasites, we postulate that CDPK3 directly or indirectly downregulates levels of cAMP. This would, in turn, alter activity of the cAMP-dependent protein kinase PKAc. Jia *et al. (2017)* have shown a clear dependency on PKG for parasites to egress upon PKAc depletion, but were also unable to reliably demonstrate cGMP accumulation in intracellular parasites. Similarly, their hypothesis that dysregulated cGMP-specific PDE activity results in altered cGMP levels has not been proven (the PDE hypothesised to be involved has since been shown to be cAMP-specific).

While it is possible that our collective inability to observe elevated cGMP levels is explained by the sensitivity limits of the assay, it is similarly possible that cAMP-mediated signalling is exerting its effects on the PKG signalling pathway in a cGMP-independent manner.

The evidence that CDPK3 is involved in cAMP homeostasis seems strong. However, the analysis of PKA inhibition is a bit less clear. The way the data is presented makes it difficult to see whether the treatment is accelerating egress of CDPK3-KO parasites or affecting both WT and CDPK3-KO lines, including both the

speed and extent of egress. This is important for the interpretation of the experiment.

Response:

Fig. 4F shows that there is a significant amount of premature egress in both WT and ΔCDPK3 parasites following 2 hrs of H89 pre-treatment (consistent with previous reports that downregulation of cAMP signalling stimulates premature egress). When we subsequently investigated A23187-induced egress rates of the remaining intracellular H89 pre-treated parasites (Fig. 4Gi-ii) we found that the ΔCDPK3 egress delay was largely rescued. We have moved Fig. 4F to the supplement (now Supp Fig. 5E) in order to avoid confusion between the distinct analyses shown in 4F (pre-treatment analyses) and 4G (egress experiment). These experiments provided a hint that cAMP signalling is affected, which we then validate by measuring elevated cAMP levels in CDPK3 mutant parasites.

The biochemical characterisation of the four PDE is interesting and seems well performed. However, PDE1 was previously shown to hydrolyse both cAMP and cGMP (https://doi.org/10.1101/2021.09.21.461320) which raises some questions about the experimental set up. Could the authors possibly discuss why they do not observe similar selectivity? Could other PDEs in the immunoprecipitate mask PDE activity? In line with this question, it is not clear what % of "hydrolytic activity (%)" means and how it was calculated.

The experiments describing the selectivity of BIPPO for PDE1, 7 and 9 as well as the biological requirement of the four tested PDEs are convincing.

Response:

We believe that the disagreement between our findings and those published by Moss and colleagues are due to the differences in experimental conditions. We performed our assays at room temperature for 1 hour with higher starting cAMP concentrations (1 uM) compared to them. They performed their assays at 37ºC for 2 hours with 10-fold lower starting cAMP concentrations (0.1 uM). We have now repeated this set of experiments using the Moss *et al.* conditions, and find that PDEs 1, 7 and 9 can be dual specific, while PDE2 is cAMP-specific, thereby recapitulating their findings (Now included in the revised manuscript under Supp Fig. 7B). However, we also now performed a timecourse PDE assay using our original conditions and show that the cAMP hydrolytic activity for PDE1 can only be detected following 4 hours of incubation, compared to cGMP activity that can be detected as early as 30 minutes, suggesting that it possesses predominantly cGMP activity (See Supp Fig. 7C). We therefore believe that our experimental setup is more stringent, because if one starts with a lower level of substrate and incubates for longer

and at a higher temperature, even minor dual activity could make a substantial difference in cAMP levels. Our data suggests that the cAMP hydrolytic activity of PDEs 1, 7 and 9 is substantially lower than the cGMP hydrolytic activity that they display.

We have also included a clear description of how % hydrolytic activity was calculated in the methods section.

-Should the authors qualify some of their claims as preliminary or speculative, or remove them altogether?

The claim that CDPK3 affects cAMP levels seems strong however the exact links between CDPK3 activity, lipid, cGMP and cAMP signalling remain unclear and it may be important to clearly state this.

Response:

We have modified our wording in the text to more clearly describe our current hypothesis and reasoning.

-Would additional experiments be essential to support the claims of the paper? Request additional experiments only where necessary for the paper as it is, and do not ask authors to open new lines of experimentation.

I think that the manuscript contains a significant amount of experiments that are of interest to scientists working on Toxoplasma egress. Requesting experiments to identify the functional link between above-mentioned pathways would be out of the scope for this work although it would considerably increase the impact of this manuscript. For example, would it be possible to test whether the CDPK3-KO line is more or less sensitive to PKG specific inhibition upon A23187 induced?

-Are the suggested experiments realistic in terms of time and resources? It would help if you could add an estimated cost and time investment for substantial experiments.

The above-mentioned experiment is not trivial as no specific inhibitors of PKG are available. Ensuring for specificity of the investigated phenotype would require the generation of a resistant line which would require significant work.

Response: We agree that this would be an interesting experiment to further substantiate our findings. As indicated by the reviewer, however, the lack of specific inhibitors of PKG means a resistant line would likely be required to ensure specificity.

-Are the data and the methods presented in such a way that they can be reproduced?

It is not clear how the % of hydrolytic activity of the PDE has been calculated.

Response: We have included a clearer description of how % hydrolytic activity was calculated in the methods section.

-Are the experiments adequately replicated and statistical analysis adequate?

This seems to be performed to high standards.

****Minor comments:****

-Specific experimental issues that are easily addressable.

I do not have any comments related to minor experimental issues.

-Are prior studies referenced appropriately?

Most of the studies relevant for this work are cited. It is however not clear to me why some important players of the "PKG pathway" are not indicated in Fig 1H and Fig 3E, including for example UGO or SPARK.

Response:

We have modified Fig 1H and 3E to include all key players involved in the PKG pathway.

-Are the text and figures clear and accurate?

While all the data shown here is impressive and well analysed, I find it difficult to read the manuscript and establish links between sections of the papers. The phosphoproteome analysis is interesting and is used to orientate the reader towards a feedback mechanism rather than a substrate overlap. But why do the

authors later focus on PDEs and not on AC or CNBD, as in the end, if I understand well, there is no evidence showing a link between CDPK3-dependent phosphorylation and PDE activity upon A23187 stimulation?

Response:

We thank reviewer#2 and appreciate their constructive feedback re the flow of the manuscript.

Our key findings from the phosphoproteomics study were that 1) BIPPO and A23187 treatment trigger near identical signalling pathways, 2) that both A23187 and BIPPO treatment leads to phosphorylation of numerous components both upstream and downstream of PKG signalling (hinting at the presence of an Ca^{2+}-regulated feedback loop) and 3) several of the abovementioned components are phosphorylated in a CDPK3 dependent manner.

While several avenues of study could have been pursued from this point onwards, we chose to focus on the feedback loop in a broader sense as its existence has important implications for our general understanding of the signalling pathways that govern egress.

We reasoned that, given the differential phosphorylation of 4 PDEs following A23187 and BIPPO treatment (none of which had been studied in detail previously), it was relevant to study these in greater detail.

Coupled with the A23187 egress assay on PDE2 knockout parasites - our findings suggest that PDE2 plays a role in the abovementioned Ca^{2+} signalling loop. While PDE2 may not exert its effects in a CDPK3-dependent manner (and CDPK3 may, therefore, alter cAMP levels in a different fashion), this does not detract from the important finding that PDE2 is one of the (likely numerous) components that is regulated in a Ca^{2+}-dependent feedback loop to facilitate rapid egress.

We have modified our wording to better reflect our rationale for studying the PDEs irrespective of their CDPK3 phosphorylation status.

While we feel that our reasoning for studying the PDEs is solid, we do appreciate that further clarification on the putative CDPK3-Adenylate cyclase link would elevate the manuscript substantially. However, given the data that the ACb is not playing a sole role in the control of egress, this is likely a non-trivial task and requires substantial work.

It is also unclear how the authors link CDPK3-dependent elevated cAMP levels with the elevated basal calcium levels they previously described. This is particularly difficult to reconcile particularly in a PKG independent manner.

Response:

We previously postulated that elevated Ca^{2+} levels allowed ΔCDPK3 mutants to overcome a complete egress defect, potentially by activating other CDPKs (e.g. CDPK1). It is similarly plausible that elevated Ca^{2+} levels in ΔCDPK3 parasites may lead to elevated cAMP levels in order to prevent premature egress.

As noted in our previous responses, we acknowledge that our inability to detect cGMP is surprising. However, given the clarity of our cAMP findings, and the phosphoproteomic evidence to suggest that various components in the PKG signalling pathway are affected, we postulate that we are either unable to reliably detect cGMP due to sensitivity issues, or that cAMP is exerting its regulation on the PKG pathway in a cGMP-independent manner. As noted previously, while the link between cAMP and PKG signalling has been demonstrated by Jia *et al.*, it is not entirely clear how this is mediated.

The presentation of the lipidomic analysis is also not really clear to me. Why do the authors show the global changes in phospholipids and not a more detailed analysis?

Response:

We performed a detailed phospholipid profile of WT and ΔCDPK3 parasites under normal culture conditions. However, due to the sheer quantity of parasites required for this detailed analysis, we were unable to measure individual phospholipid species in our A23187 timecourse. We therefore opted to measure global changes following A23187 stimulation.

As the authors focus on the PI-PLC pathway, could they detail the dynamics of phosphoinositides? I understand that lipid levels are affected in the mutant but I am not sure to understand how the authors interpret these massive changes in relationship with the function of CDPK3 and the observed phenotypes.

Response:

Our phosphoproteomic analyses identified several CDPK3-dependent phospho sites on phospholipid signalling components (DGK1 & PI-PLC), suggesting that (in keeping with

all of our other data), there is altered signalling downstream of PKG. To test whether these changes lead to a measurable phenotype, we performed the lipidomics analysis. Following stimulation with A23187, we found a delayed production of DAG in ΔCDPK3 parasites compared to WT parasites. Since DAG is required for the production of PA, which in turn is required for microneme secretion, our finding can explain why microneme secretion is delayed in ΔCDPK3 parasites, as previously reported (Lourido, Tang and David Sibley, 2012; McCoy *et al.*, 2012).

We did not follow this arm of the signalling pathway any further as we postulated that the changes in the lipid signalling pathway were less likely to play a role in the feedback loop. Nevertheless, we felt that it was worthwhile to include these findings in our manuscript as they support the conclusions drawn from the phosphoproteomics - namely that lipid signalling is perturbed in CDPK3 mutants. We, or others, may follow up on this in future.

Finally, the characterisation of the PDEs is an impressive piece of work but the functional link with CDPK3 is relatively unclear. It would also be important to clearly discuss the differences with previous results presented in this this preprint: https://doi.org/10.1101/2021.09.21.461320.

My understanding is while the authors aim at investigating the role of CDPK3 in A23187 induced egress, the main finding related to CDPK3 is a defect in cAMP homeostasis that is not linked to A23187. Similarly, the requirements of PDE2 in cAMP homeostasis and egress is indirectly linked to CDPK3. Altogether I think that important results are presented here but divided into three main and distinct sections: the phosphoproteomic survey, the lipidomic and cAMP level investigation, and the characterisation of the four PDEs. However, the link between each section is relatively weak and the way the results are presented is somehow misleading or confusing.

Response:

As mentioned in a previous response, we chose to study PDEs in greater detail because of our observation that both A23187 and BIPPO treatments lead to their phosphorylation (hinting at the presence of a Ca^{2+} regulated feedback loop). We were particularly intrigued to study the cAMP specific PDE, as CDPK3 KO parasites suggested that cAMP may play a role in the Ca^{2+} feedback mechanism. As PDE2 may not be directly regulated by CDPK3, Ca^{2+} appears to exert its feedback effects in numerous ways. We have modified our wording to better reflect our rationale for studying the PDEs irrespective of their CDPK3 phosphorylation status.

-Do you have suggestions that would help the authors improve the presentation of their data and conclusions?

This is a very long manuscript written for specialists of this signalling pathway and I would suggest the authors to emphasise more the important results and also clearly state where links are still missing. This is obviously a complex pathway and one cannot elucidate it easily in a single manuscript.

Response:

We have included an additional summary in our conclusions to better illustrate our findings and clarify any missing links.

Reviewer #2 (Significance (Required)):

-Describe the nature and significance of the advance (e.g. conceptual, technical, clinical) for the field.

This is a technically remarkable paper using a broad range of analyses performed to a high standard.

-Place the work in the context of the existing literature (provide references, where appropriate).

The cross-talk between cAMP, cGMP and calcium signalling is well described in Toxoplasma and related parasites. Here the authors show that, in Toxoplasma, CDPK3 is part of this complex signalling network. One of the most important finding within this context is the role of CDPK3 in cAMP homeostasis. With this in mind, I would change the last sentence of the abstract to "In summary we uncover a feedback loop that enhances signalling during egress and links CDPK3 with several signalling pathways together."

Response:

In light of feedback received from several reviewers, we have made our wording less CDPK3 centric - as our findings relate in part to CDPK3 and, in a broader sense, to a Ca^{2+} driven feedback loop.

Review COMMONS

The genetic and biochemical analyses of the four PDEs are remarkable and highlight consistencies and inconsistencies with recently published work that would be important to discuss and will be of interest for the field.

Response: We thank reviewer#2 and agree that the PDE findings are of significant importance to the field.

While I understand the studied signalling pathway is complex, I think it would be important to better describe the current model of the authors. In the discussion, the authors indicate that "the published data is not currently supported by a model that fits most experimental results." I would suggest to clarify this statement and discuss whether their work helps to reunite, correct or improve previous models.

Response: We have expanded on the abovementioned statement to clarify that the presence of a feedback loop is a major pillar of knowledge required for the complete interpretation of existing signalling data.

Could the authors also speculate about a potential role of PDE/CDPK3 in host cell invasion as cAMP signalling has be shown to be important for this process (30208022 and 29030485)?

Response: Existing literature (Jia *et al.*, 2017) suggests that perturbations to cAMP signalling play a very minor role in invasion since parasites where either ACα or ACβ are deleted show no impairment in invasion levels. We currently do not have substantial data on invasion, and are not sure that pursuing this is valuable given the minor phenotypes observed in other studies.

-State what audience might be interested in and influenced by the reported findings.

This paper is of great interest to groups working on the regulation of egress in Toxoplasma gondii and other related apicomplexan pathogens.

-Define your field of expertise with a few keywords to help the authors contextualize your point of view. Indicate if there are any parts of the paper that you do not have sufficient expertise to evaluate.

I am working on the cell biology of apicomplexan parasites.

Reviewer #3 (Evidence, reproducibility and clarity (Required)):

Summary:

Dominicus et al aimed to identify the intersecting components of calcium, cyclic nucleotides (cAMP, cGMP) and lipid signaling through phosphoproteomic, knockout and biochemical assays in an intracellular parasite, Toxoplasma gondii, particularly when its acutely-infectious tachyzoite stage exits the host cells. A series of experimental strategies were applied to identify potential substrates of calcium-dependent protein kinase 3 (CDPK3), which has previously been reported to control the tachyzoite egress. According to earlier studies (PMID: 23226109, 24945436, 5418062, 26544049, 30402958), CDPK3 regulated the parasite exit through multiple phosphorylation events. Here, authors identified differentially-regulated (DR) phosphorylation sites by comparing the parasite samples after treatment with a calcium ionophore (A23178) and a PDE inhibitor (BIPPO), both of which are known to induce artificial egress (induced egress as opposed to natural egress). When the DCDPK3 mutant was treated with A23187, its delayed egress phenotype did not change, whereas BIPPO restored the egress to the level of the parental (termed as WT) strain, probably by activating PKG.

The gene ontology enrichment of the up-regulated clusters revealed many probable CDPK3-dependent DR sites involved in cyclic nucleotide signaling (PDE1, PDE2, PDE7, PDE9, guanylate and adenylate cyclases, cyclic nucleotide-binding protein or CNBP) as well as lipid signaling (PI-PLC, DGK1). Authors suggest lipid signaling as one of the factors altered in the CDPK3 mutant, albeit lipidomics (PC, PI, PS, PT, PA, PE, SM) showed no significant change in phospholipids. To reveal how the four PDEs indicated above contribute to the cAMP and cGMP-mediated egress, they examined their biological significance by knockout/knockdown and enzyme activity assays. Authors claim that PDE1,7,9 proteins are cGMP-specific while PDE2 is cAMP-specific, and BIPPO treatment can inhibit PDE1-cGMP and PDE7-cGMP, but not PDE9-cGMP. Given the complexity, the manuscript is well structured, and most experiments were carefully designed. Undoubtedly, there is a significant amount of work that underlies this manuscript; however, from a

conceptual viewpoint, the manuscript does not offer significant advancement over the current knowledge without functional validation of phosphoproteomics data (see below). A large body of work preceding this manuscript has indicated the crosstalk of cAMP, cGMP, calcium and lipid signaling cascades. This work provides a further refinement of the existing model In a methodical sense, the work uses established assays, some of which require revisiting to reach robust conclusions and avoid misinterpretation. The article is quite interesting from a throughput screening point of view, but it clearly lacks the appropriate endorsement of the hits.The authors accept that identifying the phosphorylation of a protein does not imply a functional role, which is a major drawback as there is no experimental support for any phosphorylation site of the protein identified through phosphoproteomics. In terms of the mechanism, it is not clear whether and how lipid turnover and cAMP-PKA signaling control the egress phenotype (lack of a validated model at the end of this study).

Response:

We thank reviewer #3 for their comments, but respectfully disagree with their assessment that the work presented does not advance current knowledge.

1. We demonstrate that, following both BIPPO and A23187 treatment, there is differential phosphorylation of numerous components traditionally believed to sit upstream of PKG activation (as well as numerous components within the PKG signalling pathway itself). While it may have been inferred from previous studies that A23187 and BIPPO signalling intersect, this has never been unequivocally demonstrated - nor has a feedback loop ever been shown.

2. We provide a novel A23187-driven phosphoproteome timecourse that further bolsters the model of a Ca^{2+}-driven feedback loop.

3. We show that deletion of CDPK3 leads to a delay in DAG production upon stimulation with A23187.

4. We show that some of the abovementioned sites are CDPK3 dependent, and that deletion of CDPK3 leads to elevated levels of cAMP (dysregulation of which is known to alter PKG signalling).

5. We show that pre-treatment with a PKA inhibitor is able to largely rescue this phenotype.

6. We demonstrate that a cAMP-specific PDE is phosphorylated following A23187 treatment (i.e. Ca^{2+} flux)

7. We show that this cAMP specific PDE plays a role in egress.

8. While the latter PDE may not be directly regulated by CDPK3, these findings suggest that there are likely several Ca^{2+}-dependent kinases that contribute to this feedback loop.

We also firmly disagree with the reviewer's assertion that without phosphosite characterisation, we have no support for our model. Following treatment with A23187 (and BIPPO), we clearly show broad, systemic changes (both CDPK3 dependent and independent) across signalling pathways previously deemed to sit upstream of calcium flux. Given the vast number of proteins involved in these signalling pathways, and the multitude of differentially regulated phosphosites identified on each of them, it is highly likely that the signalling effects we observe are combinatorial. Accordingly, we believe that mutating individual sites on individual proteins would be a very costly endeavour which is unlikely to substantially advance our understanding of signalling during egress. Moreover, introducing multiple point mutations in a given protein to ablate phosphorylation may lead to protein misfolding and would therefore not be informative. One of the key aims of this study was to assess how egress signalling pathways are interconnected, and we believe we have been able to show strong support for a Ca^{2+}-driven feedback mechanism in which both CDPK3 and PDE2 play a role through the regulation of cAMP.

While we agree with the reviewer's statement that a large body of work preceding this manuscript has indicated the crosstalk of cAMP, cGMP, calcium and lipid signalling cascades, a feedback loop has not previously been shown. We believe that this finding is absolutely central to facilitate the complete interpretation of existing signalling data. Furthermore, no previous studies have gone to this level of detail in either proteomics or lipidomics to analyse the calcium signal pathway in any apicomplexan parasite. We argue that the novelty in our manuscript is that it is a carefully orchestrated study that advances our understanding of the signalling network over time with subcellular precision. The kinetics of signalling is not well understood and we believe that our study is likely the first to include both proteomic and lipidomic analyses over a timecourse during the acute lytic cycle stage of the disease. In doing so, we found evidence for a feedback loop that

controls the signalling network spatiotemporally, and we characterise elements of this feedback in the same study.

Major Comments:

Based on the findings reported here there is little doubt that BIPPO and A23187-induced signaling intersect with each other, as very much expected from previous studies. The authors selected the 50s and 15s post-treatment timing of A23187 and BIPPO, respectively for collecting phosphoproteomics samples. At these time points, which were shown to peak cytosolic Ca2+, parasites were still intracellular (Line #171). How did authors make sure to stimulate the entire signaling cascade adequately, particularly when parasites do not egress within the selected time window? There is significant variability between phosphosite intensities of replicates (Line #186), which may also be attributed to insufficient triggers for the egress across independent experiments. This work must be supported by in vitro egress assays with the chosen incubation periods of BIPPO and ionophore treatment (show the induced % egress of tachyzoites in the 50s and 15s).

Response:

1. We appreciate that the reviewer acknowledges that our data clearly shows that BIPPO and A23187-induced signalling intersect. While this may have been expected from previous studies, this has not previously been shown - and is therefore valuable to the field. Specifically, the fact that A23187-treatment leads to phosphorylation of targets normally deemed to sit upstream of calcium release is entirely novel and adds a substantial layer of information to our understanding of how these signalling pathways work together.

2. Treatments were purposely selected to align pathways to a point where calcium levels peak just prior to calcium reuptake. At these chosen timepoints, we clearly show that overall signalling correlation is very high. We know from our egress assays using identical treatment concentrations (Fig. 2C), that the stimulations used are sufficient to result in complete egress. We are simply comparing signalling pathways at points prior to egress.

3. As mentioned in point 2, we show convincingly that the treatments used are sufficient to trigger complete egress. As detailed clearly in the text, we believe that these variations in intensities between replicates are due to slight differences in timing

between experiments (this is inevitable given the very rapid progression of signalling, and the difficulty of replicating exact sub-minute treatment timings). We demonstrate that the reporter intensities associated with DR sites correlate well across replicates (Supp Fig. 3C), suggesting that despite some replicate variability, the overall trends across replicates is very much consistent. This allows us to confidently average scores to provide values that are representative of a site's phosphorylation state at the timepoint of interest.

4. The reviewer's suggestion that we should demonstrate % egress at the 50s and 15s treatment timepoints is obsolete - we state clearly in the text that parasites have not egressed at these timepoints. Our egress assays (Fig. 2C) further support this.

The authors discuss that CDPK3 controls the cAMP level and PKA through activation of one or more yet-to-be-identified PDEs(s). cAMP could probably also be regulated by an adenylate cyclase, ACbeta that was found to have CDPK3-dependent phosphorylation sites. If CDPK3 is indeed a regulator of cAMP through the activation of PDEs or ACbeta, it would be expected that the deletion of CDPK3 would perturb the cAMP level, resulting in dysregulation of PKAc1 subunit, which in turn would dysregulate cGMP-specific PDEs (PMID: 29030485) and thereby PKG. All these connections need to explain in a more clear manner with experimental support (what is positive and what is negatively regulated by CDPK3).

Response:

1. We do not firmly state that CDPK3 regulates cAMP by phosphorylation of a PDE - this is one of the possibilities addressed. We acknowledge the possibility that this could also be via the adenylate cyclase (see line 792).

2. PMID: 29030485 demonstrates clearly a link between cAMP signalling and PKG signalling, but does not demonstrate how this is mediated. The authors postulate that a cGMP-specific PDE is dysregulated given their observation that PDE2 is differentially phosphorylated in a constitutively inactive PKA mutant, however this was not validated experimentally. We and others (Moss *et al.*, 2022), however, demonstrate that PDE2 is cAMP-specific. This suggests that the model built by PMID: 29030485 requires revisiting. We acknowledge clearly in the text that Jia et al. have shown a link between cAMP and PKG signalling, and hypothesise that CDPK3's modulation of cAMP levels may affect this (this is in keeping with our phosphoproteomic data).

Moreover, the egress defect is not due to a low influx of calcium in the cytosol because when the ionophore A23187 was added to the CDPK3 mutant, its phenotype was not recovered. Rather, the defect may be due to the low or null activity of PKG that would activate PI4K to generate IP3 and DAG. The latter would be used as a substrate by DGK to generate PA that is involved in the secretion of micronemes and Toxoplasma egress. In this context, authors should evaluate the role of CDPK3 in the secretion of micronemes that is directly related to the egress of the parasite.

1. We agree with the reviewer on their point about calcium influx, and have already acknowledged in the text that the feedback loop does not control release of Ca^{2+} from internal stores as disruption of CDPK3 does not lead to a delay in Ca^{2+} release.

2. We agree, and clearly address in the text, that the egress defect could be due to altered PKG/phospholipid pathway signalling.

3. (Lourido, Tang and David Sibley, 2012; McCoy *et al.*, 2012) have both previously shown that microneme secretion is regulated by CDPK3. We therefore do not deem it necessary to repeat this experiment, but have made clearer mention of their findings in our writing.

When the Dcdpk3 mutant with BIPPO treatment was evaluated, it was observed that the parasite recovered the egress phenotype. It is concluded that CDPK3 could probably regulate the activity of cGMP-specific PDEs. CDPK3 could (in)activate them, or it could act on other proteins indirectly regulating the activity of these PDEs. Upon inactivation of PDEs, an increase in the cGMP level would activate PKG, which will, in turn, promote egress. From the data, it is not clear whether any phosphorylation by CDPK3 would activate or inactivate PDEs, and if so, then how (directly or indirectly). To reach unambiguous interpretation, authors should perform additional assays.
Response:

As mentioned previously, given the abundance of differentially regulated phosphosites, we do not believe that mutating individual sites on individual proteins is a worthwhile or realistic pursuit.

We clearly show systematic A23187-mediated phosphorylation of key signalling components in the PKA/PKG/PI-PLC/phospholipid signalling cascade, and demonstrate that several of these are CDPK3-dependent. We demonstrate that CDPK3 alters cAMP levels (and that the ΔCDPK3 egress delay in A23187 treated parasites is largely rescued following pre-treatment with a PKA inhibitor). We similarly demonstrate that A23187 treatment leads to phosphorylation of numerous PDEs, including the cAMP specific PDE2, and show that PDE2 knockout parasites show an egress delay following A23187 treatment. While PDE2 may not be directly regulated by CDPK3 (suggesting other Ca^{2+} kinases are also involved), these findings collectively demonstrate the existence of a calcium-regulated feedback loop, in which CDPK3 and PDE2 play a role (by regulating cAMP).

We acknowledge that we have not untangled every element of this feedback loop, and do not believe that it would be realistic to do so in a single study given the number of sites phosphorylated and pathways involved. We do believe, however, that we have shown clearly that the feedback loop exists - this in itself is entirely novel, and of significant importance to the field.

On a similar note, a possible experiment that can be done to improve the work would be to treat the CDPK3 mutant with BIPPO in conjunction with a calcium chelator (BAPTA-AM) to reveal, which proteins are phosphorylated prior to activation of the calcium-mediated cascades?

Response:

We agree that this would be an interesting experiment to carry out but would involve significant work. This could be pursued in another paper or project but is beyond the scope of this work.

The manuscript claims that PDE1, PDE7, PDE9 are cGMP specific, and BIPPO inhibits only cGMP-specific PDEs. All assays are performed with 1-10 micromolar cAMP and cGMP for 1h. There is no data showing the time, protein and substrate dependence. Given the suboptimal enzyme assays, authors should re-do them as suggested here. (1) Repeat the pulldown assay with a higher number of parasites (50-100 million) and measure the protein concentration. (2) Set up the PDE assay with saturating amount of cAMP and cGMP, which is critical if the PDE1,7,9 have a higher Km Value for cAMP (means lower affinity) compared to cGMP. An adequate amount of substrate and protein allows the reaction to reach the Vmax. Once you have re-determined the substrate specificity (revise Fig 5D), you should retest BIPPO (Fig 5E) in the presence of cAMP and cGMP. It is very likely that you would

find the same result as PDE9 and PfPDEβ (BIPPO can inhibit both cAMP and cGMP-specific PDE), as described previously

We have repeated our assay using the exact same conditions outlined by Moss *et al.* This involved using a similar number of parasites, a longer incubation time of 2 hours at a higher temperature (37ºC) and with a lower starting concentration of cAMP (0.1 uM). We demonstrate that we are able to recapitulate both the Moss *et al.* and Vo *et al.* (see Supp Fig. 7B). However, we noticed that these reactions were not carried out with saturating cAMP/cGMP concentrations, since all reactions had reached 100% completion at the end of the assay whereby all substrate was hydrolysed. We therefore believe that based on our original assay, as well as the new PDE1 timecourse that we have performed (Supp Fig. 7C), that PDEs 1, 7 and 9 display predominantly cGMP hydrolysing activity, with moderate cAMP hydrolysing activity.

We also repeated the BIPPO inhibition assay using the Moss *et al.* conditions, and still observe that the cGMP activity of PDE1 is the most potently inhibited of all 4 PDEs. We also see moderate inhibition of the cAMP activities of PDE1 and PDE9, suggesting that cAMP hydrolytic activity can also be inhibited. Interestingly, the cGMP hydrolytic activities of PDEs 7 & 9, which were previously inhibited using our original assay conditions, no longer appear to be inhibited. This is likely due to the longer incubation time, which masks the reduced activities of these two PDEs following treatment with BIPPO.

The authors did not identify any PKG substrate, which is quite surprising as cAMP signaling itself could impact cGMP. Authors should show if they were able to observe enhanced cGMP levels in BIPPO-treated sample (which is expected to stimulate cGMP-specific PDEs). The author mention their inability to measure cGMP level but have they analyzed cGMP in the positive control (BIPPO-treated parasite line)? Why have they focused only on CDPK3 mutant, whereas in their phosphoproteomic data they could see other CDPKs too? It could be that other CDPK-mediated signaling differs and need PKA/PKG for activation.

In the title, the authors have mentioned that there is a positive feedback loop between calcium release, cyclic nucleotide and lipid signaling, which is quite an extrapolation as there is no clear experimental data supporting such a positive feedback loop so the author should change the title of the paper.

Response:

1. As addressed in our previous response to the reviewer, PMID: 29030485 demonstrates clearly a link between cAMP signalling and PKG signalling, but does not confirm how this is mediated. The authors surmise that a cGMP-specific PDE is dysregulated (although the PDE hypothesised to be involved has since been shown to be cAMP-specific), but are similarly unable to detect changes in cGMP levels. This suggests that their model may be incomplete.

2. The BIPPO treatment experiment suggested by the reviewer was already included in the original manuscript (see Fig. 4D in original manuscript, now Fig. 4E). With BIPPO treatment we are able to detect changes in cGMP levels.

3. We did not deem it to be within the scope of this study to study every single other CDPK. We chose to study CDPK3, as its egress phenotype was of particular interest given its partial rescue following BIPPO treatment. We reasoned that its study may lead us to identify the signalling pathway that links BIPPO and A23187 induced signalling.

4. As addressed in greater detail in our response to reviewer #2, the fact that the feedback loop appears to stimulate egress implies that it is positive.

Minor Comments:

Materials & Methods

Explanation of parameters is not clear (Line #360-367). Phosphoproteomics with A23187 (8 micromolar) treatment in CDPK3-KO and WT, for 15, 30 and 60s at 37{degree sign}C incubation with DMSO control. Simultaneously passing the DR and CDPK3 dependency thresholds: CDPK3-dependent phosphorylation

Response: We have modified the wording to make this clearer as per the reviewer's suggestion.

Line #368: At which WT-A23187 timepoint did the authors identify 2408 DR-up phosphosites (15s, 30s or 60s)? Or consistently in all? It should be clarified?

Response: As already stated in the manuscript (see line 366 in original manuscript, now line 1047), phosphorylation sites were considered differentially regulated if at any given timepoint their log2FC surpassed the DR threshold.

A23187 treatment of the CDPK3-KO mutant significantly increased the cAMP levels at 5 sec post-treatment, but BIPPO did not show any change. The authors concluded that BIPPO presumably does not inhibit cAMP-specific PDEs. However, the dual-specific PDEs are known to be inhibited by BIPPO, as shown recently (https://www.biorxiv.org/content/10.1101/2021.09.21.461320v1). Authors do confirm that BIPPO-treatment can inhibit hydrolytic activity of PfPDEbeta for cAMP as well as cGMP (Line #612). Besides, it was shown in Fig 5E that BIPPO can partially though not significantly block cAMP-specific PDE2. The statements and data conflict each other under different subtitles and need to be reconciled. Elevation of basal cAMP level in the CDPK3 mutant indicates the perturbation of cAMP signaling, however BIPPO data requires additional supportive experiments to conclude its relation with cAMP or dual-specific PDE.

Response:

1. The manuscript to which the reviewer refers does not use BIPPO in any of their experiments. They show that continuous treatment with zaprinast blocks parasite growth in a plaque assay, but do not test whether zaprinast specifically blocks the activity of any of the PDEs.

2. Having repeated the PDE assay using the Moss *et al.* conditions (as outlined above), we are now able to recapitulate their findings, showing that PDEs 1, 7 and 9 can display dual hydrolytic activity while PDE2 is cAMP specific. As explained further above, we believe that our original set of experiments are more stringent than the Moss *et al.* conditions. To confirm this, we also performed an additional experiment, incubating PDE1 for varying amounts of time using our original conditions (1 uM cAMP or 10 uM cGMP, at room temperature). This revealed that PDE1 is much more efficient at hydrolysing cGMP, and only begins to display cAMP hydrolysing capacity after 4 hours of incubation.

3. We also measured the inhibitory capacity of BIPPO on the PDEs using the Moss *et al.* conditions. During the longer incubation time, it seems that BIPPO is unable to inhibit PDEs 7 and 9, while with the more stringent conditions it was able to inhibit both PDEs. We reasoned that since BIPPO is unable to inhibit these PDEs fully, the residual activity over the longer incubation period would compensate for the inhibition, eventually leading to 100% hydrolysis of the cNMPs. We also see that while the cGMP hydrolysing capacity of PDE1 is completely inhibited, its cAMP hydrolysing capacity is only partially inhibited. These findings and the fact that PDE2 is not inhibited by BIPPO are in line with our experiments where we measured [cAMP] and showed that treatment with BIPPO did not lead to alterations in [cAMP].

The method used to determine the substrate specificity of PDE 1,2,7 and 9 resulted in the hydrolytic activity of PDE2 towards cAMP, while the remaining 3 were determined as cGMP-specific. However, PDE1 and PDE9 have been reported as being dual-specific (Moss et al, 2021; Vo et al, 2020), which questions the reliability of the preferred method to characterize substrate specificity by the authors. It is also suggested to use another ELISA-based kit to double check the results.

Response:

As outlined above, we have repeated the assay using the conditions described by Moss *et al.* (lower starting concentrations of cAMP, 2 hour incubation period at 37ºC) and find that we are able to recapitulate the results of both Moss *et al.* and Vo *et al.*. However, using the Moss *et al.* conditions, the PDEs have hydrolysed 100% of the cyclic nucleotide, suggesting that these conditions are less stringent than the ones we used originally using higher starting concentrations of cAMP and incubating for 1 hour only at room temperature. With enzymatic assays it is always important to perform them at saturating conditions (as already suggested by the reviewer) and therefore we believe that our original conditions are more stringent than the results using the Moss *et al.* conditions.

Line #607-608: Authors found PDE9 less sensitive to BIPPO-treatment and concluded PDE2 as refractory to BIPPO inhibition; however, the reduction level of activity seems similar as seen in PDE9-BIPPO treated sample? This strong statement should be replaced with a mild explanation.

Response: We have tempered our wording as per the reviewer’s suggestion

Figures and legends:

The introductory model in Fig S1 is difficult to understand and ambiguous despite having it discussed in the text. For example, CDPK1 is placed, but only mentioned at the beginning, and the role of other CDPKs is not clear. In addition, the arrows in IP3 and PKG are confusing. The location of guanylate and adenylate cyclase is wrong, and so on... The figure should include only the egress-related signaling components to curate it. The illustration of host cell in orange color must be at the right side of the figure in connection with the apical pole of the parasite (not on the top). Figure legend should also be rearranged accordingly and citations of the underlying components should be included (see below).

Response: We have modified Supp Fig. 1 as per the suggestions of reviewer#2 and #3. We have now modified the localisations of the proteins and have also removed the lines showing the cross talk between pathways. We have also highlighted to the reader that this is only a model and may not represent the true localisations of the proteins, despite our best efforts.

In Figure 5D, would you please provide the western blot analysis of samples before and after pulling down to demonstrate the success of your immunoprecipitation assay. Mention the protein concentration in your PDE enzyme assay. Please refer to the M&M comments above to re-do the enzyme assays.

Response:

We have now included western blots for the pull downs of PDEs 1, 2, 7 and 9 (Supp Fig. 7A). We chose not to measure protein concentrations of samples since all experiments were performed using the same starting parasite numbers, and we do not see large differences in activities between biological replicates of the PDEs.

Figure legend 1C: Line #194: There is no red-dotted line shown in graph! Correct it!

Response: We have modified this.

Figure 4Gi-ii: Shouldn't it be labelled i: H89-treatment and ii: A23178, respectively instead of DMSO and H89? (based on the text Line #579).

Response: Our labelling of Fig. 4Gi-ii is correct as panel i parasites were pre-treated with DMSO, while panel ii parasites were pre-treated with H89. Subsequent egress assays on both parasites were then performed using A23187.

We have modified the figures to include mention of A23187 on the X axis, and modified the figure legend to clarify pre-treatment was performed with DMSO and H89 respectively.

Bibliography:

Line #57 and 58: Citations must be selected properly! Carruthers and Sibley 1999 revealed the impact of Ca2+ on the microneme secretion within the context of host cell attachment and invasion, not egress as indicated in the manuscript! Similar case is also valid for the reference Wiersma et al 2004; since the roles of cyclic

nucleotides were suggested for motility and invasion. Also notable in the fact that several citations describing the localization, regulation and physiological importance of cAMP and cGMP signaling mediators (PMID: 30449726 , 31235476 , 30992368 , 32191852 , 25555060 , 29030485) are either completely omitted or not appropriately cited in the introduction and discussion sections.

Response:

We have modified the citations as per the reviewer's suggestions. We now cite Endo *et al.*, 1987 for the first use of A23187 as an egress trigger, and Lourido, Tang and David Sibley, 2012 for the role of cGMP signalling in egress. We also cite all the GC papers when we make first mention of the GC. We have also removed the Howard *et al.*, 2015 citation (PMID: 25555060) when referring to the fact that BIPPO/zaprinast can rescue the egress delay of ΔCDPK3 parasites.

Grammar/Language

Line #31: After "cAMP levels" use comma

Response:

We have modified this.

36: Sentence is not clear. Does conditional deletion of all four PDEs support their important roles? If so, the role in egress of the parasite?

Response:

We have clarified our wording as per the reviewer's suggestion. We state that PDEs 1 and 2 display an important role in growth since deletion of either these PDEs leads to reduced plaque growth. We have not investigated exactly what stage of the lytic cycle this is.

40: "is a group involving" instead of "are"

Response:

We found no mention of "a group involving" in our original manuscript at line 40 or anywhere else in the manuscript, so we are unsure what the reviewer is referring to.

108: isn't it "discharge of Ca++ from organelle stores to cytosol"?

Response: We thank the reviewer for spotting this error. We have now modified this sentence.

120: "was" instead of "were"

Response: Since the situation we are referencing is hypothetical, then ‘were’ is the correct tense.

Reviewer #3 (Significance (Required)):

There is a significant amount of work that underlies this manuscript; however, from a conceptual viewpoint, the manuscript does not offer significant advancement over the current knowledge without functional validation of phosphoproteomics data. In terms of the mechanism, it is not clear whether and how lipid turnover and cAMP-PKA signaling control the egress phenotype (lack of a validated model at the end of this study).In a methodical sense, the work uses established assays, some of which require revisiting to reach robust conclusions and avoid misinterpretation.

Compare to existing published knowledge

A large body of work preceding this manuscript has indicated the crosstalk of cAMP, cGMP, calcium and lipid signaling cascades. This work provides a further refinement of the existing model. The article is quite interesting from a throughput screening point of view, but it clearly lacks the appropriate endorsement of the hits.

Response:

Please refer to our first response to reviewer #3 for our full rebuttal to these points. We respectfully disagree with the assessment that the work presented does not advance current knowledge.

Audience

Field specific (Apicomplexan Parasitology)

Expertise

Molecular Parasitology

References

Bailey, A. P. *et al.* (2015) 'Antioxidant Role for Lipid Droplets in a Stem Cell Niche of Drosophila', *Cell*. The Authors, 163(2), pp. 340–353. doi: 10.1016/j.cell.2015.09.020.

Bullen, H. E. *et al.* (2016) 'Phosphatidic Acid-Mediated Signaling Regulates Microneme Secretion in Toxoplasma Article Phosphatidic Acid-Mediated Signaling Regulates Microneme Secretion in Toxoplasma', *Cell Host & Microbe*, pp. 349–360. doi: 10.1016/j.chom.2016.02.006.

Dass, S. *et al.* (2021) 'Toxoplasma LIPIN is essential in channeling host lipid fluxes through membrane biogenesis and lipid storage', *Nature Communications*. Springer US, 12(1). doi: 10.1038/s41467-021-22956-w.

Endo, T. *et al.* (1987) 'Effects of Extracellular Potassium on Acid Release and Motility Initiation in Toxoplasma gondii', *The Journal of Protozoology*, 34(3), pp. 291–295. doi: 10.1111/j.1550-7408.1987.tb03177.x.

Flueck, C. *et al.* (2019) *Phosphodiesterase beta is the master regulator of camp signalling during malaria parasite invasion*, *PLoS Biology*. doi: 10.1371/journal.pbio.3000154.

Howard, B. L. *et al.* (2015) 'Identification of potent phosphodiesterase inhibitors that demonstrate cyclic nucleotide-dependent functions in apicomplexan parasites', *ACS Chemical Biology*, 10(4), pp. 1145–1154. doi: 10.1021/cb501004q.

Jia, Y. *et al.* (2017) ' Crosstalk between PKA and PKG controls pH -dependent host cell egress of Toxoplasma gondii ', *The EMBO Journal*, 36(21), pp. 3250–3267. doi: 10.15252/embj.201796794.

Katris, N. J. *et al.* (2020) 'Rapid kinetics of lipid second messengers controlled by a cGMP signalling network coordinates apical complex functions in Toxoplasma tachyzoites', *bioRxiv*. doi: 10.1101/2020.06.19.160341.

Lentini, J. M. *et al.* (2020) 'DALRD3 encodes a protein mutated in epileptic encephalopathy that targets arginine tRNAs for 3-methylcytosine modification', *Nature Communications*. Springer US, 11(1). doi: 10.1038/s41467-020-16321-6.

Lourido, S., Tang, K. and David Sibley, L. (2012) 'Distinct signalling pathways control Toxoplasma egress and host-cell invasion', *EMBO Journal*. Nature Publishing Group, 31(24), pp. 4524–4534. doi: 10.1038/emboj.2012.299.

Lunghi, M. *et al.* (2022) 'Pantothenate biosynthesis is critical for chronic infection by the neurotropic parasite Toxoplasma gondii', *Nature Communications*. Springer US, 13(1). doi: 10.1038/s41467-022-27996-4.

McCoy, J. M. *et al.* (2012) 'TgCDPK3 Regulates Calcium-Dependent Egress of Toxoplasma gondii from Host Cells', *PLoS Pathogens*, 8(12). doi: 10.1371/journal.ppat.1003066.

Moss, W. J. *et al.* (2022) 'Functional Analysis of the Expanded Phosphodiesterase Gene Family in Toxoplasma gondii Tachyzoites', *mSphere*. American Society for Microbiology, 7(1). doi: 10.1128/msphere.00793-21.

Stewart, R. J. *et al.* (2017) 'Analysis of Ca2+ mediated signaling regulating Toxoplasma infectivity reveals complex relationships between key molecules', *Cellular Microbiology*, 19(4). doi: 10.1111/cmi.12685.

Vo, K. C. *et al.* (2020) 'The protozoan parasite Toxoplasma gondii encodes a gamut of phosphodiesterases during its lytic cycle in human cells', *Computational and Structural Biotechnology Journal*. The Author(s), 18, pp. 3861–3876. doi: 10.1016/j.csbj.2020.11.024.
