# Supplementary material for: A positive feedback loop mediates crosstalk between calcium, cyclic nucleotide and lipid signalling in calcium-induced Toxoplasma gondii egress
Source: PLoS Pathog. 2022 Oct 20;18(10):e1010901. doi: 10.1371/journal.ppat.1010901 (PMC9624417; doi:10.1371/journal.ppat.1010901)
Supplement: Attachment — Submitted filename: Cover_letter_Nofal_revised.docx [file ppat.1010901.s019.docx]

London, 20.09.2022

Moritz Treeck, PhD

Group Leader

Tel: +44 (0)20 37962345

email: [Moritz.Treeck@crick.ac.uk](mailto:Moritz.Treeck@crick.ac.uk)

www.treecklab.org

Dear Mathieu,

Hereby we submit a revised version of our manuscript. As requested, we have made most of the suggested changes- which are highlighted in the revised manuscript with track changes.

- Modify the abstract and title to better reflect CDPK3 requirements and non-requirements in Toxoplasma egress. We have been thinking about ways doing this but found no satisfactory solution. We have mentioned the non-essential role of CDPK3 again in the introduction instead.

- Clarify whether induced cAMP levels are significantly increased upon A23187 treatment in the CDPK3 KO line compared with the reference line or whether the observed differences upon treatment reflect the higher basal levels. This may have important consequences in the observed absence of functional links between CDPK3 and studied PDEs. Done

- Mention in the main text that proteome changes have not been investigated but that specific sets of sites mapping are deferentially regulated compared to other site mapping on the same proteins pointing to specific changes. Done

- Show the GO term enrichment analyses with associated p-values in the figures. Done, now Figure 4D

- Add an intensity scale bar to Figure 1B. Done

- Indicate in the results section the temperature at which all PDE assays were performed (ie, with saturating and non-saturating concentrations of cyclic nucleotides), which may also explain the observed differences with Moss et al. Done

With best wishes,

Moritz (Treeck)

Senior Group Leader, The Francis Crick Institute
