# Supplementary material for: A positive feedback loop mediates crosstalk between calcium, cyclic nucleotide and lipid signalling in calcium-induced Toxoplasma gondii egress
Source: PLoS Pathog. 2022 Oct 20;18(10):e1010901. doi: 10.1371/journal.ppat.1010901 (PMC9624417; doi:10.1371/journal.ppat.1010901)
Supplement: Peer review history

# Author comment: Author response to previous submission

**DOI:** 10.1371/journal.ppat.1010901.r001

24 Aug 2022

Attachment Submitted filename: 121903_1_rebuttal_2437671_rgntg1.pdf Click here for additional data file.

# Editor report: Decision Letter 0

**DOI:** 10.1371/journal.ppat.1010901.r002

**Authors:** Dominique Soldati-Favre, Mathieu Brochet

9 Sep 2022

Dear Dr Treeck

Thank you very much for submitting your manuscript "A positive feedback loop mediates crosstalk between calcium, cyclic nucleotide and lipid signalling in Toxoplasma gondii" for consideration at PLOS Pathogens. Following your revisions to an initial round of 3 independent reviewers, your manuscript was discussed by three members of the editorial board who appreciated the attention to an important topic. Based on the revised version, we are likely to accept your manuscript for publication, providing that you further modify it according to some of the reviewer’s recommendations:

- Modify the abstract and title to better reflect CDPK3 requirements and non-requirements in Toxoplasma egress

- Clarify whether induced cAMP levels are significantly increased upon A23187 treatment in the CDPK3 KO line compared with the reference line or whether the observed differences upon treatment reflect the higher basal levels. This may have important consequences in the observed absence of functional links between CDPK3 and studied PDEs.

- Mention in the main text that proteome changes have not been investigated but that specific sets of sites mapping are deferentially regulated compared to other site mapping on the same proteins pointing to specific changes

- Show the GO term enrichment analyses with associated p-values in the figures

- Add an intensity scale bar to Figure 1B

- Indicate in the results section the temperature at which all PDE assays were performed (ie, with saturating and non-saturating concentrations of cyclic nucleotides), which may also explain the observed differences with Moss et al.

Please prepare and submit your revised manuscript within 30 days. If you anticipate any delay, please let us know the expected resubmission date by replying to this email.

When you are ready to resubmit, please upload the following:

[1] A letter containing a detailed list of your responses to all review comments, and a description of the changes you have made in the manuscript.

Please note while forming your response, if your article is accepted, you may have the opportunity to make the peer review history publicly available. The record will include editor decision letters (with reviews) and your responses to reviewer comments. If eligible, we will contact you to opt in or out

[2] Two versions of the revised manuscript: one with either highlights or tracked changes denoting where the text has been changed; the other a clean version (uploaded as the manuscript file).

Important additional instructions are given below your reviewer comments.

Thank you again for your submission to our journal. We hope that our editorial process has been constructive so far, and we welcome your feedback at any time. Please don't hesitate to contact us if you have any questions or comments.

Sincerely,

Mathieu Brochet

Associate Editor

PLOS Pathogens

Dominique Soldati-Favre

Section Editor

PLOS Pathogens

Kasturi Haldar

Editor-in-Chief

PLOS Pathogens

​orcid.org/0000-0001-5065-158X

Michael Malim

Editor-in-Chief

PLOS Pathogens

orcid.org/0000-0002-7699-2064

***********************

Figure Files:

While revising your submission, please upload your figure files to the Preflight Analysis and Conversion Engine (PACE) digital diagnostic tool, https://pacev2.apexcovantage.com. PACE helps ensure that figures meet PLOS requirements. To use PACE, you must first register as a user. Then, login and navigate to the UPLOAD tab, where you will find detailed instructions on how to use the tool. If you encounter any issues or have any questions when using PACE, please email us at figures@plos.org.

Data Requirements:

Please note that, as a condition of publication, PLOS' data policy requires that you make available all data used to draw the conclusions outlined in your manuscript. Data must be deposited in an appropriate repository, included within the body of the manuscript, or uploaded as supporting information. This includes all numerical values that were used to generate graphs, histograms etc.. For an example see here: http://www.plosbiology.org/article/info%3Adoi%2F10.1371%2Fjournal.pbio.1001908#s5.

Reproducibility:

To enhance the reproducibility of your results, we recommend that you deposit your laboratory protocols in protocols.io, where a protocol can be assigned its own identifier (DOI) such that it can be cited independently in the future. Additionally, PLOS ONE offers an option to publish peer-reviewed clinical study protocols. Read more information on sharing protocols at https://plos.org/protocols?utm_medium=editorial-email&utm_source=authorletters&utm_campaign=protocols

References:

Please review your reference list to ensure that it is complete and correct. If you have cited papers that have been retracted, please include the rationale for doing so in the manuscript text, or remove these references and replace them with relevant current references. Any changes to the reference list should be mentioned in the rebuttal letter that accompanies your revised manuscript. If you need to cite a retracted article, indicate the article’s retracted status in the References list and also include a citation and full reference for the retraction notice.

# Author comment: Author response to Decision Letter 0

**DOI:** 10.1371/journal.ppat.1010901.r003

22 Sep 2022

Attachment Submitted filename: Cover_letter_Nofal_revised.docx Click here for additional data file.

# Editor report: Decision Letter 1

**DOI:** 10.1371/journal.ppat.1010901.r004

**Authors:** Dominique Soldati-Favre, Mathieu Brochet

29 Sep 2022

Dear Dr. Treeck,

We are pleased to inform you that your manuscript 'A positive feedback loop mediates crosstalk between calcium, cyclic nucleotide and lipid signalling in calcium-induced Toxoplasma gondii egress.' has been provisionally accepted for publication in PLOS Pathogens.

Before your manuscript can be formally accepted you will need to complete some formatting changes, which you will receive in a follow up email. A member of our team will be in touch with a set of requests.

Please note that your manuscript will not be scheduled for publication until you have made the required changes, so a swift response is appreciated.

IMPORTANT: The editorial review process is now complete. PLOS will only permit corrections to spelling, formatting or significant scientific errors from this point onwards. Requests for major changes, or any which affect the scientific understanding of your work, will cause delays to the publication date of your manuscript.

Should you, your institution's press office or the journal office choose to press release your paper, you will automatically be opted out of early publication. We ask that you notify us now if you or your institution is planning to press release the article. All press must be co-ordinated with PLOS.

Thank you again for supporting Open Access publishing; we are looking forward to publishing your work in PLOS Pathogens.

Best regards,

Dominique Soldati-Favre

Section Editor

PLOS Pathogens

Dominique Soldati-Favre

Section Editor

PLOS Pathogens

Kasturi Haldar

Editor-in-Chief

PLOS Pathogens

​orcid.org/0000-0001-5065-158X

Michael Malim

Editor-in-Chief

PLOS Pathogens

orcid.org/0000-0002-7699-2064

***********************************************************

Dear Dr Treeck,

We are pleased to inform you that your manuscript ' has been provisionally accepted for publication in PLOS Pathogens.

Before your manuscript can be formally accepted you will need to modify your abstract to better detail the requirement of CDPK3 in the feedback loop as briefly discussed.

Best regards,

Mathieu Brochet

Reviewer Comments (if any, and for reference):

# Editor report: Acceptance letter

**DOI:** 10.1371/journal.ppat.1010901.r005

**Authors:** Dominique Soldati-Favre, Mathieu Brochet

17 Oct 2022

Dear Dr. Treeck,

We are delighted to inform you that your manuscript, "A positive feedback loop mediates crosstalk between calcium, cyclic nucleotide and lipid signalling in calcium-induced *Toxoplasma gondii* egress," has been formally accepted for publication in PLOS Pathogens.

We have now passed your article onto the PLOS Production Department who will complete the rest of the pre-publication process. All authors will receive a confirmation email upon publication.

The corresponding author will soon be receiving a typeset proof for review, to ensure errors have not been introduced during production. Please review the PDF proof of your manuscript carefully, as this is the last chance to correct any scientific or type-setting errors. Please note that major changes, or those which affect the scientific understanding of the work, will likely cause delays to the publication date of your manuscript. Note: Proofs for Front Matter articles (Pearls, Reviews, Opinions, etc...) are generated on a different schedule and may not be made available as quickly.

Soon after your final files are uploaded, the early version of your manuscript, if you opted to have an early version of your article, will be published online. The date of the early version will be your article's publication date. The final article will be published to the same URL, and all versions of the paper will be accessible to readers.

Thank you again for supporting open-access publishing; we are looking forward to publishing your work in PLOS Pathogens. 

Best regards,

Kasturi Haldar

Editor-in-Chief

PLOS Pathogens

​orcid.org/0000-0001-5065-158X

Michael Malim

Editor-in-Chief

PLOS Pathogens

orcid.org/0000-0002-7699-2064
